# A Global Overview of Diversity and Phylogeny of the Rust Genus *Uromyces*

**DOI:** 10.3390/jof8060633

**Published:** 2022-06-14

**Authors:** Ajay Kumar Gautam, Shubhi Avasthi, Rajnish Kumar Verma, Sushma, Mekala Niranjan, Bandarupalli Devadatha, Ruvishika S. Jayawardena, Nakarin Suwannarach, Samantha C. Karunarathna

**Affiliations:** 1 School of Agriculture, Abhilashi University, Mandi 175028, Himachal Pradesh, India; 2 School of Studies in Botany, Jiwaji University, Gwalior 474011, Madhya Pradesh, India; 3 Department of Plant Pathology, Punjab Agricultural University, Ludhiana 141004, Punjab, India; vermarajnish1985@gmail.com; 4 Department of Biosciences, Chandigarh University, Gharuan 140413, Punjab, India; smunihal@gmail.com; 5 Department of Botany, Rajiv Gandhi University, Rono Hills, Doimukh, Itanagar 791112, Arunachal Pradesh, India; neeru436@gmail.com; 6 Fungal Biotechnology Lab, Department of Biotechnology, School of Life Sciences, Pondicherry University, Kalapet 605014, Pondicherry, India; devadatha796@gmail.com; 7 Center of Excellence in Fungal Research, Mae Fah Luang University, Chiang Rai 57100, Thailand; ruvishika.jay@mfu.ac.th; 8 Research Center of Microbial Diversity and Sustainable Utilization, Chiang Mai University, Chiang Mai 50200, Thailand; 9 Center for Yunnan Plateau Biological Resources Protection and Utilization, College of Biological Resource and Food Engineering, Qujing Normal University, Qujing 655011, China; samanthakarunarathna@gmail.com

**Keywords:** phylogeny, *Pucciniomycotina*, rust fungi, taxonomy, *Uromyces*

## Abstract

*Uromyces* is the second-largest plant pathogenic rust genus, is responsible for numerous diseases, and has major effects on both agricultural and non-agricultural plants. The genus is generally characterized by its unicellular teliospores that help to characterize it and distinguish it from another important rust genus, *Puccinia*. In this study, a global overview of the diversity and distribution of *Uromyces* is presented based on both online and offline resources. The information obtained was analyzed for numerical and graphical summaries to provide the diversity and distribution of the genus by country and continent. Besides this, broad taxonomical aspects, a brief life cycle, and other comparative aspects on diversity and distribution were also provided. In addition, a phylogenetic analysis based on the ITS and nLSU DNA sequence data available in GenBank and published literature was performed to examine the intergeneric relationships of *Uromyces*. The results obtained revealed that the rust genus is found distributed over 150 countries, territories, and occupancies of the world on around 647 plant genera belonging to 95 plant families. Phylogenetic studies based on LSU and ITS sequence data revealed that *Uromyces* species are polyphyletic and require more DNA-based analyses for a better understanding of their taxonomic placement.

## 1. Introduction

*Uromyces* (Link) Unger, a genus of rust fungi, was proposed by Unger (1833). The genus contains several important plant pathogens, parasitizes in both monocots and dicots throughout the world, and affects a range of crops, causing a varying amount of yield loss annually, with losses being very severe in many cases. This genus shares synonymy with numerous genera of fungi such as *Alveomyces* Bubák., *Capitularia* Rabenh., *Coeomurus* Gray., *Dichlamys* Syd. & P. Syd., *Groveola* Syd., *Haplopyxis* Syd. & P. Syd., *Haplotelium* Syd., *Hypodermium* subgen. *Uromyces* Link., *Klebahnia* Arthur., *Nielsenia* Syd., *Poliotelium* Syd., *Poliotelium* Syd., *Pucciniola* L. Marchand., *Teleutospora* Arthur & Bisby., *Telospora* Arthur., *Trochodium* Syd. & P. Syd., and *Uromycopsis* Arthur. *Hypodermium* subgen. *Uromyces* Link 1816 has been considered the Basionym of this genus [1]. *Uromyces* is the second largest genus of rust fungus after *Puccinia* in the family *Pucciniaceae*, which contains more than 1568 epithets and more than 800 reported species [1,2,3,4,5]. This genus produces unicellular teliospores that help to characterize it and distinguish it from another important rust genus, *Puccinia*.

Species of *Uromyces* occur on a wide variety of plant hosts around the world. The most important ones are caused by *Uromyces* spp. that cause infections and damage to various agricultural crops such as alfalfa or lucerne rust (*Uromyces straitus* J. Schröt.), bean rust [*Uromyces appendiculatus* (Pers.) Unger], beet rust [*Uromyces betae* (Pers.) Tul.], carnation rust [*Uromyces dianthi* (Pers.) Niessl], chickpea rust (*Uromyces ciceris-aerietinus* Jacz.), clover rust (*Uromyces trofolii-repentis* Liro), and pea rust [*Uromyces pisi* (DC.) G.H. Otth.] [2]. The *Uromyces* species was recorded on the host plants belonging to the families *Asteraceae*, *Euphorbiaceae*, *Fabaceae*, *Liliaceae*, *Loranthaceae*, and *Poaceae,* and among these, *Loranthaceae* is known to be the most affected. However, the known distribution of most *Uromyces* species on *Loranthaceae* is restricted to Mexico and Central and South America [6].

The present manuscript is focused on providing a global overview of the diversity and distribution of the genus *Uromyces*. Based on the combined ITS and LSU DNA sequence data available in GenBank and published literature, phylogenetic analyses were performed to examine the intergeneric relationships of *Uromyces*. In addition, broad taxonomical aspects, a brief life cycle, and other comparative aspects of its diversity and distribution are also provided.

## 2. Taxonomy of the Genus *Uromyces*

The genus *Uromyces* belongs to the phylum *Basidiomycota*, the second-largest [7] phylum after *Ascomycota* in the kingdom *Fungi*; both phyla are considered higher fungi and share 97% of all fungal species [3,8,9,10,11]. Taxonomically, *Uromyces* contains rusts under the class *Pucciniomycetes* and order *Pucciniales*. Based on telial morphology, the spermogonial and aecial type, basidiospores, and uredinial characteristics, 13 rust families were proposed by Cummins and Hiratsuka [2]. However, Aime [12] and Aime and McTaggart [5] proposed several changes in basal systematics of rust fungi based on molecular studies along with morphological characteristics. This resulted in the introduction of many new families and the transmission of many genera and species. The Wijayawardene et al. [3] included 21 genera with *Uromyces* and 4961 species in the rust family *Pucciniaceae*. The recent higher-level classification for rust fungi provided recently by Aime and McTaggart [5] proposed the inclusion of 23 genera (including *Uromyces*) and 9 likely belonging to the family *Pucciniaceae*. The genus *Uromyces* was described by Unger in 1833 with *Uromyces appendiculatus* Pers. as the type species of the genus. *Uredo appendiculatus* Pers. was considered as a basionym for the type species, now regarded as *Uromyces appendiculatus* (Pers.) Link 1816. The detailed taxonomy of the genus *Uromyces* is described as follows: 

*Uromyces* (Link) Unger, Exanth. Pflanzen (Wien): 277 (1833):

= *Alveomyces* Bubák, Annln K.K. naturh. Hofmus. Wien 28 (1–2): 190 (1914);
= *Capitularia* Rabenh., Bot. Ztg. 9 (25): 449 (1851);
= *Coeomurus* Gray, Nat. Arr. Brit. Pl. (London) 1: 541 (1821);
= *Dichlamys* Syd. & P. Syd., Annlsmycol. 17(2/6): 105 (1920), (1919);
= *Groveola* Syd., Annlsmycol. 19 (3–4): 173 (1921);
= *Haplopyxis* Syd. & P. Syd., Annlsmycol. 17 (2/6): 105 (1920), (1919);
= *Haplotelium* Syd., Annlsmycol. 20 (3/4): 124 (1922);
= *Hypodermium* subgen. *Uromyces* Link, Mag. Gesell. naturf. Freunde, Berlin 8: 28 (1816), (1815);
= *Klebahnia* Arthur, Résult. Sci. Congr. Bot. Wien 1905: 345 (1906);
= *Mapea* Pat., Bull. Soc. Mycol. Fr. 22: 46 (1906);
= *Nielsenia* Syd., Annlsmycol. 19 (3–4): 171 (1921);
= *Nigredo* (Pers.) Roussel, Fl. Calvados, Edn 2: 47 (1806);
= *Peridipes* Buriticá & J.F. Hennen, RevtaAcad. Colomb. Cienc. Exact. Fís. Nat. 19 (no. 72): 50 (1994);
= *Poliotelium* Syd., Annlsmycol. 20 (3/4): 124 (1922);
= *Puccinella* Fuckel, Jb. nassau. Ver. Naturk. 15: 18 (1860);
= *Pucciniola* L. Marchand, Bijdr. Natuurk. Wetensch. 4: 47 (1829);
= *Rubigo* (Pers.) Roussel, Fl. Calvados, Edn 2: 46 (1806);
= *Teleutospora* Arthur & Bisby, Bull. Torrey bot. Club 48: 38 (1921);
= *Telospora* Arthur, Résult. Sci. Congr. Bot. Wien 1905: 346 (1906);
= *Trichobasis* Lév., in Orbigny, Dict. Univ. Hist. Nat. 12: 785 (1849);
= *Trochodium* Syd. & P. Syd., Annlsmycol. 17(2/6): 106 (1920) (1919);
= *Uredo* Pers., Syn. meth. fung. (Göttingen) 1: 214 (1801);
= *Uromycopsis* Arthur, Résult. Sci. Congr. Bot. Wien 1905: 345 (1906).

Classification:

*Pucciniaceae*, *Pucciniales*, *Pucciniomycetes*, *Pucciniomycotina*, *Basidiomycota*, *Fungi*

Type of species:

***Uromyces appendiculatus*** Pers., Observ. Mycol. (Lipsiae) 1: 17 (1796).

The characteristic one-celled teliospores of the genus *Uromyces* distinguish it from *Puccinia*, which has two-celled teliospores. *Spermogonia* subepidermal, Group V (type 4). *Aecia* subepidermal, erumpent, *Aecidium*-type (with peridium and catenuate, mostly verrucose aeciospores) or *Uredo*-type (with mostly echinulate aeciospores borne singly on pedicels). *Uredinia,* mostly the *Uredo* type, is subepidermal and erumpent and consists of echinulate urediniospores with various pores, borne singly on pedicels. Telia are subepidermal, erumpent, or remains covered by the epidermis with pedicellate, one-celled teliospores borne singly on pedicels with a mostly pigmented wall. Basidia is external in nature. The species of *Uromyces* produce macrocyclic and heteroecious modes of the life cycle, as well as many endocyclic, microcyclic, or autoecious species [2,5].

The phylogenetic analyses of different DNA loci revealed that *Uromyces,* along with the genus *Puccinia* in the family *Pucciniaceae*, is not monophyletic [5]. The generic name of *Uromyces* was recommended to be protected over *Uredo* [13]. *Uredo betae,* as one of the initially included species of this genus when described in 1801 by Persoon, is now considered a synonym of *Uromyces beticola*. Since *Uromyces appendiculatus* and *U. beticola* are regarded as congeneric, *Uromyces* and *Uredo* are synonyms. Previously, the name *Uredo* was used in cases where sexual morph was lacking and the species could not be described in the “correct” sexually typified genus. The reason why the name *Uromyces* was protected over *Uredo* is due to two major reasons: *Uredo* belongs to many different rust genera, and secondly, *Uromyces* is a commonly used generic name. The generic name *Uromyces* was already conserved over *Coeomurus* Link ex Gray 1821 and *Pucciniola* L. Marchand 1829 [13].

## 3. Biology, Pathogenicity, and Life Cycle of *Uromyces*

Rusts represent one of the largest groups of plant pathogens and have historically posed a major threat to farmers around the world. These diversified fungi are obligate biotrophic parasites distributed in all geographical areas on a wide range of wild and cultivated plants. The two major plant groups, cereals and legumes, suffer the most from rust infections [14]. The genus *Uromyces* consists of a number of plant pathogens responsible for many harmful plant diseases.

### 3.1. Disease Symptoms of Uromyces

**Figure 1:**
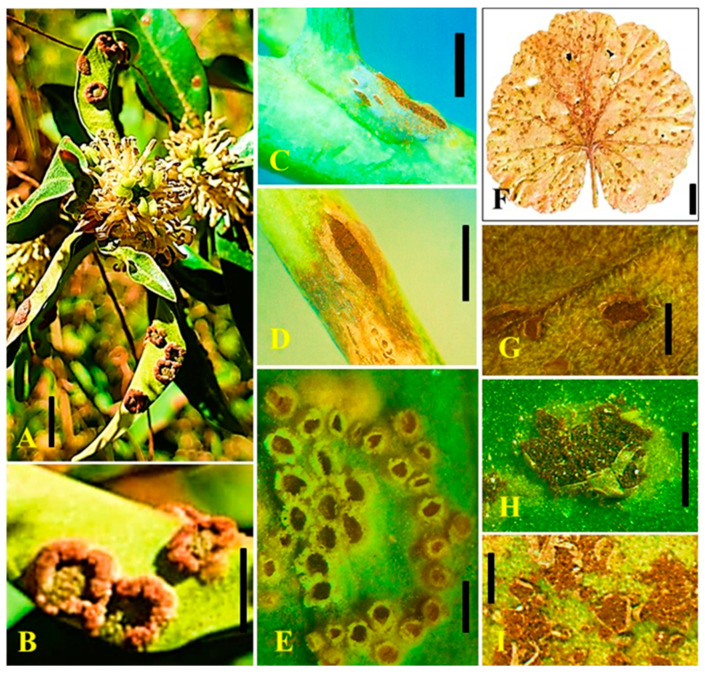
Disease symptoms of species of *Uromyces*. (**A**,**B**) *Uromyces ambiens* on *Buxus wallichiana*; (**C**–**E**) *Uromyces fabae* on *Pisum sativum*; (**F**,**G**) *Uromyces geranii* on *Geranium* sp.; (**H**,**I**) *Uromyces trifolii* on *Trifolium* sp. Scale bar: **A**–**I** = 1 mm.

*Uromyces,* as a pathogen, affects major hosts such as alfalfa (*Medicago sativa*), bean (*Phaseolus vulgaris*), carnation (*Dianthus caryophyllus*), chickpea (*Cicer arietinum*), clover (*Trifolium* sp.), and pea (*Pisum sativum*) belonging to plant families, *Fabaceae* and *Poaceae* [2,15]. The rust diseases caused by *Uromyces* spp. are characterized by numerous small, rust-like orange/yellow or brown pustules formed on infected plant tissues. *Uromyces,* as a plant pathogen, kills the diseased plant cells and forms a light yellow halo around diseased pustules. Dark brown to black-brown telia are usually formed by the rust fungi during infection. The severity of the disease causes a loss of photosynthetic area in infected plants, resulting in a reduction in overall plant performance. Since rust disease is affected by low temperatures and heavy rain in spring and autumn, this can affect the severity of the disease. The rust diseases caused by *Uromyces* are generally autoecious (one plant host) and macrocyclic (produce five spore stages in the life cycle) in nature [2]. The appearance of rust disease symptoms varies in different hosts. A single species of *Uromyces* can infect multiple hosts and cause variable symptoms. The rust on pea (*Pisum sativum*) caused by *Uromyces pisi* and *Uromyces viciae-fabae* produce minute, whitish, slightly raised spots that enlarge and rupture the epidermis to produce reddish-brown, irregular pustules on the stems, pods and lower surface of leaves. Initially, the pustules contain abundant powdery urediniospores, but eventually, they turn dark brown to black as overwintering teliospores are produced [16]. Similarly, *Uromyces viciae-fabae* is also reported to cause lentil rust with disease symptoms of circular yellowish-white pycnidia and aecial cups on leaflets, which later become oval to circular, brown uredial pustules; and dark brown to black, elongated telia [17]. Such variability in disease symptoms caused by different *Uromyces* species can also be observed in other crops (Figure 1).

### 3.2. Life Cycle

The species of rust genus *Uromyces* are generally macrocyclic in nature, i.e., exhibit all five spore forms known in *Pucciniales*. In addition, these fungi exhibit an autoecious life cycle, meaning that all spore forms are produced on a single host. However, the endocyclic or microcyclic nature with a heteroecious mode of the life cycle was observed in some species of *Uromyces* [2,5]. The general life cycle process of *Uromyces* spp. involves the germination of diploid teliospores in the spring with a metabasidium after overwintering on plant debris. The metabasidium produces four haploid basidiospores after meiosis. These basidiospores with two different mating types germinate and start infection by producing different infection structures on the surface of the host plant. Once an infection is established, the production of pycnia containing pycniospores of two mating types and receptive hyphae takes place. After, the spermatization of pycniospores of pycnia of different mating types and subsequent dikaryotization takes place in aecial primordia, along with a subsequent exchange.

**Figure 2:**
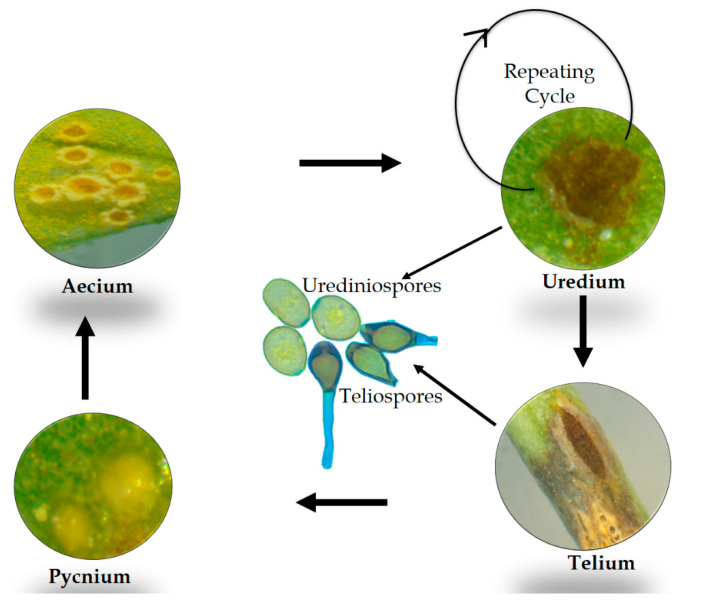
A general life cycle of rust genus *Uromyces*.

After the aecium is fully developed, the mature aeciospores infect the host surface by germinating and producing infection structures. Eventually, this infection leads to the production of uredia with urediospores. With the repeated infection of the host plant, these urediospores are produced in large quantities during the summer. Surprisingly, urediospores can disseminate thousands of kilometers with the help of the wind. When suitable hosts are found, these spores differentiate into telia, which ultimately produce unicellular diploid teliospores in the winter [17,18]. A general life cycle of the rust genus *Uromyces* is presented in Figure 2 below.

### 3.3. The Rusts as Classical Biocontrol Agents

*Uromyces* is the causal agent of rust disease on numerous agricultural, horticultural, and forest plantations. Rust fungi of this genus are considered a major economic threat due to possible yield losses from reduced production. Rust diseases on pea, beans, lentils, polyhouse flower crops, clover, and many more are some of the hosts infected by this genus. In addition to their identity as the causative agent of rust, the *Uromyces* species also shows potential for the biological control of various phytopathogenic fungi, weeds, etc. The biology and effectiveness of *Uromyces heliotropii* were studied by Hasan and Aracil [19], where they found the rust to effectively control an annual weed heliotrope (*Heliotropium europaeum* L.).

They evaluated the biocontrol potential of the rust in a greenhouse and field inoculation experiments and observed that the rust rapidly killed infected plants and reduced or prevented seed production. Similarly, Anderson et al. [20] conducted a study to evaluate the potential of three rusts naturally infecting Chilean needle grass (*Nassella neesiana*) in Argentina, *Uromyces pencanus*, *Puccinia graminella*, and *P. nassellae*, as biocontrol agents. They found *U. pencanus* to be most effective due to the damage it inflicts on its host in the field. A list of species of *Uromyces* studied as biocontrol agents is given in Table 1.

Rust fungi as biocontrol agents are mainly studied in their use against plant weeds. The biocontrol ability of some species of *Puccinia* was investigated, such as as *P. abrupta* var. *partheniicola* on different growth stages of *Parthenium hysterophorus* [27,28], *Puccinia arechavaletae* on *Cardiospermum grandiflorum* [29], *Puccinia komarovii* var. *glanduliferae* on *Impatiens glandulifera* [30], and *Prospodium transformans* on *Tecoma stans* var. *stans* [31]. A number of studies on *Uromyces* species have been carried out by a number of researchers (see Table 1), but the assessment of species from other rust genera has not yet been well established. Furthermore, the broad aspects of the rust fungi as biocontrol agents based on biochemical and molecular approaches still need to be explored.

## 4. Data Collection and Molecular Analysis

### 4.1. Data Collection and Compilation

This paper was compiled based on the information obtained from an extensive search of peer-reviewed publications, field guides, monographs, books, conference proceedings, project reports, and other offline and online resources. This information was updated as recently as December 2020. The information obtained was compiled firstly as a table depicting names of species of *Uromyces*, their hosts along with the family, the locality of occurrence, and the reference of scientific publication. The scientific names of the hosts and fungi were then cross-verified for scientific entities. The host name given in the original citation was sometimes changed to be consistent with the current taxonomy based on The Plant List (http://www.theplantlist.org; accessed on 20 April 2022). The names of rust fungi genus/species as reported in the cited publications were replaced by currently accepted names according to the websites MycoBank (www.mycobank.org; accessed on 20 April 2022) and Species Fungorum (www.speciesfungorum.org; accessed on 20 April 2022). Fungal Databases, US National Fungus Collections, ARS, USDA, an important online source of plant pathogens and their hosts, was also noted during the compilation [32]. An attempt was made to summarize all available literature on diversity and distribution of *Uromyces* spp.; only the most appropriate references were included in this study.

### 4.2. Analyses of Collected Data

After inserting the collected data into the primary database as a table, they were analyzed for numerical and graphical summaries. First, the information was analyzed by providing a comparative representation of the diversity and distribution of rust fungi (*Uromyces*) by country and continent. Thereafter, distribution patterns based on substrate types (herb, shrub, and tree) were constructed to understand the host preference of these rust fungi. In addition, the data of the host family were also presented. The publication indices of *Uromyces* spp. in terms of year, decade, and era are analyzed and presented in this paper. In addition, the references in other languages are translated into English so that the scientific community can understand them easily.

### 4.3. Molecular Data Analyzing

DNA sequence data of *Uromyces* species from the LSU and ITS rDNA were downloaded from GenBank and through earlier published literature. A checklist of molecular studies on *Uromyces* sp. along with the name of isolate and references were also prepared and presented in Table 2. The relevant publications on molecular analyses were also consulted [5,12,33,34]. Individual nucleotide sequences of LSU and ITS were aligned distinctly using MAFFT 7 (http://mafft.cbrc.jp/alignment/server/; accessed on 1 April 2022) [35], followed by manual checking and editing where necessary in BioEdit v. 7.0.9 [36]. The sequences of taxa containing poorly aligned portions, incomplete data, missing sequence data, and gaps were trimmed. The ITS and LSU sequences alignment was converted to NEXUS format (.nxs) using ClustalX 2.1 (http://www.clustal.org/clustal2/; accessed on 1 April 2022) for Phylogenetic Analysis Using PAUP (PAUP) analysis. The aligned LSU and ITS single-gene datasets and a concatenated dataset of LSU and ITS genes were analyzed with PAUP 4.0b10 [37]. These datasets were run after completing the program output tree in the Bootstrap.tre file. Maximum Likelihood bootstrap values greater than 60% are considered good bootstrap supports and are given above each node. Phylogenetic trees are visualized using the FigTree v1.4.0 program [38] and reorganized in Microsoft power point.

## 5. Distribution, Diversity, and Molecular Data Analysis of *Uromyces*

### 5.1. Phylogenetic Analyses

**Figure 3:**
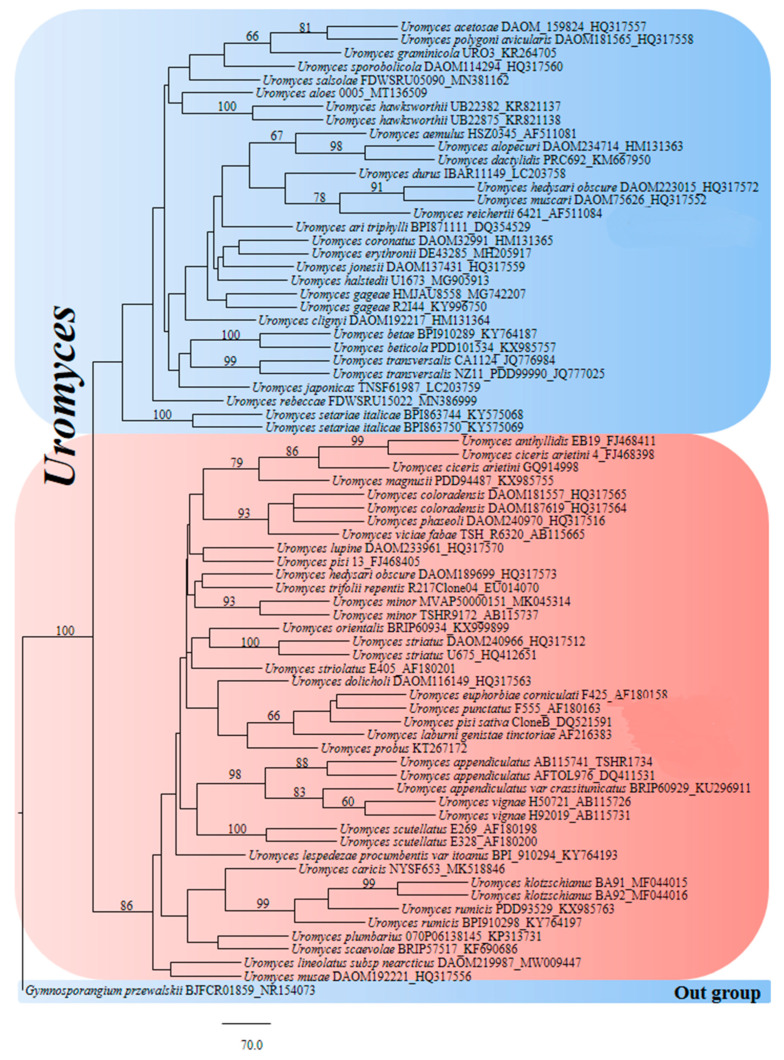
Phylogram generated from PAUP analysis of *Uromyces* species of ITS sequences. The scale bar indicates 70 changes and outgroup *Gymnosporangium przewalskii* BJFCR01859_NR154073. Bootstrap values of MP > 60% are given above branches, and 72 sequences are included in the phylogenetic analyses. The best maximum parsimony (MP) dataset consists of 394 total characters, of which 164 were constant, 156 parsimony-informative, and 74 parsimony-non-informative. The parsimony analysis of the data matrix showed a thousand equally parsimonious trees with a length of 917 steps in the first tree (CI = 0.408, RI = 0.702, RC = 0.286, HI = 0.592).

**Figure 4:**
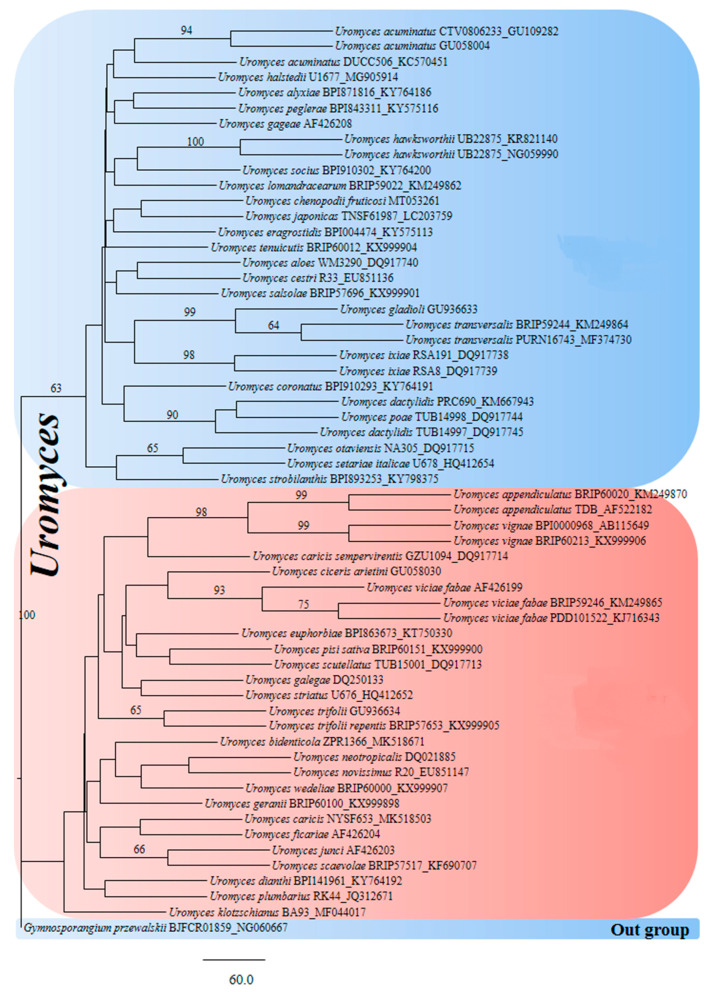
Phylogram generated from PAUP analysis of *Uromyces* species of LSU sequences. The scale bars indicate 60 changes and outgroup *Gymnosporangium przewalskii* BJFCR01859_NG060667. Bootstrap values of MP equal to or greater than 60% are given above branches, and 58 sequences are included in the phylogenetic analyses. The best maximum parsimony (MP) dataset consists of 873 total characters, of which 600 were constant, 125 parsimony-informative, and 148 parsimony-non-informative. The parsimony analysis of the data matrix showed 1000 equally parsimonious trees with a length of 625 steps in the first tree (CI = 0.550, RI = 0.708, RC = 0.389, HI = 0.450).

In the phylogenetic results, *Uromyces* were separated into two complexes in both ITS and LSU sequence data. Both complexes of ITS and LSU share many similar sequences. The incomplete sequences were mostly found in the *Uromyces* sequence dataset, e.g., ITS1 and 5.8S or ITS1, 5.8S complete, and ITS partial or 28S partially. Approximately 50% of the sequences had up to 300 nucleotides, while the remaining sequences had up to 800 nucleotides. Incomplete sequences can result in two complexes in a single genus. Therefore, complete gene sequences from ITS and LSU are needed to analyze these complex clades (Figure 3 and Figure 4).

### 5.2. Morphological Diversity and Distribution

The results compiled on diversity and distribution revealed that the rust genus *Uromyces* comprised a total of 1500 species that occurred worldwide as obligate parasitic fungi on vascular plants [3,4]. After combing through the different online databases [1,32,73], a total of 988 species were included in this paper. Similar to all fungi, its distribution shows great variations in different parts of the world. The tremendously changing climates across the world lead to diversified flora, resulting in a wide diversity and distribution of rust fungi. With regard to global diversity, it is pertinent to note here that *Uromyces* varies in diversity among countries and continents. These rust fungi generally show a macrocyclic nature and autoecious mode of the life cycle [2,5], which confirms their morphological diversity on specific hosts in particular regions. The broad host range in microcyclic and heteroecious life cycles was also found in *Uromyces* species. 

The genus *Uromyces* predominantly showed its great diversity in North America in comparison to other continents. Almost 834 (30%) species are described here, which is the highest among all continents. The diversity of the genus is known in other continents of the world as follows: Asia, with 633 (23%) species described; Europe, with 622 (23%) species; North America, with 321 (12%) species; Africa, with 313 (11%) described species; and Australia, with 32 (1%) species described. The genus appears to be well-represented in North American, Asian, and European counties. After dispersal by various modes, such as wind, water, or insect vectors, the propagules of rust fungi germinate and infect plant tissues of specific hosts. Entry of these pathogens takes place either by natural openings such as lenticels and stomata or by wounds or injuries caused by various physical agents [74].

**Figure 5:**
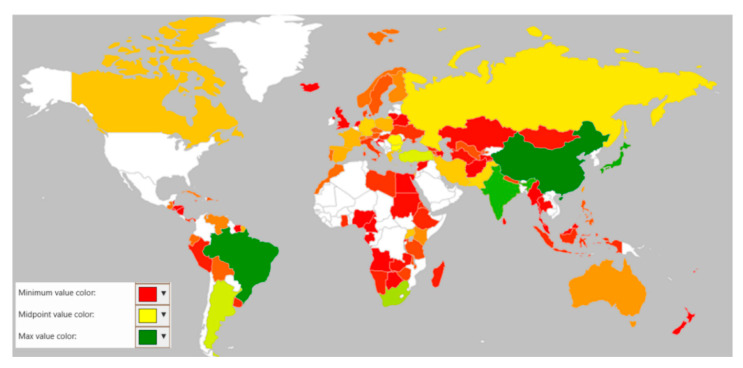
Heat map to show the global distribution and species richness of *Uromyces* spp.

**Figure 6:**
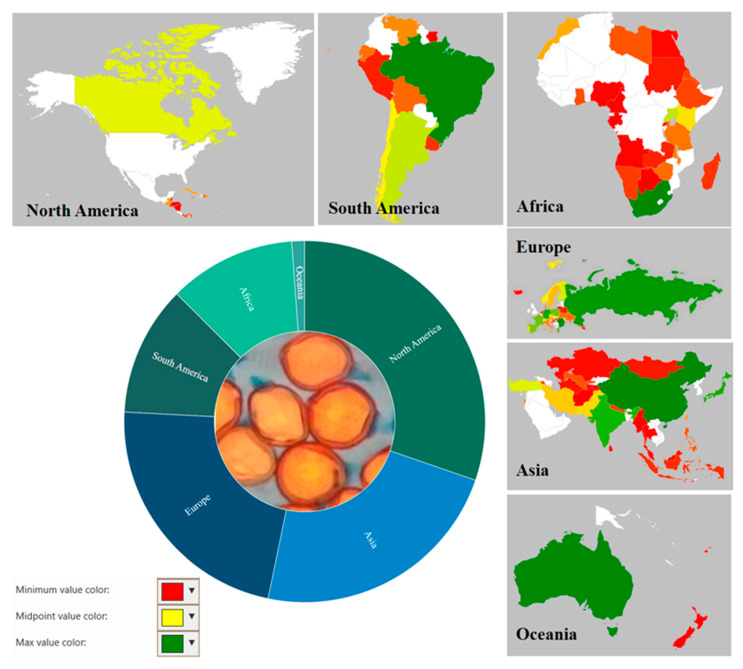
Heat maps to show the continental distribution and species richness of *Uromyces* spp.

Human anthropogenic activities also play an important role in the global distribution of these organisms. So far, *Uromyces* species have been found on every land on earth except Antarctica. To understand the distribution of the *Uromyces* species, we analyzed their distributions across continents and terrestrial ecoregions. More than 150 countries and territories or occupancies showed the distribution of this rust genus. Although only 73 sequences of ITS and nLSU *Uromyces* species were identified based on molecular characteristics, respectively, the majority of the species are still identified morphologically. This may impact the number and distribution pattern of the *Uromyces* species as molecular-based research on the rust fungi progress. In comparison to all continents, the highest distribution of 834 species of *Uromyces* was recorded over 66 regions of different countries and dependencies of North America. The distribution pattern observed in other continents was observed as follows: 633 species in 26 countries and dependencies of Asia; 622 species in 33 countries and dependencies of Europe; 321 species in 17 countries and dependencies of South America; 313 species in 27 countries and dependencies of Africa; and 37 species in 3 countries, islands, or dependencies of Oceania. The array of this global distribution of *Uromyces* species reveals their vast diversity and justifies its position as the second-largest genus of rust fungi (Figure 5 and Figure 6).

Distribution: America, Kazakhstan, India, Brazil, Syria, México, Russia, Cyprus, Ecuador, Uzbekistan, New Guinea, Cape Province, Algeria, Tunisia, Japan, Norway, China, Uganda, Hawaii, Argentina, Canada, Siberia, France, South Africa, Sri Lanka, Ethiopia, Colombia, Romania, Costa Rica, Israel, Chile, Switzerland, Ivory Coast, Uganda, Turkey, Kenya, Morocco, Britain, New Zealand, Malaysia, Philippines, Iran, Greece, Madagascar, Romania, Australia, Yunnan, Tadzhikistan, Pakistan, Zimbabwe, Myanmar, Namibia, Costa Rica, Taiwan, Nepal, Italy, Cuba, Afghanistan, Egypt, Iraq, Peru, Tibet, Croatia, Sudan, Ecuador, Jamaica, Sweden, Bolivia, Spain, Austria, Tunisia, Kenya, Norway, Kansas, Peru, Poland, and Venezuela.

### 5.3. Distribution Patterns of Rust Fungi (Uromyces) by Substrate Types

**Figure 7:**
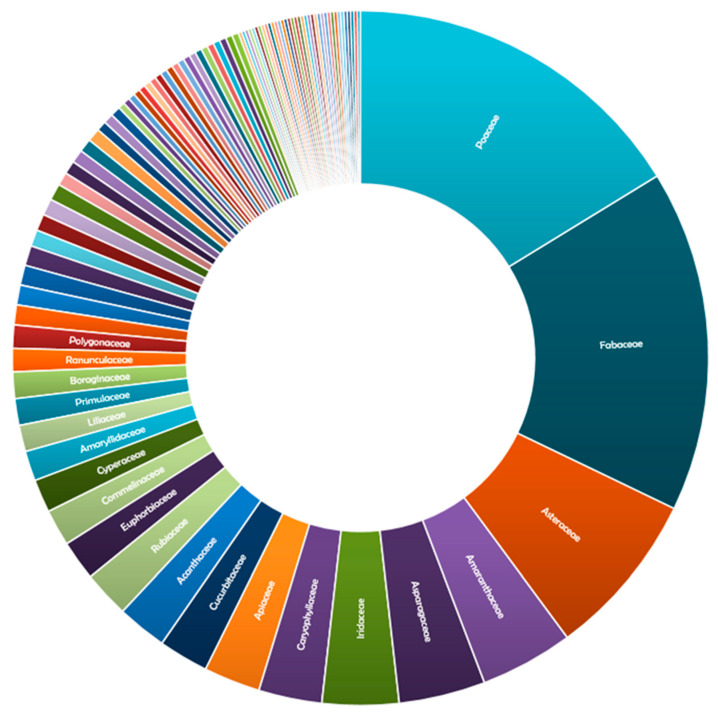
Family-wise comparison of genera of infected host plants.

*Uromyces*, being the second-largest rust genus after *Puccinia*, contains a number of important plant pathogens. The species of *Uromyces* attacks nearly all categories of plants and causes great damage to both plant and their products. *Uromyces* is a genus of rust fungi that infects both monocots and dicots throughout the world. Analyses of the available literature on host diversity of *Uromyces* revealed that a total of 647 plant genera belonging to 95 plant families were found to be infected by these rust fungi. *Uromyces* is particularly reported in plant families such as *Amaranthaceae*, *Apiaceae*, *Asparagaceae*, *Asteraceae*, *Caryophyllaceae*, *Cucurbitaceae*, *Cyperaceae*, *Euphorbiaceae*, *Fabaceae*, *Iridaceae*, *Poaceae*, and *Rubiaceae*. The highest occurrences of *Uromyces* spp. were observed in *Poaceae* and *Fabaceae,* with 105 and 103 infected host genera, respectively. A number of other families that have an infection in up to 50 plant genera have also been observed, including *Acanthaceae*, *Asparagaceae*, *Asteraceae*, *Caprifoliaceae*, *Cyperaceae*, *Euphorbiaceae*, *Fabaceae*, *Lamiaceae*, *Liliaceae*, *Loranthaceae*, *Oleaceae*, *Poaceae*, and *Polygonaceae*. A study on the diversity and distribution of *Uromyces* in India by Gautam and Avasthi [75] revealed that nearly 180 plant species belonging to 85 genera and 32 families were found to be infected with *Uromyces* spp. Among all families, *Fabaceae* and *Poaceae* were found to be the most infected with different species of *Uromyces*. A family-wise comparison of the genera of infected host plants is shown in Figure 7.

### 5.4. Global Host Range of Uromyces Species

**Figure 8:**
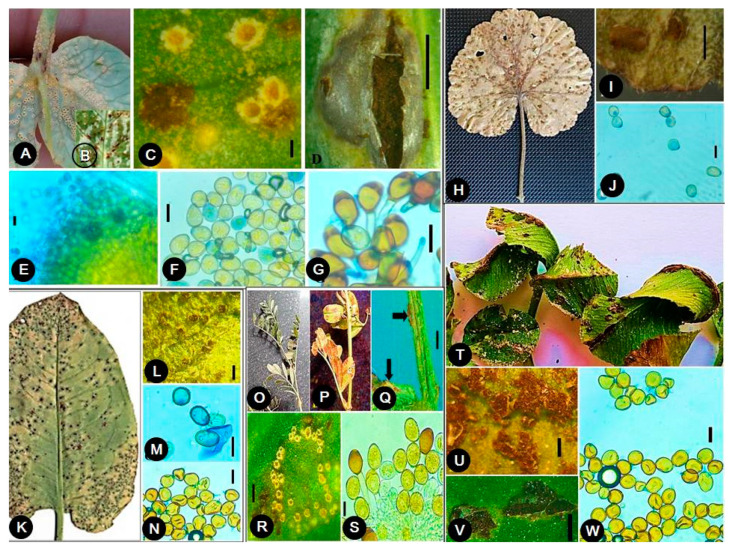
Occurrence of *Uromyces* species with some host plants. (**A**–**G**) *Uromyces fabae* on *Pisum sativum* (pea). (**A**) Aecia; (**B**,**C**) Uredia; (**D**) Telia; (**E**) Aeciospores; (**F**) Urediospores; (**G**) Teliospores; (**H**–**J**) *Uromyces geranii* on *Geranium* sp.; (**H**,**I**) Uredia; (**J**) Uredinospores; (**K**–**N**) *Uromyces rumicis* on *Rumex* sp; (**K**,**L**) Uredia; (**M**) Urediospores; (**N**) Teliospores; (**O**–**R**) *Uromycesviciae-fabae* on *Vicia faba*; (**O**–**Q**) Different rust sorus on natural host; (**R**) Aecia; (**S**) Urediospores and Teliospores; (**T**–**W**) *Uromyces trifolii* on *Trifolium* sp.; (**T**–**V**) Uredia and Telia; (**W**) Teliospores. Scale bars: **C**,**I**,**L**,**Q**,**R**,**U**,**V** = 1 mm; **D** = 0.5 mm; **E**,**F**,**G**,**J**,**M**,**N**,**S**,**W** = 20 µm.

The host range of *Uromyces* species showed their occurrence in 647 plant genera of 95 plant families. They are reported to be infected by various species of *Uromyces,* such as: *Abrus*, *Abuilon*, *Acacia*, *Acalypha*, *Acantholimon*, *Acanthophyllum*, *Acetosa*, *Achyranthes*, *Acidanthera*, *Acorus*, *Aconitum*, *Actinostemon*, *Adenostyles*, *Aegopogon*, *Aellenia*, *Aeluropus*, *Agropyron*, *Agrostis*, *Aira*, *Albizia*, *Albuca*, *Alhagi*, *Allium*, *Aloe*, *Alopecurus*, *Alstroemeria*, *Alysicarpus*, *Alyxia*, *Amblytropis*, *Ambrosia*, *Amischotolype*, *Amphicarpaea*, *Amphilophis*, *Anabasis*, *Anaphalis*, *Anarthrophyllum*, *Ancrumia*, *Andropogon*, *Aneilema*, *Anemone*, *Anguria*, *Anguria*, *Anisotome*, *Anomatheca*, *Anotis*, *Anthacanthus*, *Anthericum*, *Antholyza*, *Aphelandra*, *Apios*, *Apluda*, *Ardisia*, *Arenaria*, *Argyrolobium*, *Arisaema*, *Aristida*, *Armeria*, *Arnica*, *Artemisia*, *Arthrocnemum*, *Asclepias*, *Ascyrum*, *Aspalathus*, *Asperula*, *Aspilia*, *Aster*, *Astragalus*, *Astrebla*, *Atriplex*, *Atylosia*, *Avenella*, *Avenula*, *Babiana*, *Baccharis*, *Bahia*, *Baltimora*, *Bartholina*, *Basella*, *Bauhinia*, *Beckeropsis*, *Beckmannia*, *Bellevalia*, *Beloperone*, *Benedictella*, *Beta*, *Bidens*, *Blainvillea*, *Boissiera*, *Bolboschoenus*, *Boltonia*, *Bomarea*, *Bonaveria*, *Bongardia*, *Borreria*, *Bothriochloa*, *Bouvardia*, *Brachiaria*, *Bradburya*, *Briza*, *Brodiaea*, *Bromus*, *Bryzopyrum*, *Bufonia*, *Buforrestia*, *Bulbine*, *Bupleurum*, *Buxus*, *Cacalia*, *Caccinia*, *Cachrys*, *Cajanus*, *Calamagrostis*, *Calandrinia*, *Callicarpa*, *Callisia*, *Calopogonium*, *Caltha*, *Calycotome*, *Calyptridium*, *Camptosema*, *Campylotropis*, *Canavalia*, *Capillipedium*, *Caragana*, *Carex*, *Cassia*, *Caucalis*, *Cayaponia*, *Celosia*, *Celtis*, *Centropogon*, *Centrosema*, *Cerastium*, *Ceratocarpus*, *Ceratoides*, *Cestrum*, *Chaetobromus*, *Chaetochloa*, *Chaetolimon*, *Chamaecrista*, *Chamaecytisus*, *Chamaesyce*, *Chasmanthe*, *Chenopodium*, *Chesneya*, *Chiloglottis*, *Chionodoxa*, *Chloris*, *Chlorophytum*, *Chorizanthe*, *Cicer*, *Cicuta*, *Cimacoptera*, *Cirsium*, *Cissus*, *Cladostachys*, *Cladrastis*, *Claytonia*, *Cleome*, *Climacoptera*, *Clitoria*, *Clutia*, *Cnidoscolus*, *Cnidoscolus*, *Colchicum*, *Collomia*, *Cologania*, *Colutea*, *Combretum*, *Commelina*, *Commiphora*, *Convolvulus*, *Corallocarpus*, *Cordia*, *Coronilla*, *Cosmos*, *Crepis*, *Crocosmia*, *Crocus*, *Crotalaria*, *Cruckshanksia*, *Cucubalus*, *Cucumis*, *Cucurbita*, *Curculigo*, *Cyanotis*, *Cyathula*, *Cymbopogon*, *Cynosurus*, *Cyperus*, *Cypholepis*, *Cytisus*, *Dactylis*, *Dactyloctenium*, *Dalbergia*, *Danthonia*, *Deeringia*, *Dendroseris*, *Deschampsia*, *Desmodium*, *Desmostachya*, *Dianthus*, *Dichanthium*, *Dichromena*, *Dicliptera*, *Dierama*, *Digitaria*, *Diodia*, *Dipcadi*, *Dipogon*, *Discaria*, *Distichlis*, *Dodecatheon*, *Dolicholus*, *Dolichos*, *Doronicum*, *Dorycnium*, *Dorycnopsis*, *Dorystaechas*, *Doyerea*, *Dracaena*, *Drimiopsis*, *Echinocephalum*, *Edwardsia*, *Ehretia*, *Ehrharta*, *Eleocharis*, *Emex*, *Emmeorhiza*, *Enargea*, *Endymion*, *Engysiphon*, *Epicampes*, *Eragrostis*, *Eremopogon*, *Eremopyrum*, *Eriochloa*, *Eriogonum*, *Eriophyllum*, *Eriosema*, *Eriospermum*, *Erodium*, *Ervum*, *Erythrina*, *Erythronium*, *Eulophia*, *Euphorbia*, *Euryops*, *Excoecaria*, *Exotheca*, *Fatoua*, *Ferraria*, *Ferula*, *Festuca*, *Ficaria*, *Fimbristylis*, *Flemmingia*, *Fleurya*, *Floscopa*, *Freesia*, *Fritillaria*, *Gagea*, *Galactia*, *Galega*, *Galinsoga*, *Galium*, *Galphimia*, *Gamanthus*, *Gaudichaudia*, *Gaura*, *Gayophytum*, *Geissorhiza*, *Genista*, *Gentiana*, *Geranium*, *Gilliesia*, *Gladiolus*, *Glaux*, *Glyceria*, *Glycyrrhiza*, *Gnaphalium*, *Gomphrena*, *Gossypium*, *Gouania*, *Grindelia*, *Guizotia*, *Gurania*, *Gypsophila*, *Habrochloa*, *Haemanthus*, *Halenia*, *Halimodendron*, *Halocharis*, *Haloxylon*, *Hardenbergia*, *Hedysarum*, *Helianthus*, *Helichrysum*, *Helictotrichon*, *Heliopsis*, *Heliotropium*, *Helleborus*, *Helmontia*, *Hemarthria*, *Heracleum*, *Hesperantha*, *Heteranthera*, *Heteromorpha*, *Heteropogon*, *Hewittia*, *Hibiscus*, *Hippocrepis*, *Hippomarathrum*, *Holcus*, *Homogyne*, *Honckenya*, *Hookera*, *Hordeum*, *Hosackia*, *Houstonia*, *Hyacinthoides*, *Hyacinthus*, *Hydrocotyle*, *Hymenocarpos*, *Hyparrhenia*, *Hypericum*, *Hypoestes*, *Hypoxis*, *Hypsela*, *Indigofera*, *Inga*, *Ipomoea*, *Iresine*, *Isathne*, *Ixia*, *Jacobinia*, *Jacquemontia*, *Jasminum*, *Jatropha*, *Jaumea*, *Juncus*, *Juniperus*, *Justica*, *Kalidium*, *Karroochloa*, *Kochia*, *Koeleria*, *Krameria*, *Krascheninnikovia*, *Kummerowia*, *Kyllinga*, *Lablab*, *Laburnum*, *Lachenalia*, *Lantana*, *Lapeirousia*, *Lasiacis*, *Lasiocorys*, *Lathyrus*, *Ledenbergia*, *Leersia*, *Lembotropis*, *Lens*, *Leonotis*, *Leontice*, *Leopoldia*, *Leptochloa*, *Lerchenfeldia*, *Lespedeza*, *Leucocoryne*, *Ligularia*, *Lilium*, *Limonium*, *Lloydia*, *Loeselia*, *Lomandra*, *Loranthus*, *Lotononis*, *Lotus*, *Lupinus*, *Luzula*, *Lychnis*, *Lygeum*, *Lysimachia*, *Maackia*, *Maianthemum*, *Mallotus*, *Manettia*, *Manihot*, *Massonia*, *Mattiastrum*, *Medicago*, *Meibomia*, *Melandrium*, *Melanolepis*, *Melanthera*, *Melasphaerula*, *Medica*, *Melilotus*, *Melothria*, *Menyanthes*, *Mercurialis*, *Merremia*, *Messerschmidia*, *Microchloa*, *Microlespedeza*, *Microtis*, *Miersia*, *Mikania*, *Milium*, *Milla*, *Mimusops*, *Minuartia*, *Modecca*, *Moehringia*, *Momordica*, *Monocymbium*, *Montanoa*, *Moraea*, *Mucuna*, *Muehlenbeckia*, *Muhlenbergia*, *Mulinum*, *Muricauda*, *Musa*, *Muscari*, *Myosotis*, *Myrsine*, *Nassauvia*, *Nassella*, *Noaea*, *Nothoscordum*, *Nymphoides*, *Ocimum*, *Odontonema*, *Oenanthe*, *Oenothera*, *Olsynium*, *Onagra*, *Onobrychis*, *Ononis*, *Ophiorrhiza*, *Oreopolus*, *Ormosia*, *Ornithogalum*, *Ornithopus*, *Orobus*, *Orthosiphon*, *Oryctanthus*, *Oryza*, *Oxytropis*, *Pachylophus*, *Panicum*, *Parietaria*, *Paspalum*, *Paspalum*, *Passiflora*, *Pavonia*, *Peireskia*, *Peltandra*, *Pennisetum*, *Pentace*, *Pentaschistis*, *Peplis*, *Peracarpa*, *Perymenium*, *Petrorhagia*, *Petrosimonia*, *Peucedanum*, *Phaca*, *Phacelurus*, *Phalaris*, *Phaseolus*, *Phleum*, *Phlogacanthus*, *Phlox*, *Phoradendron*, *Phragmites*, *Phrygilanthus*, *Phthirusa*, *Physanthyllis*, *Phyteuma*, *Piptanthus*, *Pisum*, *Pittosporum*, *Plantago*, *Poa*, *Poinsettia*, *Poiretia*, *Poitea*, *Polemannia*, *Polemonium*, *Pollia*, *Polycnemum*, *Polygala*, *Polygonum*, *Polymnia*, *Polytrias*, *Polyxena*, *Pontederia*, *Pozoa*, *Prangos*, *Pratia*, *Primula*, *Priva*, *Pseudarthria*, *Pseuderanthemum*, *Psoralea*, *Psychotria*, *Pteracanthus*, *Pulicaria*, *Quinchamalium*, *Ranunculus*, *Rapanea*, *Rapanea*, *Ratibida*, *Rhinacanthus*, *Rhinopetalum*, *Rhynchospora*, *Rhynchosia*, *Ribes*, *Roepera*, *Romulea*, *Rosa*, *Rottboellia*, *Rudbeckia*, *Ruellia*, *Rumex*, *Rytidosperma*, *Sabinea*, *Salicornia*, *Salmea*, *Salpichroa Salsola*, *Sapium*, *Saururus*, *Scaevola*, *Schismus*, *Schizachyrium*, *Schizeilema*, *Scilla*, *Scirpus*, *Scleranthus*, *Scleria*, *Sclerochloa*, *Scribneria*, *Secale*, *Secamone*, *Securigera*, *Sedum*, *Selliera*, *Selysia*, *Senecio*, *Sesbania*, *Seseli*, *Sessea*, *Setaria*, *Sigesbeckia*, *Silene*, *Silphium*, *Simethis*, *Siphocampylus*, *Siphocampylus*, *Sisyrinchium*, *Sium*, *Smilacina*, *Smilax*, *Snowdenia*, *Solanum*, *Solaria*, *Solidago*, *Sophora*, *Sorghum*, *Sparaxis*, *Sparganium*, *Spartina*, *Spergularia*, *Spermacoce*, *Sphaeralcea*, *Sphenostylis*, *Sporobolus*, *Spraguea*, *Stellaria*, *Stenorrhynchus*, *Stipa*, *Strobilanthes*, *Strumaria*, *Struthanthus*, *Stylochaeton*, *Suaeda*, *Swainsona*, *Teramnus*, *Tessaria*, *Tetraena*, *Tetragonolobus*, *Thalictrum*, *Thapsia*, *Thelymitra*, *Themeda*, *Thermopsis*, *Thyrsacanthus*, *Tithymalus*, *Tournefortia*, *Toona*, *Tradescantia*, *Tragus*, *Triadenum*, *Trianosperma*, *Tribolium*, *Trichocline*, *Tricholaena*, *Trichoneura*, *Trichosanthes*, *Tricoryne*, *Trifolium*, *Trigonella*, *Tripogandra*, *Tripogon*, *Tripsacum*, *Trisetum*, *Triteleia*, *Tritonia*, *Trochomeriopsis Tulipa*, *Tylosema*, *Ulex*, *Uraria*, *Urginea*, *Urochloa*, *Valerianella*, *Veratrum*, *Verbascum*, *Veronica*, *Vicia*, *Vigna*, *Vilfa*, *Viola*, *Viscaria*, *Viscum*, *Vitis*, *Vossia*, *Vulpia*, *Watsonia*, *Wedelia*, *Wilbrandia*, *Wulffia*, *Zantedeschia*, *Zebrina*, *Zexmenia*, *Zigadenus*, *Zizania*, *Zizaniopsis,* and *Zygophyllum*. This compiled information depicts the broad host range of these rust fungi. The association of *Uromyces* species with some host plants is given in Figure 8, while detailed information on the diversity, host range, and distribution of *Uromyces* species is summarized in Table 3.

### 5.5. Endemic/Native Uromyces Species

**Figure 9:**
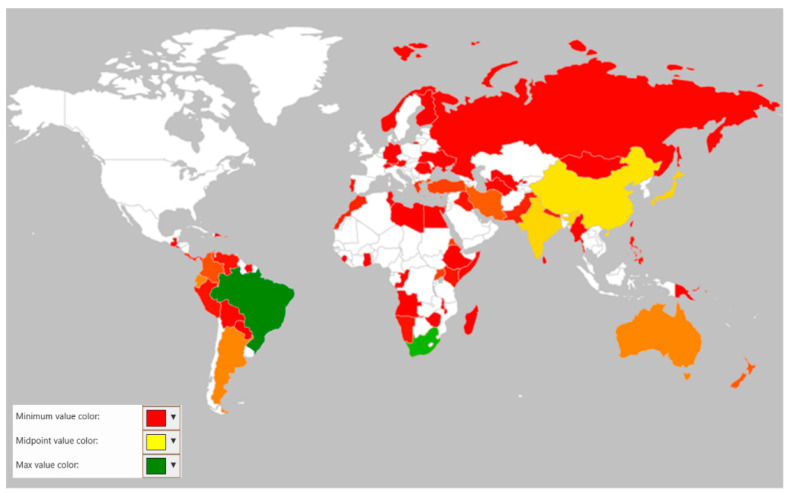
Geographical heat map showing distribution of endemic/native *Uromyces* species.

The distribution of rust fungi is governed by various ecological factors that affect their host range and endemism. As with other biotrophic pathogens, rust fungi are also found to be co-evolved with their host plants, which possibly justifies their host specificity and narrow host range. However, cross-infection in rust fungi is also observed, which opens up the need for further investigation of their host range. Among various rust fungi, the genus *Uromyces* also exhibited a broad host diversity among different terrestrial regions of the world. An analysis of data collected in the present study revealed the restriction of the *Uromyces* species towards a single host. More than 400 species of *Uormyces* were found to be endemic to more than 100 countries, provinces, or islands. The distribution of endemic species of *Uromyces* showed that the highest number of endemic species was found in Brazil (41 species) and South Africa (33 species). Other countries, such as China (18), Chile (18), Japan (17), India (17), México (15), France (12), Argentina (11), Australia (11), and Ecuador (11), showed a medium to low endemic distribution of these fungi. If we compare the continental distributions, it is clear from the geographical heat map that there is a big difference between the prevalence of endemic species in North and South America. In fact, South America showed the occurrence of the endemic *Uromyces* species. Similarly, Asia also holds a high number of species endemic to the different countries or regions in the continent. The distribution of the endemic species was found to be scattered compared to European countries; however, the number of endemic species in this region was comparatively high. Detailed information on the distribution of endemic or native *Uromyces* species is presented in Table 4 and Figure 9.

## 6. Discussion

*Uromyces* is the second-largest rust genus, the species of which are phytopathogenic to any category of plants, causing severe damage and reducing growth and yields. The present study provides literature-based complete information on this rust in a single compilation. In addition to being distributed worldwide on vascular plants, *Uromyces* species cause several damaging diseases on major agricultural crops such as alfalfa (*Medicago sativa*), bean (*Phaseolus vulgaris*), carnation (*Dianthus caryophyllus*), chickpea (*Cicer arietinum*), clover (*Trifolium* sp.), and pea (*Pisum sativum*). This study contributes to a better understanding of the taxonomy of these rust fungi in terms of their taxonomic placement, biology, pathogenicity, life cycle, diversity, and distribution. The information presented in this study helps to better understand all possible aspects of *Uromyces* in a single document.

The genus *Uromyces* is distributed globally on around 647 plant genera belonging to 95 plant families. *Poaceae* and Fabaceae are the most affected families, with the occurrence of more than 100 species of *Uromyces*. However, these fungi infect about 95 species; their occurrence on *Poaceae* and *Fabaceae* reflects the specificity of these rusts to grasses and legumes. In addition to host diversity, the distribution of species of *Uromyces* exhibited a wide range across the globe. The distribution of this genus extends to over 150 countries, territories, and occupancies of the world. In its continental diversity distribution, North America is followed by Asia, Europe, South America, Africa, and Oceania, respectively. A large variation in the geographical distribution along with the vast diversity of hosts demonstrated the impact of significantly changing climatic zones on rust fungi. Besides this, more than 400 *Uromyces* species are endemic to more than 100 countries, provinces, and islands. This may be due to the climatic conditions and precise distribution of hosts. Available studies on global diversity and distribution are rare; however, regional descriptions are available. A checklist of rust fungi of New Zealand provided by Mckenzie [313] reported the occurrence of 31 species of *Uromyces*. Similarly, Bahcecioglu and Kabaktepe [80] reported 74 species from Turkey, while Afshan and Khalid [536] reported 15 species of the grass family *Poaceae* from Pakistan. In India, 97 species of *Uromyces* have been reported on various hosts [75]. A total of 61 *Uromyces* species were reported from Portugal, whereas about 91 were from Iran [430]. The occurrence of species of *Uromyces* reported from different countries also supports the broad diversity of these fungi on a wide range of hosts.

Although 988 species of *Uromyces* investigated in the present study are found all over the world, only 73 species are known to have DNA sequence data. As in the case of other rust fungi, the species of *Uromyces* are also difficult to culture, which may be one of the major factors behind the reduced availability of molecular data. In addition, the isolation of DNA directly from rust fungi present on a natural host and then its sequencing is not simple or easy, which also affects the molecular studies of these fungi. Phylogenetic studies based on LSU and ITS sequence data revealed that *Uromyces* species are polyphyletic taxa and required more DNA-based analyses for a better understanding of their taxonomic placement. The polyphyletic nature of *Uromyces* species was also confirmed by Aime and McTaggart [5] in their study to propose a higher ranking classification for rust fungi, with notes on genera. Similarly, this was also proposed by Gautam et al. [34] during their study on Indian *Pucciniales* with the description of the taxonomic outline, including important descriptive notes. Overall, the present study proposes the requirement of fresh collections of *Uromyces* species and their molecular characterization to generate molecular data so that their phylogenetic relationships can be explained more precisely. The development of a universal digital platform exclusively for global rust fungi should be developed for the benefit of researchers working on this specific group of fungi.

## 7. Conclusions

Being the second-largest plant pathogenic rust genus, *Uromyces* showed a great variation with respect to its diversity and distribution. After a complete analysis of information gathered in the present study, it was concluded that the species of rust genus *Uromyces* are distributed globally. Their distribution has been reported in over 150 countries and territories or occupancies of the world. However, the genus *Uromyces* predominantly showed its great diversity in North America in comparison to other continents. Approximately 647 plant genera belonging to 95 plant families are reported to be affected by these rust pathogens. Apart from this, the endemic nature of this genus is also revealed, which concluded that more than 400 species of *Uormyces* are found to be endemic in more than 100 countries. The biocontrol nature of some species of *Uormyces* is also elucidated in this study. Moreover, analyses of LSU and ITS sequence data revealed the polyphyletic nature of species of *Uromyces.* Further DNA-based analyses of rust disease caused by *Uromyces* are still required to develop a better understanding of their taxonomic placement.

## Figures and Tables

**Table 1:** List of species of *Uromyces* with biocontrol potential.

Species of *Uromyces*	Target Organism (Plants)	Reference
*Uromyces rumicis*	*Rumex ckpus*	[21]
*Uromyces galegae*	*Galega officinalis*	[22]
*Uromyces heliotropii*	*Heliotropiu meuropaeum*	[19]
*Uromyces pencanus*	*Nassella neesiana*	[20,23,24,25]
*Uromyces pisi-sativi*	*Cytisus scoparius*	[26]
*Uromyces pisi-sativi*	*Chamaecytisus palmensis*	[26]
*Uromyces pisi-sativi*	*Lupinus polyphyllus*	[26]

**Table 2:** GenBank and voucher/culture collection accession numbers of *Uromyces* species included in the phylogenetic study.

Taxa	Isolate Name	GenBank Accession No.		References
		ITS	LSU	
*Uromyces acetosae*	DAOM 159824	HQ317557	HQ317557	[39]
*Uromyces acuminatus*	DUCC506	‒	KC570451	[40]
	CT-V080623-3	GU109282	‒	[41]
*Uromyces aemulus*	HSZ0345	AF511081	AF511081	[42]
*Uromyces aloes*	2020-6-28-0005	MT136509	‒	Unpublished
	WM 3290	‒	DQ917740	[43]
*Uromyces alopecuri*	DAOM 234714	HM131363	HM131363	[44]
*Uromyces alyxiae*	BPI 871816	‒	KY764186	Unpublished
*Uromyces anthyllidis*	EB19	FJ468411	‒	[45]
*Uromyces ari-triphylli*	BPI 871111	DQ354529	DQ354528	[12]
*Uromyces betae*	BPI910289	KY764187	‒	Unpublished
*Uromyces beticola*	PDD:101534	KX985757	KX985757	[46]
*Uromyces bidenticola*	ZP-R1366	‒	MK518671	Unpublished
*Uromyces caricis-sempervirentis*	GZU 10-94	‒	DQ917714	Unpublished
*Uromyces chenopodii-fruticosi*	K(M)107793	‒	MT053261	[47]
*Uromyces ciceris-arietini*	BPI 879192	FJ468398	GU058030	[48]
	‒	GQ914998	‒	[49]
*Uromyces clignyi*	DAOM 192217	HM131364	‒	[44]
*Uromyces coloradensis*	DAOM 181557	HQ317565	‒	[39]
	DAOM187619	HQ317564	‒	[39]
*Uromyces coronatus*	DAOM 32991	HM131365	‒	[44]
	BPI 910293	‒	KY764191	Unpublished
*Uromyces dactylidis*	PRC692	KM667950	‒	[50]
	TUB 14997	‒	DQ917745	[43]
*Uromyces dianthi*	BPI 141961	‒	KY764192	Unpublished
*Uromyces dolicholi*	DAOM 116149	HQ317563	‒	[39]
*Uromyces durus*	IBAR11149	LC203758	‒	[51]
*Uromyces eragrostidis*	BPI004474	‒	KY575113	Unpublished
*Uromyces erythronii*	DE-soo-43285	MH205917	MH205917	[52]
*Uromyces euphorbiae*	F425	AF180158	‒	[53]
	BPI 863673	‒	KT750330	Unpublished
*Uromyces ficariae*	WM 1398	‒	AF426204	[33]
*Uromyces gageae*	HMJAU:8558	MG742207	MG742207	[54]
	U242	DQ250133	DQ250133	[55]
*Uromyces geranii*	BRIP 60100	‒	KX999898	[56]
*Uromyces gladioli*	‒	‒	GU936633	[57]
*Uromyces graminicola*	URO 3	KR264705	‒	[58]
*Uromyces halstedii*	U1673	MG905913	‒	[59]
*Uromyces hawksworthii*	UB:22382	KR821137	‒	[60]
	UB:22875	KR821138	‒	
	UB:22875	‒	NG059990	
*Uromyces hedysari-obscuri*	DAOM 189699	HQ317573	‒	[39]
	DAOM 223015	‒	HQ317572	
*Uromyces ixiae*	RSA191	‒	DQ917738	[43]
	RSA8	‒	DQ917739	Unpublished
*Uromyces japonicus*	TNS:F:61987	LC203759	LC203759	[51]
*Uromyces jonesii*	DAOM 137431	HQ317559	HQ317559	Unpublished
*Uromyces junci*	GZU 11-98	‒	AF426203	[33]
*Uromyces klotzschianus*	BA91	MF044015	‒	[61]
	BA92	MF044016	‒	
	BA93	‒	MF044017	
*Uromyces lespedezae-procumbentis*	BPI 910294	KY764193	‒	Unpublished
*Uromyces lomandracearum*	BRIP:59022	‒	KM249862	[62]
*Uromyces lupini*	DAOM 233961	HQ317570	HQ317570	[39]
*Uromyces magnusii*	PDD:94487	KX985755	‒	[46]
*Uromyces minor*	MVAP50000151	MK045314	‒	Unpublished
*Uromyces musae*	DAOM 192221	HQ317556	‒	[39]
*Uromyces muscari*	DAOM 75626	HQ317552	HQ317552	[39]
*Uromyces neotropicalis*	MCA2533	‒	DQ021885	[63]
*Uromyces novissimus*	R20	‒	EU851147	[57]
*Uromyces orientalis*	BRIP 60934	KX999899	‒	[56]
*Uromyces otaviensis*	NA 305	‒	DQ917715	[43]
*Uromyces peglerae*	BPI 843311	‒	KY575116	Unpublished
*Uromyces pisi-sativa*	‒	DQ521591	‒	Unpublished
	BRIP 60151	‒	KX999900	[56]
*Uromyces plumbarius*	070P06138145	KP313731	‒	[64]
	RK44	‒	JQ312671	[65]
*Uromyces polygoni-avicularis*	DAOM 181565	‒	HQ317558	[39]
*Uromyces probus*	BPI 893209	KT267172	‒	[66]
*Uromyces rebeccae*	FDWSRU 15-022	MN386999	MN386999	[47]
*Uromyces reichertii*	6421	AF511084	AF511084	[42]
*Uromyces rumicis*	PDD:93529	KX985763	‒	[46]
	BPI 910298	‒	KY764197	Unpublished
*Uromyces salsolae*	FDWSRU 05-090	MN381162	MN381162	[47]
*Uromyces scaevolae*	BRIP:57517	KF690686	KF690686	[67]
*Uromyces scutellatus*	E328	AF180200	AF180200	[53]
*Uromyces setariae-italicae*	BPI 863744	KY575068	‒	Unpublished
*Uromyces setariae-italicae*	BPI 863750	‒	KY575069	Unpublished
*Uromyces socius*	BPI 910302	‒	KY764200	Unpublished
*Uromyces sporobolicola*	DAOM 114294	HQ317560	HQ317560	[39]
*Uromyces striatus*	U-675	HQ412651	‒	[68]
	DAOM 240966	HQ317512	‒	[39]
*Uromyces striolatus*	E405	AF180201	AF180201	[53]
*Uromyces strobilanthis*	BPI 893253	‒	KY798375	Unpublished
*Uromyces tenuicutis*	BRIP 60012	‒	KX999904	[56]
*Uromyces transversalis*	NZ11 PDD-99990	JQ777025	‒	Unpublished
	CA1124	JQ776984	‒	Unpublished
	PUR:N16743	‒	MF374730	[69]
*Uromyces trifolii*	‒	‒	GU936634	[57]
*Uromyces trifolii-repentis*	R217	EU014070	‒	[70]
	BRIP 57653	‒	KX999905	[56]
*Uromyces viciae-fabae*	TSH_R6320	AB115665	‒	[71]
	WM 1365	‒	AF426199	[33]
*Uromyces vignae*	H92019	AB115731	‒	[72]
	H50721	AB115726	‒	
	BRIP 60213	‒	KX999906	[56]
	BRIP 60213	‒	KX999906	
*Uromyces wedeliae*	BRIP 60000	‒	KX999907	[56]

**Table 3:** Diversity, host range, and distribution of species of *Uromyces*.

Species	Host	Locality	Reference
*Uromyces abbreviatus* Arthur	*Psoralea purshii* and *P. physodes*	California, Idaho (Western USA), Nevada, and Washington (USA)	[76,77,78]
*Uromyces acantholimonis* Syd. & P. Syd.	*Acantholimon libanoticum*	Afghanistan, Iran, Israel, Kazakhstan, Pakistan, and Turkey	[79,80,81]
*Uromyces acetosae* J. Schröt.	*Acetosa pratensis*, *Rumex acetosa*, *R. acetosella*, *R. acetoselloides*, *R. alpestris*, and *R. scutatus*	Bulgaria, Czech Republic, China, Czechoslovakia, Finland, Germany, Iceland, México, Norway, Poland, Portugal, Romania, Spain, Sweden, Russia, Taiwan, Turkey, and the United Kingdom	[80,82,83,84]
*Uromyces achrous* Syd. & P. Syd.	*Dalbergia sissoo*	India, Pakistan	[85,86]
*Uromyces aconiti* Fuckel	*Aconitum apetalum*, *A. barbatum*, *A. carmichaelii*, *A. delavayi*, *A. delavayi*, *A. lycoctonum*, *A. macrorhynchum*, *A. monticola*, *A. umbrosum*, *A. umbrosum*, and *A. volubile*	China and Japan	[87,88,89,90,91]
*Uromyces aconiti-lycoctoni* (DC.) G. Winter	*Aconitum apetalum*	California, Finland, Romania, and Spain	[78,92]
*Uromyces acori* T.S. Ramakr. & Rangaswami	*Acorus calamus*	India, China, Japan, and Thailand	[93,94,95,96,97,98,99]
*Uromyces actinostemonis* H.S. Jacks. & Holw.	*Actinostemon concolor*	Brazil	[100,101,102,103]
*Uromyces acuminatus* Arthur	*Arenaria lateriflora*, *Collomia linearis*, *Dodecatheon pulchellum*, *Honckenya peploides*, *Lysimachia ciliata*, *Maianthemum canadense*, *Phlox glaberrima*, *P. pilosa*, *Polemonium reptans*, *Smilacina stellate*, *Spartina alterniflora*, *S. alterniflora*, *S. cynosuroides*, *S. gracilis*, *S. patens*, and *S. pectinata*	Alberta, Bulgaria, California, Canada, Colorado, Delaware, Florida, Indiana, Iowa, Maine, Maryland, Massachusetts, Michigan, Montana, New Hampshire, New Jersey, New York, North America, North Dakota, Nova Scotia, Ontario, Quebec, Rhode Island, Saskatchewan, Wisconsin, and Wyoming	[41,104]
*Uromyces acutatus* Fuckel	*Gagea bohemica* and *G. villosa*	Germany	[105]
*Uromyces adelphicus* Syd.	*Milium trichopodium*	Syria	[106]
*Uromyces aecidiiformis* (F. Strauss) C.C. Rees	*Fritillaria pallidiflora*, *F. pineticola*, *F. ussuriensis*, *Lilium bulbiferum*, and *L. candidum*	China, Greece, Germany, Norway, Sweden, and the United Kingdom	[91,105,107,108,109]
*Uromyces aegopogonis* Dietel & Holw.	*Aegopogon cenchroides*, *A. geminiflorus*, *A. gracilis*, and *A. tenellus*	México	[106,110]
*Uromyces aeluropodinus* Tranzschel	*Aeluropus littoralis*	Ukraine	[111]
*Uromyces aeluropodis-repentis* Nattrass	*Aeluropus repens*	Cyprus, Turkey, and Ukraine	[80,106]
*Uromyces aemulus* Arthur	*Allium acuminatum*, *A. brevistylum*, *A. brandegei*, *A. douglasii*, *A. gooddingii*, *A. tolmiei*, and *A. validum*	Arizona, California, Colorado, Canada, Idaho, Nevada, Utah, Oregon, Washington, and Wyoming	[112,113,114,115,116]
*Uromyces affinis* G. Winter	*Alstroemeria aurantiaca*, *A. caryophyllaea*, *A. inodora*, *A. isabellana*, *A. nemorosa*, *Curculigo scorzoneraefolia*, *Hypoxis decumbens*, *H. erecta*, and *H. hirsuta*	Brazil, Colombia, Dominica, Dominican Republic, Florida, Missouri, Mississippi, and Puerto Rico	[102,117,118,119,120,121,122,123,124,125]
*Uromyces agnatus* Arthur	*Jatropha stimulosa*	Florida	[112]
*Uromyces agropyri* Barclay	*Agropyron* sp.	India	[126]
*Uromyces agrostidis* (Gonz. Frag.) A.L. Guyot	*Agrostis capillaris* and *Ranunculus repens*	Europe	[39]
*Uromyces aimeae* Berndt	*Cucurbitaceae*	Ecuador	[127]
*Uromyces airae-flexuosae* Ferd. & Winge	*Aira flexuosa*, *Avenella flexuosa*, *Deschampsia beringensis*, *Deschampsia flexuosa*, and *Lerchenfeldia flexuosa*	Bulgaria, Czech Republic, Czechoslovakia, Denmark, Finland, Germany, Poland, and Russia	[82,83,106,128,129,130,131,132,133,134,135]
*Uromyces albidum* Kirgizb.	*Acanthophyllum pungens*	Uzbekistan	[106]
*Uromyces albiziae* Henn.	*Albizia procera*	Indonesia	[136]
*Uromyces albucae* Kalchbr. & Cooke	*Albuca altissima*, *A. aurea*, *A. minor*, *A. canadensis*, *A. juncifolia*, and *A. wakefieldii*	Kenya, Malawi, South Africa	[137,138,139,140,141,142]
*Uromyces albus* (Clinton) Dietel & Holw.	*Vicia americana* and *V. linearis*	California and Nebraska	[78,143]
*Uromyces algeriensis* P. Syd. & Syd.	*Scilla obtusifolia* and *Scilla* sp.	Algeria, Cyprus, and Tunisia	[144,145]
*Uromyces alhagi* Szemb.	*Alhagi camelorum* and *A. sparsifolium*	Russia and Uzbekistan	[146]
*Uromyces allii-monanthi* Y. Harada	*Allium monanthum*	Japan	[147]
*Uromyces allii-sibirici* Gjaerum	*Allium sibiricum*	Norway	[148,149]
*Uromyces allii-victorialis* Liou & Y.C. Wang	*Allium fistulosum*, *A. macrostemon*, and *A. victorialis*	China	[90]
*Uromyces aloes* (Cooke) Magnus	*Aloe* spp.	India, Japan, and South Africa	[43,133,139,150,151,152,153,154]
*Uromyces alopecuri* Seym.	*Alopecurus aequalis*, *A. amurensis*, *A. arundinaceus*, *A. arundinaceus*, *A. geniculatus*, *A. japonicas*, *A. pratensis*, *Ranunculus sceluratus*, *R. sieboldii*, and *R. vernyi*	China, Colorado, Japan, Iowa, Minnesota, Nebraska, Texas, Turkey, and Wyoming	[80,92,96,155,156]
*Uromyces alpestris* Tranzschel	*Euphorbia cyparissias*	Bulgaria, Germany, Spain, and Switzerland	[82,157,158,159,160]
*Uromyces alsinis* Tranzschel	*Minuartia hamata* and *M. meyeri*	Turkey	[80,161]
*Uromyces alstroemeriae* (Dietel) Henn.	*Alstroemeria revoluta*, *A. aurantiaca*, *A. caryophyllaea*, *A. inodora*, *A. ligtu*, *A. isabellana*, *A. nemorosa*, and *A. subrosulacea*	America, Argentina, Brazil, and Chile	[143,162,163,164,165,166,167]
*Uromyces alysicarpi* Wakef. & Hansf.	*Alysicarpus glumaceus*, *A. monilifer*, *A. vaginalis*, and *A. violaceus*	India, Malawi, Uganda, South Africa, and Zambia	[138,139,141,168,169,170,171,172]
*Uromyces alyxiae* Arthur	*Alyxia oliviformis*	Hawaii	[173,174,175,176]
*Uromyces ambiens* Cooke	*Buxus sempervirens* and *B. wallichiana*	India and Pakistan	[177,178]
*Uromyces ambiguus* (DC) Lév.	*Allium ampeloprasum*, *A. angulosum,* *A. atroviolaceum*, *A. babingtonii*, *A. descendens*, *A. fistulosum*, *A. rotundum, A. schoenoprasum*, *A. sibiricum*, *A. ursinum*, *A. vineale*, and *A. waldsteinii*	Bulgaria, Finland, Germany, Greece, Japan, Poland, Romania, Ukraine, and the United Kingdom	[82,105,133,135,145,179]
*Uromyces americanus* Speg.	*Cicuta bulbifera*, *Hydrocotyle bonariensis*, *H. umbellata*, *Oenanthe sarmentosa*, *Scirpus americanus*, *S. acutus*, *S. californicus*, *S. lacustris*, *S. olneyi*, *S. validus*, *Sium cicutifolium*, and *Sium suave*	Alabama, Argentina, Canada, California, Delaware, Indiana, Nebraska, Puerto Rico, Texas, Uruguay, Virgin Islands, West Indies, and Wisconsin	[180,181,182,183,184,185]
*Uromyces amoenus* Syd. & P. Syd.	*Anaphalis alpicola*, *A. busua, A. contorta*, *A. margaritacea*, and *Gnaphalia margaritacea*	Canada, California, China, Idaho, Japan, Michigan, Montana, Nepal, Oregon, Russia, South Dakota, and Washington	[79,94,186,187,188,189,190]
*Uromyces amphidymus* Syd. & P. Syd.	*Glyceria acutiflora*, *G. borealis, G. fluitans*, and *G. septentrionalis*	America, Indiana, New Jersey, Rhode Island, and Wisconsin	[79,191,192,193]
*Uromyces amphilophis-insculptae* T.S. Ramakr., Sriniv. & Sundaram	*Amphilophis insculpta*	India	[194]
*Uromyces amurensis* Kom.	*Maackia amurensis*, *M. floribunda*, *M. hupehensis*, *M. tashiroi*, and *M. reticulata*	China, Japan, Korea, and Russia	[92,94,96,108,109]
*Uromyces anabasis* Kazenas	*Anabasis aphylla*	China	[195]
*Uromyces anagyridis* Roum.	*Anagyris foetida*, *A. mongolicus*, *A. nanus*, *Piptanthus concolor*, and *P. nepalensis*	China, Cyprus, Greece, Iraq, Israel, Spain, and Turkey	[80,81,96,108,109,196,197,198]
*Uromyces andropogonis* Tracy	*Andropogon elliottii*, *A. emersus*, *A. glomeratus*, *A. perforatus*, *A. ternarius*, *A. saccharoides*, *A. virginicus*, *Sorghum halepense*, *Viola papilionacea*, *V. pedata*, *V. pedata*, *V. striata*, and *V. tricolor*	Alabama, Arkansas, Bolivia, Carolina, Chile, Columbia, Connecticut, Delaware, Ecuador, Florida, India, Indiana, Illinois, Jersey, Kentucky, Louisiana, Maryland, Mississippi, Missouri, New North Georgia, New York, Ohio, Tennessee, Texas, and Virginia	[171,172,199,200,201,202,203,204,205]
*Uromyces andropogonis-annulati* Syd., P. Syd. & E.J. Butler	*Andropogon annulatus*, *A. abyssinicus*, *A. brevifolius*, *A. brevifolius*, *A. hirtiflorus*, *A. ischaemum*, *A. longipes*, *A. multinervis*, *A. pilosellus*, *A. platyphyllus*, *A. schottii*, *Bothriochloa insculpta*, *B. ischaemum*, *B. pertusa*, *Cymbopogon giganteus*, *Dichanthium annulatum*, *D. aristatum*, *D. caricosum*, *D. nodosum*, *Exotheca abyssinica*, *Hemarthria altissima*, *Hyparrhenia hirta*, *Monocymbium ceresiiforme*, and *Themeda triandra*	Barbados, Cuba, Ethiopia, Guatemala, India, Kenya, Malawi, Mauritius, México, New Guinea, Pakistann, Philippines, Uganda, Sierra Leone, Sudan, Tunisia, and West Indies	[85,87,88,122,139,168,171,172,201,206,207,208,209]
*Uromyces anguriae* H.S. Jacks. & Holw.	*Anguria warmingiana*, *Gurania pycnocephala*, *Helmontia cardiophylla*, and *Wilbrandia verticillata*	Brazil and French Guiana	[127,210,211,212]
*Uromyces anomathecae* Cooke	*Anomatheca cruenta*	South Africa	[123,138,169,213]
*Uromyces anotidis* Petch	*Anotis richardiana*	Sri Lanka	[214]
*Uromyces anotidis-monospermatis* T.S. Ramakr. & Sundaram	*Anotis monosperma*	India	[215]
*Uromyces anthacanthi* H.S. Jacks.	*Anthacanthus spinosus*	Puerto Rico, Virgin Islands, and West Indies	[125,184,216,217,218]
*Uromyces anthemophilus* Vestergr.	*Bauhinia longifolia* and *Bauhinia* sp.	Mato Grosso	[102,219]
*Uromyces antholyzae* Syd. & P. Syd.	*Antholyza abyssinica*	Ethiopia and South Africa	[138,142,169]
*Uromyces anthyllidis* (Grev.) J. Schröt.	*Anagyris* spp., *Astragalus* spp., *Benedictella* spp., *Coronilla* spp., *Dorycnium* spp., *Hedysarum* spp., *Hippocrepis* spp., *Lathurus* spp., *Lotus* spp., *Lupinus* spp., *Medicago* spp., *Ononis* spp., *Trifolium* spp., *Trigonella* spp., and *Vicia* spp.	Algeria, Armenia, Austria, Belarus, Bulgaria, Cyprus, France, Germany, Greece, Hungary, Iran, Itlay, Kyrgyzstan, Libya, Morocco, Palestine, Poland, Portugal, Romania, Spain, Switzerland, Syria, Tunisia, Turkey, Ukraine, Ukraine, and Yugoslavia	[81,220,221,222]
*Uromyces antiguanus* Cummins	*Desmodium orbiculare* and *Desmodium* sp.	Guatemala (USA) and México	[171,172,223,224,225]
*Uromyces antioquiensis* Mayor	*Rhynchospora polyphylla* and *R. nervosa*	Colombia	[119,226,227]
*Uromyces antipae* Săvul. & O. Săvul.	*Rosa lutea*	Romania	[228]
*Uromyces aphelandrae* Syd.	*Aphelandra pectinata*	Costa Rica	[218,224,229]
*Uromyces apiosporus* Hazsl.	*Primula minima* and *Primula suffrutescens*	Austria, Bulgaria, Germany, Poland, Romania, and California	[78,82,135,230,231]
*Uromyces apludae* Syd., P. Syd. & E.J. Butler	*Apluda mutica*, *Indigofera linifolia*, and *I. cordifolia*	India, Guinea, Pakistan, and Philippines	[85,208,232,233]
*Uromyces appelianus* Gassner	*Passiflora foetida* and *Cayaponia* sp.	Brazil, India, and Uruguay (USA)	[127,211,212,234]
*Uromyces appendiculatus* (Pers.) Link	*Amphicarpaea* spp., *Cajanus* sp., *Dolichos* spp., *Lablab* sp., *Phaseolus* spp., and *Vigna* spp.	Worldwide	[92,195,235,236,237,238]
*Uromyces appendiculatus* var. *azukicola* Hirata	*Phaseolus angularis*	Japan	[239]
*Uromyces aquiriensis* Berndt	*Cucurbitaceae*	Acre (Israel)	[127]
*Uromyces araucanus* Dietel & Neger	*Senecio otites*	La Araucania (Chile)	[162,240]
*Uromyces archerianus* Arthur & Fromme	*Chloris elegans*	Africa, China, Kenya, Malawi, and México (USA)	[108,109,110,171,172,241,242,243,244,245]
*Uromyces arenariae* Tranzschel	*Arenaria graminea, A. gypsophiloides*, and *A. serpyllifolia*	Armenia, Romania, and Turkey	[80,161,246]
*Uromyces arenariae-grandiflorae* Mayor	*Arenaria saponarioides*	Turkey	[80]
*Uromyces argaeus* Maire	*Rumex tuberosus*	Asia	[247]
*Uromyces argutus* F. Kern	*Spartina alterniflora* and *S. glabra*	Florida	[106,242,248]
*Uromyces argyrolobii* Doidge	*Argyrolobium amplexicaule* and *Sesbania* sp.	KwaZulu-Natal and Zimbabwe (South Africa)	[138,249,250]
*Uromyces ari-triphylli* (Schwein.) Seeler	*Arisaema* spp., *Peltandra virginica*, and *Zantedeschia* sp.	Britain, California, Canada, Florida, Indiana, Illinois, Iowa, Minnesota, Mississippi, México, Washington, and Virginia	[185,187,251,252,253,254,255,256]
*Uromyces arizonicus* Tracy & Galloway	*Eriogonum racemosum*	Arizona (USA)	[257]
*Uromyces armeriicola* Speg.	*Armeria chilensis*	North, Central, and South America, and West Indies	[120,125,258,259,260,261,262,263,264,265]
*Uromyces asclepiadis* Cooke	*Asclepias* spp.	Argentina, Brazil, Bolivia, Colombia, Cuba, California, Florida, Maine, Peru, Puerto Rico, México, and Texas	[100,118,119,123,124,262,266]
*Uromyces asperulae* McAlpine	*Asperula conferta* and *A. oligantha*	Australia	[267,268]
*Uromyces aspiliae* H.S. Jacks. & Holw.	*Aspilia phyllostachya*, *Aspilia* sp., and *Wedelia saltensis*	Argentina and Rio de Janeiro (Brazil)	[102,210,269]
*Uromyces aspiliellus* Vienn. -Bourg.	*Aspilia latifolia*	Ivory Coast	[270]
*Uromyces aspiliicola* Cummins	*Aspilia asperifolia*, *A. africana*, *A. kotschyi*, *Guizotia* sp., and *Wedelia* sp.	Malawi, Sudan, Tanzania, and Uganda	[168,271,272,273,274,275]
*Uromyces astragali-alopecuri* Gjaerum	*Astragalus alopecurus*	Turkey	[276]
*Uromyces astragali-atropilosuli* Gjaerum	*Astragalus atropilosulus* var. *bequaertii*	Kenya	[276]
*Uromyces astragalicola* Henn.	*Astragalus adsurgens*	North Dakota and Utah	[277,278]
*Uromyces astragali-pseudoutrigeris* Gjaerum	*Astragalus pseudoutriger*	Turkey	[276]
*Uromyces atlanticus* Guyot & Malençon	*Hippocrepis scabra*	Morocco	[279]
*Uromyces atriplicis* McAlpine	*Atriplex confertifolia*, *A. paludosa*, *A. semibaccata*, and *A. vesicaria*	Australia, Britain, and Colorado	[280,281,282]
*Uromyces atropidis* Tranzschel	*Atropidis distantis*	Turkey	[161]
*Uromyces aureus* Dietel & Holw.	*Allium trinulatum*, *A. validum*, *Allium* sp., and *Chlorogalum pomeridianum*	California and Washington (USA)	[78,186,187]
*Uromyces auriculae* (Magnus) A. Buchheim	*Primula auricula*	Austria and Germany	[160,283]
*Uromyces azorellae* Cooke	*Pozoa trifoliate* and *Schizeilematrifolio latum*	New Zealand	[213,284]
*Uromyces babianae* Doidge	*Babiana disticha*	Western Cape Province (South Africa)	[249,285]
*Uromyces baccarinii* Syd. & P. Syd.	*Wedelia* sp.	Eritrea	1937 [286,287]
*Uromyces badius* Syd.	*Haemanthus coccineus*, *H. pumilio*, *H. rotundifolius*, and *H. sanguineus*	South Africa	[138,167,288]
*Uromyces baeumlerianus* Bubák	*Melilotus* spp.	Austria, Bulgaria, China, Czech Republic, Hungary, India, Israel, Japan, Poland, Spain, Romania, Turkey, and Ukraine	[81,82,96,289,290]
*Uromyces bahiensis* Perd. Sánch.	*Loranthaceae*	Panama	[15]
*Uromyces basellae* Syd. & P. Syd.	*Basella rubra*	Malaysia	[15]
*Uromyces bauhiniae* Henn.	*Bauhinia bongardii*, *B. cuyabensis*, *B. hiemalis*, *B. longiflora*, *B. pauletia*, and *B. ungulata*	Brazil and México	[102,291,292]
*Uromyces bauhiniicola* Arthur	*Bauhinia chlorantha* and *B. pringlei*	México	[291]
*Uromyces beckeropsidis* E. Castell.	*Beckeropsis nubica*	Eritrea and Euthopia	[106,293,294]
*Uromyces beckmanniae* H.S. Jacks.	*Beckmannia eruciformis* and *B. syzigachne*	Oregon	[295]
*Uromyces behenis* (DC.) Unger	*Silene* spp.	Worldwide	[296,297]
*Uromyces belemensis* F.C. Albuq. & Figueiredo	*Ormosia nobilis*	Brazil	[219,298]
*Uromyces beloperones* G.F. Laundon	*Beloperone californica*, *B. purpusii*, and *Jacobinia mexicana*	Arizona, California, and México	[116,218,299,300]
*Uromyces bermudianus* Cummins	*Cyperus paniculatus*	Bermuda	[224,233]
*Uromyces bethelii* Arthur	*Silene verecunda*	California	[301]
*Uromyces beticola* (Bellynck) Boerema, Loer. & Hamers	*Beta vulgaris*	Bulgaria, Israel, and Turkey	[80,81,82,302]
*Uromyces bicolor* Ellis	*Allium* spp.	California, Idaho, Kansas, Massachusetts, Missouri, Montana, Maine, New York, Ohio, and Texas	[186,191,303,304,305]
*Uromyces bidenticola* (Henn.) Arthur	*Bidens* spp. and *Cosmos caudatus*	Africa, Asia, Central and South America, and the Southern United States	[96,121,306,307,308,309,310]
*Uromyces bidentis* Lagerh.	*Bidens* spp. and *Cosmos caudatus*	Central and South America, Southern United States, and West Indies	[118,119,152,184,264,311,312,313]
*Uromyces bisbyi* Savile	*Eriogonum parvifolium*	California	[314]
*Uromyces blainvilleae* Berk.	*Blainvillea* spp.	Brazil, Chinas, India, Nigeria, Sri Lanka, and Tanzania	[152,195,210,315,316,317]
*Uromyces blandus* Syd.	*Phragmites vulgaris*	China and Philippines	[318,319]
*Uromyces boissierae* Vienn. Bourg.	*Boissiera pumilio*	Iran	[320]
*Uromyces bolusii* Massee	*Aspalathus pachyloba*	South Africa	[138,321,322]
*Uromyces bomareae* Henn.	*Bomarea* sp.	Brazil	[167,261,323]
*Uromyces bonaerensis* Speg.	*Gomphrena elegans*	Buenos Aires	[324]
*Uromyces bonae-spei* Bubák	*Tritonia scillaris* and *Acidanthera pallida*	Southern Africa	[325]
*Uromyces bonaveriae* P. Syd.	*Bonaveria securidaca* and *Securigera securidaca*	Greece	[326,327]
*Uromyces borealis* Peck	*Hedysarum boreale*, *H. mackenzii*, and *Rumex arifolius*	Canada and Finland	[328,329]
*Uromyces bornmuelleri* Magnus	*Bongardia chrysogonum* and *Leontice armeniaca*	Cyprus, Iran, Iraq, Israel, and Turkey	[81,196,330]
*Uromyces borreriae* Henn.	*Borreria verticillata*	Rio de Janeiro (Brazil)	[331]
*Uromyces bosseri* Vienn. Bourg.	*Trochomeriopsis diversifolia*	Madagascar	[127,332]
*Uromyces bothriochloae-intermediae* Gorlenko	*Bothriochloa intermedia*	China	[108,109]
*Uromyces bouvardiae* Syd. & P. Syd.	*Bouvardia* spp.	Guatemala and México	[281,292,333]
*Uromyces bradburyae* H.S. Jacks. & Holw.	*Bradburya pubescens*, *B. virginiana*, *Centrosema pubescens*, and *C. virginianum*	Brazil	[101,102]
*Uromyces brandzae* Săvul.	*Lathyrus aureus*, *L. venetus*, and *Orobus vernus*	Romania and Ukraine	[111,126,228]
*Uromyces brasilianus* Speg.	*Senecio brasiliensis*	Buenos Aires	[334]
*Uromyces brasiliensis* Trotter	*Jacquemontia* spp.	Brazil, China, and México	[102,335,336]
*Uromyces bravensis* Cummins	*Sporobolus argutus* and *S. pyramidalis*	The Dominican Republic and Texas	[171,172,337]
*Uromyces bresadolae* Tranzschel	*Euphorbia* sp.	Itlay	[157]
*Uromyces briardii* Har.	*Euphorbia* sp.	Germany	[338]
*Uromyces brizae* Gäum., E. Müll. & Terrier	*Briza media*	France	[339]
*Uromyces brodiaeae* Ellis & Harkn	*Brodiaea* spp., *Hookera hyacinthina*, and *Triteleia ixioides*	California, Oregon, and Washington (USA)	[187,340]
*Uromyces bromicola* Arthur & Holw.	*Bromus coloratus* and *B. lithobius*	Chile	[199]
*Uromyces brominus* Gucevič	*Bromus riparius*	Ukraine and Russia	[106,111]
*Uromyces buforrestiae* Cummins	*Buforrestia imperforata*	Ghana	[341]
*Uromyces bugranae* A.L. Guyot	*Ononis columnae* and *O. striata*	France, Switzerland	[342]
*Uromyces bulbinicola* Doidge	*Bulbine bulbosa*	South Australia	[249,343]
*Uromyces bulbinus* Thüm.	*Bulbine* sp.	Australia and New Zealand	[344]
*Uromyces bunsteri* (Neger) H.S. Jacks. & Holw.	*Sisyrinchium cuspidatum*, *S. graminifolium*, and *Sisyrinchium* sp.	Chile	[166]
*Uromyces bupleuri* Magnus	*Bupleurum* spp.	China, Greece, Iran, Morocco, and Tibet	[91,190,220,345,346]
*Uromyces bylianus* Doidge	*Liliaceae*	KwaZulu-Natal	[249]
*Uromyces cacaliae* (DC.) Unger	*Adenostyles* spp. and *Cacalia* spp.	Austria, Germany, Japan, Poland, Romania, Russia, and Switzerland	[92,135,159,160]
*Uromyces cacciniae* Jørst.	*Caccinia strigosa* and *Mattiastrum* sp.	Afghanistan and Iran	[347,348]
*Uromyces cachrydis* Har.	*Cachrys pterochlaena*, *Hippomarathrum boissieri*, *H. crassilobum*, *H. siculum*, and *Prangos* sp.	Cyprus, Israel, Libya, Morocco, and Portugal	[81,349]
*Uromyces caladii* (Schwein.) Farl.	*Arisaema* spp., *Peltandra* spp., and *Muricauda dracontium*	Carolina, Georgia, Iowa, Minnesota, México, Missouri, Oklahoma, Texas, and West Virginia	[133,155,253,255]
*Uromyces calamagrostidis* Uljan.	*Calamagrostis arundinacea*	The former Soviet Union	[106]
*Uromyces callicarpae* (Petch) Fujik. ex S. Ito	*Callicarpa* spp.	China, Japan, and Taiwan	[94,96,97]
*Uromyces calopogonii* Cummins	*Calopogonium galactioides*	Guatemala	[171,172,224,350]
*Uromyces calotheus* Syd.	*Urginea*	Sierra Leone	[351]
*Uromyces calycotomes* Gäum. & Terrier	*Calycotome spinosa*	France	[220]
*Uromyces calystegiae* de Bary ex Fuckel	*Convolvulus arvensis*	America, Australia, Italy, and Germany	[352]
*Uromyces camphorosmae* (Castagne) Har.	*Camphorosma monspeliaca*	France	[353]
*Uromyces canavaliae* J.M. Yen	*Canavalia microcarpa*	China and Taiwan	[97,108,109]
*Uromyces capensis* Doidge	*Oenothera biennis*	Western Cape Province	[138]
*Uromyces capitatus* Syd. & P. Syd.	*Desmodium yunnanensis*	China, India, and Pakistan	[96,156,354,355,356,357]
*Uromyces caraganae* (Thüm.) Magnus	*Caragana arborescens*	Portugal	[358,359]
*Uromyces caraganicola* Henn.	*Caragana chamlagu*	Asia, Africa, and Europe	[81,225,236,360,361,362,363]
*Uromyces caricis-brunneae* Morim.	*Carex brunnea*	Japan	[94]
*Uromyces caricis-dolichocarpae* Azbukina	*Carex dolichocarpa*	Sakhalin (Russia)	[364]
*Uromyces caricis-rafflesianae* Mayor	*Carex rafflesiana*	Philippines	[365]
*Uromyces caricis-schmidtii* Tomilin	*Carex schmidtii*	Khabarovsk	[297,366]
*Uromyces caricis-sempervirentis* E. Fisch.	*Carex sempervirens*, *C. sempervirens*, *C. stenophylla*, *Phyteuma betonicifolium*, and *P. orbiculare*	Austria, Europe, Germany, Poland, Romania, Switzerland, and Turkey	[43,80,160]
*Uromyces carneus* (Nees) Har.	*Astragalus alpinus* and *Oxytropis campestris*	Finland and Wyoming	[329,367,368]
*Uromyces carpathicus* Namysł.	*Geranium phaeum*	Poland	[369,370]
*Uromyces carthagenensis* Speg.	*Manihot* spp.	Argentina, Brazil, and Uruguay	[371,372]
*Uromyces cassiae-mimosoidis* (Doidge) Doidge	*Cassia mimosoides* and *Chamaecrista mimosoides*	South Africa	[138,171,172,285]
*Uromyces castaneus* P. Syd. & Syd.	*Desmodium incanum*, *D. purpureum*, and *Desmodium* sp.	Argentina and Brazil	[171,269]
*Uromyces cayaponiae* Henn.	*Cayaponia* sp.	Santa Catarina	[127]
*Uromyces cearensis* Berndt & F.O. Freire	*Ipomoea* sp.	Brazil	[373]
*Uromyces cedrelae* (Henn.) Henn.	*Toona serrata*	Indonesia	[177,374]
*Uromyces celosiae* Dietel & Holw.	*Celosia latifolia*	Argentina, Brazil, Costa Rica, Cuba, Jamaica, and México	[167,263,269,308,375,376]
*Uromyces celtidis* Dietel	*Celtis* sp.	Brazil	[102,375]
*Uromyces cenisiae* A.L. Guyot	*Ononis cenisia*	France	[342]
*Uromyces ceratocarpi* Syd. & P. Syd.	*Ceratocarpus arenarius*	China and Russia	[91,144]
*Uromyces cestricola* Speg.	*Cestrum pubeens* and *C. strigilatum*	Argentina and Bolivia	[334,377]
*Uromyces chaetobromi* Gjaerum	*Chaetobromus dregeanus* and *C. schraderi*	South Africa	[244,378]
*Uromyces chaetolimonis* Korbonsk.	*Chaetolimon sogdianum*	Tajikistan	[379]
*Uromyces charmelii* Liou	*Phyteuma charmelii*	France	[380]
*Uromyces chenopodii* J. Schröt	*Chenopodium* sp., *Salsola* spp., and *Suaeda* spp.	Around the world	[381]
*Uromyces chenopodii-fruticosi* (DC.) M. Abbasi & Aime	*Suaeda maritima*	England and Germany	[47]
*Uromyces chesneyae* Tranzschel & Erem.	*Chesneya astragalina*	Central Asia and Russia	[382,383]
*Uromyces chevalieri* A.L. Guyot	*Drimiopsis* sp.	Chari and Tropical Africa	[384,385]
*Uromyces chilensis* Dietel & Neger	*Lathyrus magellanicus* and *L. multiceps*	Chile	[162,386]
*Uromyces chiovendae* Bacc.	*Cissus* sp.	Somalia	[287]
*Uromyces chloridis* Doidge	*Chloris myriostachya*, *C. pilosa*, and *C. virgate*	Uganda and South Africa	[138,249]
*Uromyces chlorogali* Dietel & Holw.	*Chlorogalum pomeridianum*	California and Washington (USA)	[186,187,387]
*Uromyces chorizanthis* Ellis & Harkn.	*Chorizanthe pungens*	California	[340,388]
*Uromyces christensenii* J. Anikster & I. Wahl	*Muscari parviflorum* and *Hordeum bulbosum*	Israel	[389,390]
*Uromyces chubutensis* Speg.	*Poa chubutensis*	Chubut	[391]
*Uromyces ciceris-arietini* (Grognot) Jacz. & G. Boyer	*Cicer arietinum*, *Trigonella polycerata*, and *Vicia* spp.	Brazil, India, Kenya, Libya, México, Morocco, and Pakistan	[81,221,357,392]
*Uromyces ciceris-soongaricae* S. Ahmad	*Cicer songaricum*	Pakistan	[393]
*Uromyces circinalis* Kalchbr. & Cooke	*Scilla prasina*	South Africa	[138]
*Uromyces circumscriptus* Neger	*Loranthus* spp., *Struthanthus complexus*, and *Phrygilanthus* spp.	Argentina, Brazil, and Chile	[394]
*Uromyces cisneroanus* Speg.	*Excoecaria biglandulosa* var. *serrata* and *Sapium* spp.	Argentina, Brazil, Paraguay, and Venezuela	[100,101,103,269,324]
*Uromyces cladomanes* Traverso	*Vitis* sp.	Somalia	[287]
*Uromyces cladrastidis* Kusano	*Cladrastis shikokiana*	Japan	[92]
*Uromyces clarus* H.S. Jacks. & Holw.	*Iresine angustifolia*, *I. celosia* and *Iresine* sp.	Bolivia, Brazil, Cuba, Venezuela, and West Indies	[167,263,376]
*Uromyces clavatus* Dietel	*Lathyrus* spp. and *Vicia* spp.	Argentina, Chile, and Uruguay	[143,240,395]
*Uromyces claytoniae* Cooke & Peck	*Claytonia caroliniana*	Canada and New York	[104,185,396]
*Uromyces clignyi* Pat. & Har.	*Andropogon* spp., *Bothriochloa* spp., *Capillipedium* sp., *Cymbopogon* sp., *Dichanthium* spp., *Eremopogon* sp., *Exotheca* sp., *Hemarthria* spp., *Heteropogon* sp., *Monocymbium* sp., *Schizachyrium* sp., *Sorghum* sp., and *Themeda* spp.	Uganda, Australia, Cameroon, China, Ethiopia, Ghana, Guatemala, India, Japan, Kenya, México, Nepal, Nigeria, Pakistan, South Africa, Tanzania, Uganda, and Zambia	[110,152,171,172,243,244,356,397]
*Uromyces clignyioides* Gjaerum	*Monocymbium ceresiiforme*	Zimbabwe	[244]
*Uromyces clitoriae* Arthur	*Clitoria mexicana*	México	[172,398]
*Uromyces clivalis* Mitter	*Argyrolobium flaccidum*	India	[357,399]
*Uromyces clutiae* Kalchbr. & Cooke	*Clutia* sp.	Kenya	[400]
*Uromyces cnidoscoli* Henn.	*Cnidoscolus vitifolius* and *Jatropha* sp.	Argentina and Brazil	[103,282,291]
*Uromyces cobresiae* Korbonsk.	*Carex* sp.	Uzbekistan	[146]
*Uromyces colchici* Massee	*Colchicum spectabile* and *C. bavaricum*	Great Britain and Turkey	[133,401,402]
*Uromyces collinus* J.F. Hennen & Cummins	*Bauhinia* sp.	México	[133,292,403]
*Uromyces cologaniae* Arthur	*Cologania* spp. and *Teramnus uncinatus*	Costa Rica, Guatemala, México, Puerto Rico, and Venezuela	[259,281,306,310,398]
*Uromyces coloradensis* Ellis & Everh.	*Astragalus* sp. and *Vicia* spp.	Arizona, California, Canada, Colorado, Iowa, Utah, Washington, and Wisconsin (USA)	[225,404]
*Uromyces columbianus* Mayor	*Melanthera* spp.	Florida, West Indies, and Central and South America	[76,77,120,122,226,306]
*Uromyces coluteae* Arthur	*Colutea arborescens*	Austria	[299]
*Uromyces combreti* Thaung	*Combretum*	Myanmar	[405]
*Uromyces comedens* P. Syd. & Syd.	*Jasminum pubescens*	India and Dominican Republic	[118,119,144,224,406]
*Uromyces commelinae* Cooke	*Amischotolype* sp., *Aneilema* spp., *Callisia* spp., *Commelina* spp., *Cyanotis* spp., *Cyanotis* spp., *Pollia* spp., *Tradescantia* spp., *Tripogandra* sp., and *Zebrina* sp.	Worldwide	[222,407]
*Uromyces compactus* Peck	*Aster spinosus, Boltonia diffusa*, and *Baccharis* sp.	Arizona, México, and Texas	[116,337,408,409]
*Uromyces comptus* P. Syd. & Syd.	*Ipomoea bipinnatipartita*	Namibia, South Africa, and Tanzania	[126,138,165,384]
*Uromyces congoensis* Syd. & P. Syd.	*Bauhinia* sp.	The Democratic Republic of the Congo	[410]
*Uromyces conicus* Jørst.	*Cleome* sp.	Bolivia	[411]
*Uromyces coordinatus* Arthur	*Euphorbia* spp. and *Tithymalus palmeri*	California and Utah	[103,114,404,412]
*Uromyces corallocarpi* W.T. Dale	*Corallocarpus emetocatharticus* and *Doyerea emetocathartica*	Trinidad and Tobago, México, and West Indies	[127,211,212,308]
*Uromyces cordiae* Henn.	*Cordia* sp.	Rio de Janeiro	[413]
*Uromyces coronatus* Yoshin.	*Helictotrichon milanjianum*, *Oryza sativa*, *Zizania* spp., and *Zizaniopsis* sp.	China, Florida, Hong Kong, Japan, Korea, Taiwan, Thailand, and Uganda	[39,94,95,96,97,262,414]
*Uromyces coronillae* Vienn.-Bourg.	*Coronilla rostrate* and *C. varia*	Israel and Switzerland	[81,220]
*Uromyces correntinus* J.C. Lindq.	*Rhynchospora tenuis*	Argentina	[415]
*Uromyces corrugatus* Speg.	*Vicia patagonica*	Argentina and Brazil	[126,180]
*Uromyces costaricensis* Syd.	*Lasiacis* sp. and *Panicum altissimum*	Brazil, Colombia, Costa Rica, Florida, México, Puerto Rico, and Venezuela	[106,229,294]
*Uromyces costesianus* Speg.	*Sphaeralcea velutina*	Chile	[386,416]
*Uromyces crassipes* Dietel & Neger	*Rumex* spp.	Argentina, Chile, and Peru	[269,281,376,417]
*Uromyces crassivertex* Dietel	*Lychnis miqueliana* and *Lychnis* sp.	China and Japan	[92,94,418]
*Uromyces crepidis-fraasii* Kaps. Gotsi	*Crepis fraasii*	Greece	[419]
*Uromyces cretensis* Petr.	*Coronilla parviflora* and *C. rostrata*	Greece	[420,421]
*Uromyces cristatulus* Tranzschel	*Euphorbia* sp.	Australia, Germany, Hungary, and Russia	[157]
*Uromyces cristatus* J. Schröt. & Niessl	*Dianthus caryophyllus*, *Lychnis viscaria*, *Viscaria alpina*, and *V. vulgaris*	Portugal, Czechoslovakia, Denmark, Finland, Greece, Norway, Poland, and Sweden	[105,128,129,135,158]
*Uromyces cristulatus* Tranzschel	*Euphorbia barrelieri*, *E. petrophila*, *E. salicifolia*, and *E. seguieriana*	Bulgaria and Ukraine	[82,111,157,422]
*Uromyces croci* Pass.	*Crocus* spp.	Bulgaria, Israel, Romania, Russia, and Ukraine	[81,82,165,422]
*Uromyces crotalariae-nitens* Salazar-Yepes & Buriticá	*Crotalaria nitens*	Colombia	[423]
*Uromyces cruchetii* Mayor	*Borreria tenella*	Colombia	[226]
*Uromyces cruckshanksiae* Cummins & Bonar	*Cruckshanksia bustillosii*, *C. palma*, *Oreopolus glacialis*, and *O. palmae*	Argentina and Chile	[233,240,415]
*Uromyces cucubali* Hirats. & Hashioka	*Cucubalus baccifer*	China, Japan, and Taiwan	[89,96,97]
*Uromyces cucullatus* Syd. & P. Syd.	*Baltimora recta*, *Perymenium* spp., *Wedelia* spp., and *Zexmenia* spp.	Cuba, Costa Rica Guatemala, México, and Panama	[124,281,398,424,425]
*Uromyces cucumivorus* Berndt	*Cucumis melo* var. *flexuosus*	Iraq	[127]
*Uromyces cuenodii* Maire	*Silene eriocalycina*	Iraq	[196]
*Uromyces cuspidatus* G. Winter	*Festuca* spp., *Melica laxiflora*, *Muhlenbergia* spp., and *Poa chubutensis*	Argentina, Bolivia, and Chile	[106,199,240]
*Uromyces cyanotidis* Cummins	*Cyanotis capitata*	Papua New Guinea	[233,426]
*Uromyces cyathulae* Henn.	*Cyathula globulifera*	Eritrea	[287,427]
*Uromyces cyperi* Henn.	*Cyperus* sp.	Eritrea	[287,427]
*Uromyces cypericola* Gjaerum	*Cyperus albostriatus* and *C. cyperoides*	Kenya, South Africa, and Uganda	[428,429]
*Uromyces cyprius* Vienn.-Bourg.	*Rumex cyprius*	Iran	[430]
*Uromyces cystopiformis* Lagerh.	*Centropogon* sp. and *Siphocampylus* sp.	Costa Rica, Ecuador	[165,310,431]
*Uromyces cytisi* J. Schröt.	*Caragana arborescens*, *Cytisus polytrichus*, *Genista aucheri*, *G. tinctoria*, and *Laburnum anagyroides*	Russia, Ukraine, and Turkey	[80,111,432]
*Uromyces dactylidis* G.H. Otth	*Agrostis* spp., *Alopecurus* spp., *Anemone* sp., *Avenula* sp., *Dactylis* spp., *Festuca* spp., *Poa* spp., *Ranunculus* spp., and *Trisetum* sp.	Arizona, Armenia, Australia, Bulgaria, Pakistan, Canada, China, Checz Republic, Czechoslovakia, Denmark, Finland, Germany, Hungary, Japan, New Zealand, Norway, Oklahoma, Pennsylvania, Romania, Spain, Sweden, Switzerland, Ukraine, Virginia, Vermont, and other temperateregions of the world	[43,80,82,105,113,368,409,433,434,435,436,437,438,439,440,441]
*Uromyces dactyloctenii* Wakef. & Hansf.	*Dactyloctenium aegyptium*	China, Japan, Kenya, Namibia, Samoa, Tanzania, Tonga, and Uganda	[140,168,274,442,443]
*Uromyces dactylocteniicola* (Speg.) J.C. Lindq.	*Dactyloctenium aegyptium*	Central Africa, the Philippines, and South America	[106]
*Uromyces danthoniae* McAlpine	*Danthonia* spp. and *Rytidosperma* spp.	Australia and New Zealand	[245,280,313]
*Uromyces decoratus* Syd. & P. Syd.	*Crotalaria* spp.	China, Costa Rica, Guinea, Ghana, India, Japan, Madagascar, Myanmar, Nigeria, Pakistan, Sri Lanka, Taiwan, Thailand, and Venezuela	[85,86,94,96,97,152,171,172,281,290,312,356,357,444,445]
*Uromyces deeringiae* Syd. & P. Syd.	*Cladostachys polysperma* and *Deeringia* spp.	China, Japan, India, Indonesia, Philippines, and Taiwan	[92,167,232,309,446,447]
*Uromyces delagoënsis* Bubák	*Lapeirousia delagoensis*	Mozambique	[249]
*Uromyces dendroseridis* Keissl.	*Dendroseris micrantha*	Chile	[240]
*Uromyces densus* Arthur	*Bidens pilosa*	Puerto Rico	[76,77]
*Uromyces desmodii* Cooke	*Desmodium canescens*	South Carolina and Brazil	[448]
*Uromyces desmodiicola* Jørst.	*Desmodium albiflorum*	Brazil	[102,171,449]
*Uromyces desmodii-leiocarpi* Henn.	*Desmodium leiocarpum*	Brazil	[171,450]
*Uromyces devoluensis* Gäum.	*Senecio doronicum*	France	[451]
*Uromyces dianthi* (Pers.) Niessl	*Arenaria leptoclados*, *Bufonia* spp., *Cerastium brachypetalum*, *Dianthus* spp., *Euphorbia* spp., *Gypsophila* spp., *Petrorhagia* spp., *Silene* sp., *Vaccaria* sp., and *Vicia* sp.	Australia, Bermuda, Bolivia, Bulgaria, Brazil, California, Chile, Cuba, Germany, Japan, Kenya, Madagascar, New York, Poland, Romania, Spain, Thailand, Texas, Turkey, Ukraine, and Washington (USA)	[80,82,94,105,111,135,140,452]
*Uromyces dianthi-caryophylli* Monchot	*Dianthus* sp.	France	[453]
*Uromyces dichromenae* W.T. Dale	*Dichromena radicans*	Jamaica, Trinidad and Tobago, and West Indies	[264,308]
*Uromyces dictyospermae* Ellis & Everh. ex Tranzschel	*Euphorbia* spp., *Jatropha* spp., and *Tithymalus* sp.	California, Florida, Minnesota, Montana, Oklahoma, and Washington (USA)	[103,157,187,254,262,278,388,454,455,456]
*Uromyces didymae* Gapon.	*Veronica polita*	Uzbekistan	[457]
*Uromyces dieramatis* Doidge	*Dierama* spp.	South Africa	[123,138,139,273,458]
*Uromyces dietelianus* Pazschke	*Bauhinia* spp.	Argentina, Brazil, and Uruguay	[100,101,269,297]
*Uromyces digitariae-adscendentis* Y.C. Wang	*Digitaria* spp.	China and Taiwan	[96,97]
*Uromyces dilucidus* Cummins	*Sisyrinchium striatum*	Argentina	[415,459]
*Uromyces diniensis* A.L. Guyot	*Ononis fruticosa*	France	[460]
*Uromyces dinteri* Mennicken, W. Maier & Oberw.	*Tetraena* spp.	Namibia and Egypt	[385]
*Uromyces dipcadi* Gjaerum	*Dipcadi viride*	Kenya	[461,462]
*Uromyces discariae* G. Cunn.	*Discaria toumatou*	New Zealand	[284,313,463]
*Uromyces dispersus* Hirats. f.	*Apios fortunei*	Japan	[222]
*Uromyces dobremezii* Durrieu	*Euphorbia stracheyi*	Nepal	[464]
*Uromyces doebbeleri* Berndt	*Hypericum irazuense*	Costa Rica	[310]
*Uromyces dolichi* Cooke	*Dolichos axillaris*	Brazil, China, Madagascar, Puerto Rico, Zimbabwe, and Uganda	[108,109,125,171,172,250,465]
*Uromyces dolicholi* Arthur	*Cajanus* spp., *Dolicholus* spp. and *Rhyncosia* spp.	Canada, China, Colombia, Costa Rica, Florida, India, Japan, Jamaica, Kenya, Panama, Puerto Rico, South Africa, Taiwan, Texas, Venezuela, and West Indies	[96,97,138,227,310,357,466,467,468,469]
*Uromyces dolichosporus* Dietel & Holw.	*Tournefortia* spp.	Argentina, Brazil, Bolivia, Cuba, Ecuador, Jamaica, México, West Indies, and Venezuela	[100,101,263,269,308,375,456]
*Uromyces doricus* Maire	*Silene* spp.	Bulgaria and Greece	[82,308,327,470]
*Uromyces dorystaechadis* Gjaerum & Bahç.	*Dorystaechas hastata*	Turkey	[471]
*Uromyces drimiopsidis* Doidge	*Drimiopsis maculata*	South Africa	[138]
*Uromyces dubiosus* Henn.	*Lantana* sp.	Goiás	[291]
*Uromyces ducellieri* Maire	*Anabasis aphylla*	China	[91,96,472]
*Uromyces dusenii* Dietel & Neger	*Gilliesia graminea*, *G. monophylla, Miersia chilensis*, and *Ornithogalum biflorum*	Chile	[166,240,417]
*Uromyces echinodes* Kunze ex Henn.	*Asclepiadaceae*	Suriname	[473]
*Uromyces ecklonii* Bubák	*Freesia refracta*	Kenya and South Africa	[138,140]
*Uromyces eclipsis* Berndt	*Zygophyllum morgsana*	South Africa	[234]
*Uromyces edwardsiae* G. Cunn.	*Edwardsia* spp. and *Sophora* spp.	New Zealand	[284,463,474]
*Uromyces ehrhartae* McAlpine	*Ehrharta stipoides*	Australia and New Zealand	[106,280,313,474]
*Uromyces ehrhartae-giganteae* Doidge	*Ehrharta* spp.	South Africa	[138,249,378,429]
*Uromyces elegans* (Berk. & M.A. Curtis) Lagerh.	*Trifolium* spp.	Florida, Kenya, Mississippi, Oklahoma, South Carolina, Texas, and Uganda	[168,261,455,475]
*Uromyces eleocharidis* Arthur	*Eleocharis* spp.	Iowa, Kansas, North and South Dakota, and Washington (USA)	[155,278,301,466,476]
*Uromyces ellipticus* Dietel & Neger	*Glycyrrhiza astragalina*	Chile	[240,477]
*Uromyces ellisianus* Henn.	*Euphorbia marginata*	Minnesota	[277]
*Uromyces emmeorhizae* Syd.	*Emmeorhiza umbellata*	Venezuela	[478]
*Uromyces epicampis* Dietel & Holw.	*Epicampes macroura*, *Melica laxiflora*, and *Muhlenbergia* spp.	Arizona, California, Chile, Ecuador, Guatemala, México, and Texas	[106,116,199,240,281,337,479]
*Uromyces eragrostidicola* Gjaerum	*Eragrostis rigidior* and *Eragrostis* sp.	Ethiopia, Kenya, Tanzania, and Zimbabwe	[244]
*Uromyces eragrostidis* Tracy	*Anthericum torreyi*, *Cypholepis yemenica*, *Desmostachya bipinnata*, *Eragrostis* spp., and *Tripogon chinensis*	Arizona, Argentina, Australia, Botswana, Brazil, China, Georgia, Ghana, India, Israel, Kenya, Malawi, Malaysia, Mississippi, México, Nebraska, Oklahoma, Pakistan, Puerto Rico, South Africa, Texas, West Indies, and Venezuela	[90,110,116,141,171,172,199,208,243,244,250,480]
*Uromyces eriochloae* (Syd. & P. Syd.) Syd., P. Syd. & E.J. Butler	*Eriochloa* spp.	China, Indonesia, Japan, Madagascar, and Philippines	[85,90,232,309,465]
*Uromyces eriogoni* Ellis & Harkn.	*Eriogonum virgatum*	California	[340,388]
*Uromyces eriospermi* Kalchbr. & Cooke	*Eriospermum* spp.	South Africa, Tanzania, and Zimbabwe	[137,138,250,274]
*Uromyces ermelensis* Doidge	*Indigofera* sp.	South Africa	[138]
*Uromyces ervi* (Wallr.) Westend.	*Ervum* spp., *Lens* spp., *Pisum sativum*, and *Vicia* spp.	Algeria, Austria, Belgium, Bulgaria, China, Czech Republic, Denmark, Finland, France, Germany, Hungary, Italy, Japan, Morocco, Netherlands, Pakistan, Poland, Portugal, Romania, Russia, Spain, Switzerland, Sweden, Taiwan, and Ukraine	[82,96,128,220,393,481,482]
*Uromyces erythrinae* Lagerh.	*Erythrina* sp.	Ecuador	[165]
*Uromyces erythronii* (DC.) Pass.	*Erythronium* sp., *Lilium* spp., and *Tulipa* spp.	Bulgaria, China, Germany, Greece, Japan, Japan, Korea, and Spain	[82,92,96,158,179,483]
*Uromyces eugenei-mayorii* M. Morelet	*Ulex europaeus*	Italy	[451]
*Uromyces eugentianae* Cummins	*Gentiana* spp. and *Halenia guatemalensis*	Canada, Iowa, Norway, and Washington (USA)	[129,187,252,484,485,486]
*Uromyces eulophiae* Gjaerum	*Eulophia paivaena* subsp. *borealis*	India, Rwanda, and Uganda	[318,487,488]
*Uromyces euphaeus* Syd.	*Hypoxis glabella*	Australia	[489]
*Uromyces euphlebius* Syd. & P. Syd.	*Phoradendron calyculatus*, and *Phoradendron* sp.	México	[15,490]
*Uromyces euphorbiae* Cooke & Peck	*Acalypha* communis, Chamaesyce sp., *Euphorbia* spp., and *Poinsettia heterophylla*	Afghanistan, Argentina, Azirona, Brazil, California, Canada, China, Cuba, Columbia, Costa Rica, Cyprus, India, Israel, Jamaica, Japan, Mississippi, Mauritius, New York, Pakistan, Panama, South Africa, Texas, Uganda, Venezuela, West Indies, and Zambia	[96,102,203,209,227,255,263,269,300,308,310,318,348,469,475,488,491,492]
*Uromyces euphorbiae-connatae* Speschnew	*Euphorbia* sp.	Russia	[493]
*Uromyces euphorbiae-javanicae* E. Fisch.	*Euphorbia javanica*	Indonesia	[309]
*Uromyces euphorbiae-lunulatae* Liou & Y.C. Wang	*Euphorbia esula*, *E. kansui*, and *E. lunulata*	China	[96,108,109]
*Uromyces euphorbiae-nicaeensis* Unamuno	*Euphorbia nicaeensis*	Spain	[494]
*Uromyces euphorbiae-polytimeticae* Zenkova	*Euphorbia polytimetica*	Tajikistan	[495]
*Uromyces euphorbiicola* (Berk. & M.A. Curtis) Tranzschel	*Euphorbia* spp.	Brazil, Colombia, Madagascar, South Africa, and Zimbabwe	[102,138,157,250,465]
*Uromyces eurotiae* Tranzschel	*Ceratoides latens*, *Krascheninnikovia ceratoides*, and *K. latens*	China, Turkey, and Uzbekistan	[80,91,146,161]
*Uromyces euryopsidicola* A.R. Wood & M. Scholler	*Euryops empetrifolius* and *E. tenuissimus*	South Africa, North and Western Cape Province	[496]
*Uromyces evastigatus* Cummins	*Phthirusa pyrifolia*	El Salvador	[15]
*Uromyces fallens* (Arthur) Barthol.	*Trifolium pratense*	Iran	[497]
*Uromyces fatouae* Henn.	*Fatoua pilosa*	Japan	[498]
*Uromyces fedtschenkoi* Faizieva	*Rumex fedtschenkoi*	Uzbekistan	[457]
*Uromyces ferganensis* Tranzschel & Erem.	*Stipa caucasica*, *S. holosericea*, and *S. lessingiana*	Iran, Siberia, and Russia	[106,382,499]
*Uromyces ferrariae* Doidge	*Ferraria* sp.	South Africa and Zimbabwe	[138,250]
*Uromyces ferulae* Juel	*Ferula* spp. and *Heracleum cachemiricum*	Australia, Iran, Israel, Pakistan, and Morocco	[81,500]
*Uromyces ferulaginis* Lindr.	*Feruladinis silvaticae*	Poland	[501]
*Uromyces festucae-nigricantis* Gonz. Frag.	*Festuca nigricans*	Morocco and Spain	[279,502]
*Uromyces ficariae* (Schumach.) Lév.	*Ficaria* spp. and *Ranunculus* spp.	Bulgaria, Czech Republic, Czechoslovakia, Denmark, Finland, Germany, Iran, Norway, Poland, Russia, Sweden, Turkey, and Ukraine	[80,82,83,105,111,128,129,132,432,438,503]
*Uromyces fiebrigii* Henn. & Vestergr.	*Bauhinia* sp.	Paraguay	[216]
*Uromyces fiorianus* Sacc.	*Peucedanum fraxinifolium* and *Peucedanum* sp.	South Africa	[138,504]
*Uromyces fischerianus* Mayor	*Ranunculus glacialis*	Switzerland	[119]
*Uromyces fischeri-eduardi* Magnus	*Vicia* spp.	Bulgaria, Mongolia, Poland, Romania, and Turkey	[80,82,135,505]
*Uromyces flavicomae* Liou	*Euphorbia flavicoma*	France	[506]
*Uromyces flemmingiae* Henn.	*Flemmingia* sp.	Uganda	[507]
*Uromyces fleuryae* J.M. Yen	*Fleurya podocarpa*	Gabon	[508]
*Uromyces floralis* Vestergr.	*Bauhinia hiemalis*, *B. cuyabensis*, *B. holophylla*, *B. rufa*, and *Bauhinia* sp.	Brazil	[102,216]
*Uromyces floscopae* Syd. & P. Syd.	*Floscopaperuviana* and *Floscopa* sp.	Brazil	[102,509]
*Uromyces fontii* Gonz. Frag.	*Peplis acutangula*	Morocco	[510]
*Uromyces formosus* Syd. & P. Syd.	*Dianthus libanotis*	Israel and Iran	[81]
*Uromyces foveolatus* Juel	*Bauhinia hirsuta*, *B. mirandina*, and *Bauhinia* sp.	Brazil	[100,101,102,511]
*Uromyces fragilipes* Tranzschel	*Agropyron squarrosum*, *Agrostis* spp., *Deschampsia* spp., *Eremopyrum bonaepartis*, *Festuca* spp., *Hordeum* spp., and *Scribneria bolanderi*	California, Iran, Pakistan, and Russia	[106,161,347,412,512,513]
*Uromyces fraserae* Arthur & Ricker	*Frasera speciosa*	Wyoming	[514]
*Uromyces fremonti* Syd. & P. Syd.	*Oenothera fremontii*	Kansas	[79]
*Uromyces fulgens* Bubák	*Chamaecytisus* spp., *Cytisus* spp., and *Lembotropis nigricans*	Bulgaria, Czech Republic, France, Greece, Hungary, Itlay, Poland, Romania, Russia, Ukraine, and Yugoslavia	[82,220,421]
*Uromyces fuscatus* Arthur	*Polygonum alpinum*	Idaho and Utah	[281]
*Uromyces fusisporus* Cooke & Massee	*Acacia neriifolia*, *A. salicina*, and *Acacia* sp.	Australia	[177,515]
*Uromyces gaeumannii* Mayor & Vienn. Bourg.	*Hippocrepis* spp.	France, Greece, Libya, Morocco, Palestine, Portugal, Spain, Tunisia, and Yugoslavia	[220,516]
*Uromyces gageae* Beck	*Gagea* spp., *Lloydia triflora*, and *Ornithogalum umbellatum*	Austria, Bulgaria, Czech Republic, China, Denmark, Finland, Germany, Japan, Norway, Poland, Romania, Russia, Turkey, Sweden, and Ukraine	[54,80,82,128,135,222,228,440]
*Uromyces galactiae* Rezende & Dianese	*Galactia pedunculata*	Brazil	[517]
*Uromyces galegae* Sacc.	*Astragalus glycyphylloides* and *Galega* spp.	Czech Republic, France, Greece, Hungary, Italy, Poland, Russia, Turkey, and Yugoslavia	[80,82,220,421]
*Uromyces galegicola* Woron.	*Galega orientalis*	Armenia, Romania, and Turkey	[80,228,518,519]
*Uromyces galii* Dietel	*Galium aparine* and *G. spurium*	Japan	[222,414]
*Uromyces galii-californici* Linder	*Galium californicum* and *Galium* sp.	California	[520]
*Uromyces galphimiae* Dietel & Holw.	*Galphimia glauca* and *G. humboldtiana*	México	[456,479]
*Uromyces garanbiensis* (Hirats. f. & Hashioka) Sawada	*Ehretia dicksonii*	Taiwan	[521]
*Uromyces gaubae* Petr.	*Caltha introloba*	Australia	[346,522]
*Uromyces gausseni* Mayor & Vienn.-Bourg.	*Dorycnopsis gerardii*	France	[516]
*Uromyces geissorhizae* Henn.	*Geissorhiza* sp.	Western Cape Province	[523]
*Uromyces gemmatus* Berk. & M.A. Curtis	*Convolvulus parviflorus* and *Convolvulus* sp.	Brazil, China, Cuba, Ecuador, India, Japan, Jamaica, México, Taiwan, West Indies, and Venezuela	[94,96,97,100,101,122,264,306,319,524,525]
*Uromyces genistae* Fuckel	*Cytisus capitatus*, *Genista tinctoria, Genista* sp., and *Tithymalus cyparissias*	Czech Republic, Morocco, and Turkey	[80,83,220,525]
*Uromyces geranii* (DC.) G.H. Otth & Wartm.	*Erodium* spp. and *Geranium* spp.	Australia, Bulgaria, China, Chile, Czech Republic, Denmark, Finland, France, Germany, India, Japan, Kenya, Korea, Norway, Pakistan, Poland, Portugal, Romania, Russia, Spain, Sweden, Turkey, Ukraine, and Zimbabwe	[80,82,87,88,94,96,128,179,208,436,526]
*Uromyces geraniicola* Speg.	*Geranium patagonicum*	Chile	[240,258]
*Uromyces ghaznicus* Petr.	*Limonium* sp.	Afghanistan	[527]
*Uromyces giganteus* Speg.	*Kalidium foliatum* and *Suaeda* spp.	Australia, Arizona, China, California, Idaho, Texas, and Ukraine	[47,96,300,337,421]
*Uromyces gigantiformis* Salazar-Yepes & Buriticá	*Bidens* sp.	Colombia	[423]
*Uromyces gilgitae* S. Ahmad	*Sophora alopecuorides*	Pakistan	[528]
*Uromyces gladioli* Henn.	*Babiana* spp., *Geissorhiza* spp., *Gladiolus* spp., *Moraea ramose*, and *Romulea* spp.	Cuba, Kenya, Malawi, Nigeria, South Africa, and Uganda	[138,140,168,273,291,458,529,530]
*Uromyces globosus* Dietel & Holw.	*Sapium* spp.	México	[103,456,479]
*Uromyces glyceriae* Arthur	*Glyceria* spp.	Illinois, Indiana, New Jersey, Rhode Island, and Wisconsin (USA)	[39,191,299]
*Uromyces glycyrrhizae* (Rabenh.) Magnus	*Glycyrrhiza* spp.	Armenia, Algeria, Azerbaijan, Bulgaria, Caucasus, Czech Republic, China, Greece, Iraq, Israel, Itlay, Japan, Kazakhstan, Pakistan, Palestine, Portugal, Romania, Spain, Turkey, and Uzbekistan	[39,78,80,81,82,91,96,116,146,196,278,317,327,356,393,409,531,532,533,534,535,536]
*Uromyces gnaphalii* Ellis & Everh.	*Gnaphalium* sp.	Colorado	[388]
*Uromyces gouaniae* F. Kern	*Gouania domingensis*, *G. lupuloides*, *Ledenbergia macrantha*, and *Ledenbergia* sp.	Guatemala and México	[281,486,537]
*Uromyces goyazensis* Henn.	*Bauhinia* sp.	Brazil	[291,538]
*Uromyces graminis* (Niessl) Dietel	*Caucalis platycarpos*, *Ferula communis*, *Melica* spp., and *Thapsia garganica*	Afghanistan, Armenia, Bulgaria, Greece, Italy, Morocco, Romania, Spain, Portugal, and Ukraine	[82,111,158,228,348,421,519,534,539]
*Uromyces grandiotii* Gäum.	*Ancrumia cuspidata*	Chile	[540]
*Uromyces greenstockii* Doidge	*Ipomoea greenstockii*	South Africa	[138]
*Uromyces guatemalensis* Vestergr.	*Bauhinia inermis*, *B. ungulata*, and *Bauhinia* sp.	Costa Rica, El Salvador, Guatemala, and Venezuela	[216,281,444,541]
*Uromyces guayacuru* Speg.	*Statice brasiliensis*	Buenos Aires	[542]
*Uromyces gueldenstaedtiae* Liou & Y.C. Wang	*Populus* sp.	China	[543]
*Uromyces guerkeanus* Henn.	*Lotus* spp. and *Tetragonolobus biflorus*	Belarus, Egypt, France, Greece, Italy, Iraq, Malta, Morocco, Palestine, Portugal, Spain, Syria, Tunisia, and Yugoslavia	[196,220,422,426,544]
*Uromyces guraniae* Mayor	*Gurania* sp.	Brazil and Colombia	[127,212,226]
*Uromyces gypsophilae* Cooke	*Gypsophila* sp.	Asia, Europe, Turkey, Iran, and Iraq	[80,82,165,196,317,346,545,546,547]
*Uromyces habrochloae* Gjaerum	*Habrochloa bullockii*	Malawi	[244]
*Uromyces hainanicus* J.Y. Zhuang & S.X. Wei	*Ipomoea sumatrana*	China	[319]
*Uromyces halimodendri* Solkina	*Halimodendron argenteum* and *H. halodendron*	China and Uzbekistan	[198,220]
*Uromyces handelii* Bubák	*Lotus gebelia*	Iraq	[220,548]
*Uromyces haraeanus* Syd. & P. Syd.	*Scirpus* spp.	China, Japan, and Russia	[90,92,297,549]
*Uromyces hardenbergiae* McAlpine	*Hardenbergia monophylla*	Australia	[280]
*Uromyces hariotianus* Lagerh.	*Odontonema callistachyum*, *Pseuderanthemum cuspidatum*, *Pseuderanthemum* sp., and*Thyrsacanthus strictus*	Costa Rica and México	[218,281,550]
*Uromyces harmsianus* (Henn.) Doidge	*Crotalaria* spp.	India, Kenya, Malawi, Rwanda, South Africa, Tanzania, and Uganda	[138,168,171,172,551]
*Uromyces haussknechtii* Tranzschel	*Euphorbia thamnoide* and *E. pilosa*	India and Syria	[157,350]
*Uromyces hawksworthii* É.S.C. Souza, Z.M. Chaves, W.R.O. Soares, Pinho & Dianese	*Phthirusa stelis*	Brazil	[60]
*Uromyces hedysari-obscuri* (DC.) Carestia & Picc.	*Hedysarum* spp.	Alaska, Austria, Canada, Czech Republic, China, Germany, France, Finland, Hungary, India, Italy, Kazakhstan, Mongolia, Romania, Russia, Switzerland, and Tajikistan	[94,96,220,357,436,535,552,553]
*Uromyces hedysari-paniculati* (Schwein.) Farl.	*Desmodium* spp. and *Meibomia* spp.	America, Argentina, Bolivia, Brazil, Canada, Colombia, Costa Rica, Cuba, Indiana, Jamaica, Massachusetts, México, Trinidad and Tobago, Venezuela, West Indies, and Vermont	[87,88,171,172,185,191,310,415]
*Uromyces heimerlianus* Magnus	*Ervum* spp., *Euphorbia* spp., *Lathyrus* spp., and *Vicia* spp.	Armenia, Austria, Belarus, Bulgaria, China, Czech Republic, France, Germany, Hungary, Italy, Japan, Portugal, Romania, Russia, Serbia, Spain, Switzerland, Ukraine, and Yugoslavia	[220,519,554]
*Uromyces heimii* Mayor & Vienn. Bourg.	*Medicago arborea*	France	[516]
*Uromyces helichrysi* Lagerh.	*Helichrysum plicatum*, *Helichrysum rupestre*, and *Helichrysum siculum*	Algeria, Greece, and Turkey	[80,421,435]
*Uromyces heliotropii* Sred.	*Heliotropium* spp.	Australia, Bulgaria, Cyprus, Greece, Israel, Pakistan, Ukraine, and Uzbekistan	[80,81,111,145,393,421,555,556]
*Uromyces hellebori-thibetani* J.Y. Zhuang & S.X. Wei	*Helleborus thibetanus*	China	[195]
*Uromyces hemmendorfii* Vestergr.	*Bauhinia forficata*	São Paulo	[219]
*Uromyces hermonis* Magnus	*Euphorbia herniariifolia* and *E. peplus*	Greece and Iraq	[196,345,421]
*Uromyces herterianus* Dietel	*Borreria verticillata*, *Diodia* sp. and *Spermacoce verticillata*	Argentina, French Guiana, and Uruguay	[395,415,557]
*Uromyces hessii* Berndt	*Zantedeschia angustiloba*	Angola	[558]
*Uromyces heterantherae* (Henn.) P. Syd. & Syd.	*Heteranthera reniformis*	Brazil	[102,144]
*Uromyces heterodermus* Syd. & P. Syd.	*Erythronium* spp.	Alberta, California, Canada, Columbia, Colorado, Idaho, Montana, Oregon, Texas, Utah, and Washington (USA)	[79,185,187,559]
*Uromyces heterogeneus* Cooke	*Hibiscus syriacus*	India	[126,560]
*Uromyces heteromallus* Syd.	*Haloxylon recurvum*	Pakistan	[208,356,561,562,563]
*Uromyces heteromorphae* Thüm.	*Heteromorpha* spp. and *Peucedanum* spp.	Eritrea, Malawi, and South Africa	[138,273,287,564]
*Uromyces hewittiae* Syd. & P. Syd.	*Hewittia bicolor* and *Hewittia* sp.	Philippines	[565]
*Uromyces hidakaensis* Muray. & Takeuchi	*Pisum sativum*	Japan	[222,566]
*Uromyces himalaicus* Y. Ono, Adhikari & Rajbh.	*Lilium* sp.	Nepal	[567]
*Uromyces hippocrepidis* Syd. & P. Syd.	*Hippocrepis ciliate* and *H. comosa*	Greece, France, Spain, and Switzerland	[220,421,568]
*Uromyces hippomarathri* Lindr.	*Hippomarathrum crispum*, *H. libanotis*, and *H. microcarpum*	Iran and Morocco	[279,317,569]
*Uromyces hippomarathricola* Sousa da Câmara	*Hippomarathrum pterochlaenum*	Portugal	[570]
*Uromyces hobsonii* Vize	*Jasminum* spp.	Euthopia, India, Kenya, and Sri Lanka	[140,571,572]
*Uromyces holci* Jørst.	*Holcus setiger*, *Karroochloa* sp., *Schismus barbatus*, *S. scaberrimus*, and *Tribolium echinatum*	Namibia and South Africa	[124,378]
*Uromyces holubii* Doidge	*Dracaena* sp.	Gauteng	[573]
*Uromyces holwayi* Lagerh.	*Chlorophytum* sp. and *Lilium* spp.	China, California, Canada, Idaho, Iowa, Japan, New York, Nebraska, New Jersey, Oregon, Pakistan, and Washington (USA)	[94,96,187,412,559]
*Uromyces hordeastri* A.L. Guyot	*Bellevalia flexuosa*, *Hordeum* spp., and *Muscari parviflorum*	France and Israel	[81,126]
*Uromyces houstoniatus* J. Sheld.	*Houstonia* spp. and *Sisyrinchium* spp.	Connecticut, Illinois, Kansas, Massachusetts, Missouri, Mississippi, New York, Pennsylvania, Vermont, and Wisconsin (USA)	[191,251]
*Uromyces howei* (Peck) De Toni	*Asclepias curassavica*, *A. guatemalensis*, and *A. nivea*	Bermuda, Cuba, Guatemala, Jamaica, and Peru	[260,281,308,574]
*Uromyces huallagensis* Henn.	*Desmodium* sp.	Peru	[575]
*Uromyces hyacinthi* W. Schneid.	*Hyacinthus fastigiatus*	Canada	[115]
*Uromyces hyalinus* Peck	*Sophora nuttalliana*, *S. sericea*, and *S. stenophylla*	Arizona, Colorado, México, and Wyoming	[116,225,292,559,576]
*Uromyces hybridi* W.H. Davis	*Trifolium hybridum*	Connecticut, India, Massachusetts, Maine, and Vermont	[191,577,578]
*Uromyces hyderabadensis* Ramachar, K.N. Rao & Bagyan.	*Atylosia scarabaeoides*	India	[579]
*Uromyces hydrocotylicola* J.Y. Zhuang	*Hydrocotyle* sp.	China	[96,580]
*Uromyces hymenocarpi* Jaap	*Hymenocarpos circinnatus*	Croatia, Greece, Israel, and Turkey	[80,81,421,581]
*Uromyces hyparrheniae* Gjaerum	*Hyparrhenia* spp.	Angola, Cameroon, Ethiopia, and Uganda	[243,244]
*Uromyces hyparrheniicola* Gjaerum	*Hyparrhenia dregeana*	South Africa	[243]
*Uromyces hyperici* (Schwein.) M.A. Curtis	*Ascyrum hypericoides*, *Elodea* sp., and *Hypericum* spp.	China, California, Florida, Georgia, Indiana, Iowa, Japan, Massachusetts, Mississippi, Missouri, Maine, New York, Texas, Uganda, Vermont, and Wisconsin (USA)	[89,90,186,202,251,262,582,583]
*Uromyces hyperici-frondosi* (Schwein.) Arthur	*Hypericum* spp. and *Triadenum virginicum*	Brazil, Chile, California, Colombia, Guatemala, Maine, Massachusetts, Pennsylvania, South Africa, and Vermont	[78,101,118,119,138,191,281,520,584]
*Uromyces hypericinus* Speg.	*Hypericum brasiliense*	Formosa	[180]
*Uromyces hypoestis* Tarr & G.F. Laundon	*Hypoestes verticillaris*	South Africa, Sudan, Tanzania, and Zimbabwe	[218,250,274]
*Uromyces hypsophilus* Speg.	*Euphorbia* sp.	Mendoza	[391]
*Uromyces ictericus* Cummins	*Iresine celosia* and *Iresine* sp.	Costa Rica and Guatemala	[167,233,310]
*Uromyces ignobilis* (Syd. & P. Syd.) Arthur	*Muhlenbergia* sp. and *Sporobolus* spp.	Barbados, Cuba, Dominica, Grenada, Guyana, Indonesia, Hawaii, México, Pakistan, Texas, and Venezuela	[76,122,171,172,306,309,356,430,431,445,456,559,585,586]
*Uromyces illotus* Arthur & Holw.	*Mucuna andreana*, *M. rubro-aurantiacea*, *M. sloanei*, and *M. urens*	The Dominican Republic, Ethiopia, and Guatemala	[281,406,571]
*Uromyces imperfectus* Arthur	*Bauhinia* spp.	Jamaica, Nicaragua, and Venezuela	[225,308,444,586]
*Uromyces inaequialtus* Lasch	*Cerastium* sp., *Melandrium* sp., and *Silene* sp.	Argentina, Bulgaria, Canada, Chile, China, Finland, Greece, Japan, Nepal, Norway, Romania, Taiwan, South Africa, Sweden, and Ukraine	[82,91,92,97,111,128,135,142,185,188,240,415,421,587]
*Uromyces inayatii* Syd. & P. Syd.	*Apluda aristata* and *A. mutica*	India and Papua New Guinea	[85,426]
*Uromyces indicus* Pat.	*Sporobolus indicus*	Barbados	[588,589]
*Uromyces indigoferae* Dietel & Holw.	*Indigofera* spp.	Argentina, Arizona, Costa Rica, China, Guatemala, Florida, India, México, Panama, Texas, and Venezuela	[90,116,171,172,262,281,292,357,375,415,425,492,559,590]
*Uromyces induratus* Syd., P. Syd. & Holw.	*Dicliptera* spp., *Jacobinia* sp., and *Justicia racemosa*	Argentina, Bolivia, Brazil, Costa Rica, and México	[102,218,292,310,333]
*Uromyces infarctus* Berndt	*Cayaponia* sp.	Costa Rica	[127]
*Uromyces inflatus* (Cooke) McKenzie	*Anisotome* sp.	New Zealand	[591]
*Uromyces ingicola* Henn.	*Inga* sp.	Amazonas	[575]
*Uromyces ingiphilus* Speg.	*Inga edulis*	Argentina	[592]
*Uromyces insignis* P. Syd. & Syd.	*Echinocephalum latifolium* and *Melanthera latifolia*	Brazil	[102,219]
*Uromyces insularis* Arthur	*Clitoria cajanifolia*	Puerto Rico	[466]
*Uromyces intricatus* Cooke	*Chorizanthe* sp., *Eriogonum* spp., and *Gayophytum ramosissimum*	Arizona, Canada, California, Idaho, Oregon, Montana, and Washington (USA)	[185,187,300,412,448]
*Uromyces invisus* (Speg.) Speg.	*Solanum sisymbriifolium*	Argentina	[415,592]
*Uromyces ipatingae* F.A. Ferreira & Y. Hirats.	*Clitoria fairchildiana*	Brazil	[219,593]
*Uromyces iresines* Lagerh.	*Iresine* spp.	Argentina, Colombia, Dominican Republic, Ecuador, Guatemala	[118,119,144,167,281]
*Uromyces isachnes* Petch	*Isathne kunthiana*	Sri Lanka	[594]
*Uromyces itoanus* Hirats. f.	*Kummerowia* stipulacea, *K. striata*, and *Microlespedeza* spp.	China, Korea, Japan, and Taiwan	[89,90,483,595,596]
*Uromyces ixiae* (Lév.) G. Winter	*Acidanthera* spp., *Antholyzasp*., *Babiana* spp., *Engysiphon* sp., *Geissorhiza* sp., *Gladiolus* spp., *Hesperantha* spp., *Ixia* spp., *Lapeirousia* spp., *Melasphaerula* spp., *Romulea* sp., and *Sparaxis* spp.	Kenya and South Africa	[123,140]
*Uromyces jacksonii* Arthur & Fromme	*Agrostis* spp., *Deschampsia* spp., *Festuca* spp., and *Hordeum* spp.	California, Idaho, Oregon, Michigan, and Washington (USA)	[241,559]
*Uromyces jamaicensis* Vestergr.	*Bauhinia* spp.	Cuba, El Salvador, Jamaica, México, Puerto Rico, and Venezuela	[184,216,292,306,308,444,541]
*Uromyces janiphae* (G. Winter) Arthur	*Jatropha* spp. and *Manihot* sp.	Brazil, Colombia, Costa Rica, Cuba, Ecuador, Panama, Puerto Rico, México, and Venezuela	[76,118,119,125,431,456,511,597]
*Uromyces japonicus* Berk. & M.A. Curtis	*Orchidaceae*	Bulgaria, China, Italy, Japan, Poland, Russia, and Ukraine	[51,82,96,135,160,598,599]
*Uromyces jatrophae* Dietel & Holw.	*Jatropha multifida*	Brazil, Colombia, Cuba, México, Jamaica, Puerto Rico, and Virgin Islands	[102,103,184,227,308,479,597]
*Uromyces jatrophicola* Henn.	*Cnidoscolus* sp. and *Jatropha* sp.	Brazil	[103,600]
*Uromyces joffrinii* Delacr.	*Vanilla planifolia*	France	[476]
*Uromyces johowii* Dietel & Neger	*Vicia macraei*, *V. nigricans*, and *Vicia* sp.	Chile	[282,494]
*Uromyces jonesii* Peck	*Ranunculus* sp.	California, Colorado, Montana, and Wyoming	[187,300,368,412,601]
*Uromyces jordianus* Bubák	*Euphorbia cyparissias*	Austria, Bulgaria, Czech Republic, Germany, Hungary, Romania, Russia, Switzerland, and Ukraine	[82,220,602]
*Uromyces junci* Tul.	*Ambrosia psilostachya*, *Arnica* spp., *Aster* spp., *Bahia dissecta*, *Cirsium* spp., *Eriophyllum* sp., *Grindelia* sp., *Helianthus* spp., *Jaumea* spp., *Juncus* spp., *Juniperus litoralis*, *Luzula alopecurus*, and *Pulicaria dysenterica*	Arizona, Bolivia, Bulgaria, Canada, California, Chile, Colorado, Florida, Germany, Idaho, Israel, Japan, North and South Dakota, Nebraska, Montana, Oklahoma, Oregon, Pennsylvania, Poland, Romania, Spain, Wyoming, and Wisconsin (USA)	[81,82,105,116,135,185,187,262,377,386,559,603]
*Uromyces juncicola* Speg.	*Juncus stipulatus*	Mendoza	[180]
*Uromyces junci-effusi* P. Syd. & Syd.	*Juncus* spp.	Arizona, California, Canada, Idaho, Florida, Georgia, Maine, Missouri, Montana, Oregon, Utah, Washington, and Wyoming	[114,116,144,185,187,191,202,262,559]
*Uromyces juncinus* Thüm.	*Junco acutiflori*	Italy	[604]
*Uromyces kaernbachii* Henn.	*Abrus precatorius*	Papua New Guinea	[136]
*Uromyces kaimontanus* Hirats. f. & S. Sato	*Veratrum album*, *V. grandiflorum*, *V. nigrum*, and *V. puberulum*	China, Japan	[222,605]
*Uromyces kalmusii* Sacc.	*Euphorbia* sp.	China, Germany, Poland, and Russia	[105,135,606,607]
*Uromyces karjaginii* Uljan.	*Seseli cuneifolium*	Azerbaijan	[608]
*Uromyces kawakamii* Syd. & P. Syd.	*Euphorbia serrulata*	China, Japan, and Taiwan	[94,97,108,446]
*Uromyces kentaniensis* Doidge	*Antholyza aethiopica* and *Chasmanthe aethiopica*	South Africa	[249,322]
*Uromyces kenyensis* J.F. Hennen	*Chloris gayana*, *C. myriostachya*, and *C. roxburghiana*	Kenya, Uganda, Zimbabwe	[171,242,460]
*Uromyces kigesianus* Cummins	*Pittosporum abyssinicum*	Uganda	[609]
*Uromyces kisantuensis* Henn.	*Dolichos* sp.	The Democratic Republic of the Congo	[610]
*Uromyces klebahnii* E. Fisch.	*Astragalus* spp. and *Oxytropis* sp.	Austria, Bulgaria, France, Italy, Romania, Russia, Switzerland, and Turkey	[80,82,220]
*Uromyces klotzschianus* B. Ali	*Rumex dentatus*	Pakistan	[61]
*Uromyces kochiae* Syd. & P. Syd.	*Kochia prostrata*	China, Russia, Turkey, and Uzbekistan	[144,146,198,402]
*Uromyces kochianus* Gäum.	*Geranium nodosum*	Switzerland	[611]
*Uromyces koeleriae* Uljan.	*Koeleria caucasica*	Russia	[106]
*Uromyces komarowii* Bubák	*Solidaginis virgaureae*	Czech Republic	[612]
*Uromyces kondoi* Miura	*Amblytropis multiflora*	China, Japan, and Pakistan	[89,156,356]
*Uromyces krameriae* Arthur	*Krameria* spp.	Florida and Texas	[262,281]
*Uromyces krantzbergensis* Doidge	*Anthericum* sp.	Namibia	[138]
*Uromyces kunigamiensis* Shimab.	*Fimbristylis dichotoma* and *Fimbristylis* sp.	China and Japan	[108,222]
*Uromyces kurtzii* Henn.	*Senecio* spp.	Argentina	[415,613]
*Uromyces kwangensis* Henn.	*Justicia* sp.	Congo	[218,610]
*Uromyces kwangsianus* Cummins	*Fimbristylis* sp.	China and Taiwan	[614,615]
*Uromyces lachenaliae* Doidge	*Lachenalia* spp. and *Polyxena ensifolia*	South Africa	[123,138]
*Uromyces laevigatus* Syd. & P. Syd.	*Aneilema* spp.	Ghana and Tanzania	[318,549,616]
*Uromyces laevis* Körn.	*Euphorbia* spp.	Bulgaria, Germany, Iran, Romania, Turkey, and Ukraine	[80,82,105,111,228,617,618]
*Uromyces langtangensis* Durrieu	*Anaphalis nepalensis*	Nepal	[463]
*Uromyces lapeyrousiae* Petr.	*Lapeyrousia*	Tanzania	[231]
*Uromyces lapponicus* Lagerh.	*Astragalus* spp. and *Oxytropis* spp.	Alaska, Canada, China, Colorado, Finland, Idaho, India, Iran, Japan, Mongolia, Norway, Oregon, Pakistan, Russia, Sweden, and Turkey	[80,96,128,129,130,131,357,368,436,449,535,619]
*Uromyces largus* Arthur & Cummins	*Chamaesyce lata*	Colorado	[87,88]
*Uromyces laserpitii-graminis* E. Fisch.	*Laserpitii sileris* and *Melica ciliata*	Southern Europe and Northern Africa	[106]
*Uromyces lasiocorydis* Henn.	*Lasiocorys abyssinica*	Eritrea	[287,426]
*Uromyces lathyrinus* Speg.	*Lathyrus* spp. and *Vicia* spp.	Argentina, Brazil, Chile, and Paraguay	[100,101,102,219,620]
*Uromyces latimammatus* J.Y. Zhuang & S.X. Wei	*Ipomoea sumatrana*	China	[621]
*Uromyces lazistanicus* Petr.	*Orobus roseus*	Armenia and Turkey	[220]
*Uromyces lenticola* Petr.	*Lens esculenta*	Iran	[430]
*Uromyces leonotidis* Bagyan., Gjaerum & M. Raju	*Leonotis nepetifolia*	India	[622]
*Uromyces leptaleus* Syd.	*Stellaria laxa*	China, Japan, Philippines, and Taiwan	[89,90,97,565,623]
*Uromyces leptochloae* Wakef.	*Leptochloa obtusiflora*	Kenya and Uganda	[140,168]
*Uromyces lereddei* Dupias	*Colutea arborescens*	France	[220,624]
*Uromyces lespedezae* (Thüm.) Peck	*Lespedeza capitata*	Vermont	[583]
*Uromyces lespedezae-bicoloris* F.L. Tai & C.C. Cheo	*Lespedeza bicolor* and *L. formosa*	China	[108,109,625]
*Uromyces lespedezae-macrocarpae* Liou & Y.C. Wang	*Campylotropis macrocarpa*, *Lespedeza bicolor*, and *L. formosa*	China	[108,109]
*Uromyces lespedezae-procumbentis* (Schwein.) Lagerh.	*Campylotropis* spp., *Kummerowia* spp., and *Lespedeza* spp.	Canada, China, Georgia, India, Japan, Korea, Missouri, Taiwan, and Pennsylvania	[94,108,109,185,202,255,357,482,596]
*Uromyces lespedezae-sericeae* S. Ahmad	*Lespedeza sericea*	Pakistan	[208]
*Uromyces libycus* Trotter	*Lotus pusillus*	Libya	[197,626]
*Uromyces ligulariae* Hirats. & Hoshioka	*Ligularia tussilaginea* var. *formosana*	China, Japan, Taiwan	[94,97,109,627]
*Uromyces limonii-caroliniani* Savile & Conners	*Limonium carolinianum*	Canada, Mississippi, and Texas	[185,559,628]
*Uromyces limosellae* F. Ludw.	*Limosella* sp.	Australia	[280]
*Uromyces loculatus* Cummins	*Kyllinga alba*	Zambia	[341]
*Uromyces loculiformis* T.S. Ramakr. & K. Ramakr.	*Chlorophytum attenuatum*	China, India, and Nepal	[96,464,629]
*Uromyces lomandracearum* J. Walker & van der Merwe	*Lomandra longifolia* and *Lomandra* sp.	Australia	[62,630]
*Uromyces longipedicellaris* Ramachar & A. Sudh. Rao	*Rumex vesicarius*	Pakistan	[631]
*Uromyces longipes* (Lasch) Tranzschel	*Pedicularis* sp.	Russia	[382]
*Uromyces loranthi* H.S. Jacks.	*Loranthus*	Brazil	[376]
*Uromyces lotononidicola* Berndt	*Lotononis cytisoides*	South Africa	[632]
*Uromyces lugubris* Kalchbr.	*Euphorbia cyparissias*	South Africa	[137]
*Uromyces lupini* Berk. & M.A. Curtis	*Lupinus* spp.	Arizona, California, Canada, Colorado, México, Montana, Oregon, Washington, and Wyoming	[116,185,187,368,412,454,598]
*Uromyces lupinicola* Bubák	*Lupinus* spp.	Bulgaria, Finland, Lithuania, Norway, and Spain	[158,363,587,612]
*Uromyces lychnidis* Tracy & Earle	*Lychnidis drummondii*	Utah	[633]
*Uromyces lygei* P. Syd. & Syd.	*Lygeum spartum*	Sardegna	[325]
*Uromyces macnabbii* Cummins	*Chionochloa* spp. and *Danthonia* spp.	New Zealand	[284,591]
*Uromyces macounianus* Ellis & Everh.	*Euphorbia maculata* var. *affinis* and *E. serpyllifolia*	Canada and British Columbia	[103,388,634]
*Uromyces macowanii* Bubák	*Scilla prasina*	Africa	[612]
*Uromyces magnatus* (Arthur) Arthur	*Polygonatum* sp. and *Vagnera* sp.	Illinois, Iowa, Minnesota, Montana, Nebraska, North and South Dakota, and Wisconsin	[306,307]
*Uromyces magnusii* Kleb.	*Medicago minima*	China, Turkey, and Ukraine	[80,96]
*Uromyces maireanus* P. Syd. & Syd.	*Ornithogalum* spp.	Morocco, South Africa, Tanzania	[142,144,318]
*Uromyces major* Arthur	*Muhlenbergia reverchonii*, *Muhlenbergia* sp., and *Sporobolus indicus*	Belize, México, Texas, Trinidad, and Tobago	[112,117,635]
*Uromyces malloti* Henn.	*Mallotus moluccanus* and *Melanolepismulti glandulosa*	Guinea, New Papua, and the Philippines	[136,177]
*Uromyces mangenotii* Mayor & Vienn. Bourg.	*Vicia pubescens*	France	[516]
*Uromyces manihoticola* Henn.	*Manihot* spp.	Brazil	[102,103,291]
*Uromyces manihotis* Henn.	*Manihot* spp.	Brazil, Colombia, Cuba	[102,227,291]
*Uromyces manihotis-catingae* Henn.	*Manihot* spp.	Brazil	[102,600]
*Uromyces marinus* Guyot & Malençon	*Medicago marina*	Morocco	[220,279]
*Uromyces martinii* Farl.	*Melanthera* spp., *Bidens* spp.	Florida	[225,262,636]
*Uromyces massoniae* Doidge	*Massonia latifolia*	South Africa	[138]
*Uromyces mayorii* Tranzschel	*Euphorbia* spp.	California and Colombia	[227,412]
*Uromyces mbelensis* Henn.	*Indigofera*	The Democratic Republic of the Congo	[610]
*Uromyces megalosporus* Speg.	*Tessaria absinthioides*	Tucumán	[371]
*Uromyces meimerlianus* Magnus	*Vicia* sp.	Germany	[531]
*Uromyces melandrii* Dietel & Neger	*Melandrium cucubaloides*	Los Lagos	[162]
*Uromyces melantherae* Cooke	*Melanthera brownii*	Ghana, South Africa, and Uganda	[138,168,616]
*Uromyces melasphaerulae* Syd. & P. Syd.	*Melasphaerula graminea*	Western Cape Province	[424]
*Uromyces melothriae* Henn.	*Melothria tomentosa*	Eritrea	[287,427]
*Uromyces menyanthis* Azbukina & Zenkova	*Menyanthes trifoliata*	Primorye	1966 [637]
*Uromyces mercurialis* Henn.	*Mercurialis leiocarpa*	China, Japan, and Taiwan	[94,96,97]
*Uromyces mexicanus* Dietel & Holw.	*Desmodium*	Arizona, Costa Rica, México	[116,171,310,479]
*Uromyces meygounensis* Petr.	*Euphorbia bungei*	Iran	[638]
*Uromyces microchloae* Syd. & P. Syd.	*Microchloa setacea*	Bolivia, Brazil, Central Africa, Sudan, and Uganda	[106,168,199,394]
*Uromyces microsorus* Kalchbr. & Cooke	*Prunus armeniaca*	South Africa	[137]
*Uromyces microtidis* Cooke	*Microtis porrifolia*	Australia and New Zealand	[245,313,639]
*Uromyces miersiae* Gäum.	*Miersia chilensis*	Chile	[540]
*Uromyces mikaniae* Viégas	*Mikania* sp.	Brazil	[219,640]
*Uromyces mimusops* Cooke	*Mimusops* sp.	South Africa	[641]
*Uromyces minimus* Davis	*Muhlenbergia sylvatica*	Canada, Michigan, Oregon, and Wisconsin	[185,635]
*Uromyces minor* J. Schröt.	*Medicago denticulata*, *Pisum sativum*, and *Trifolium* spp.	Armenia, Austria, Bulgaria, California, Caucasus, Chile, China, Czech Republic, Estonia, Finland, France, Germany, Hungary, India, Montana, Pakistan, Poland, Romania, Russia, Spain, Sweden, Switzerland, Texas, Ukraine, New Zealand, Oregon, Washington, and Wyoming	[220,313,355,357,559,642]
*Uromyces minutus* Dietel	*Carex* spp.	Alabama, Central and Eastern United States, Iowa, Mississippi, Texas, and Wisconsin	[255,475,559,643,644]
*Uromyces miurae* Syd. & P. Syd.	*Fritillaria kamtschatcensis*	Alaska, Canada, Idaho, Japan, Russia, and Washington (USA)	[94,185,187,645,646]
*Uromyces moehringiae* S. Ito & Hirats. f.	*Moehringia lateriflora*	Japan	[94,297]
*Uromyces moesiacus* Lindtner & Vienn. Bourg.	*Lathyrus nissolia*	Yugoslavia	[451]
*Uromyces mogianensis* Bubák	*Fritillaria graeca*, *F. rhodocanakis*, *Rhinopetalum arianum*, *R. gibbosum*, and *R. karelinii*	Afghanistan, Greece, and Uzbekistan	[421,555,612]
*Uromyces mongolicus* U. Braun & G. Hirsch	*Euphorbia kozlovii*	Mongolia	[535,647]
*Uromyces monspessulanus* Tranzschel	*Euphorbia serrata*	Balearic Islands, Libya, and Spain	[129,157,197,648]
*Uromyces montanoae* Arthur & Holw.	*Montanoa dumicola*, *M. pittieris*, *M. hibiscifolia*, and *Montanoa* sp.	Costa Rica and Guatemala	[225,281,310]
*Uromyces montanus* Arthur	*Lupinus mexicanus*	Guatemala and México	[281,398]
*Uromyces montis-ferrati* Maire	*Euphorbia luteola*	Northern Africa	[649]
*Uromyces moraeae* Syd. & P. Syd.	*Moraea spathacea*	South Africa	[138,144]
*Uromyces mucunae* Rabenh.	*Mucuna pruriens*	China, Cambodia, Japan, India, Indonesia, Madagascar, Malawi, Mauritius, Taiwan, Tanzania, South Africa, Guinea, Philippines, Thailand, and Uganda	[97,209,273,309,465,565,605,650,651,652]
*Uromyces muhlenbergiae* S. Ito	*Muhlenbergia japonica*	Japan	[635]
*Uromyces mulgedii* Lindr.	*Lactuca* sp.	The Czech Republic and Turkey	[569]
*Uromyces mulini* J. Schröt.	*Mulinum integrifolium*	Argentina and Chile	[240,331,415]
*Uromyces musae* Henn.	*Musa* spp.	Congo, Fiji, and Philippines	[565,610,653,654]
*Uromyces muscari* (Duby) Niess	*Bellevalia* spp., *Chionodoxa* spp., *Dipcadi erythraeum*, *Endymionspp*., *Gagea* sp., *Hyacinthoides* spp., *Leopoldia* spp., *Muscari* spp., *Ornithogalum* sp., *Scilla* spp., and *Urginea* spp.	Afghanistan, Bulgaria, California, Canada, Germany, Greece, India, Israel, Iran, Italy, Morocco, New Zealand, Norway, Scotland, Sweden, and Turkey	[80,81,82,105,123,124,179,317,318,389,534,655]
*Uromyces mussooriensis* Syd. & P. Syd.	*Stipa sibirica*	India	[106,656]
*Uromyces myosotidis* Bahç.	*Myosotis* sp.	Turkey	[471]
*Uromyces myristica* Berk. & M.A. Curtis	*Euphorbia bicolor*	Texas	[657]
*Uromyces myrsines* Dietel	*Ardisia compressa*, *Icacorea* sp., *Myrsine* spp., and *Rapanea* spp.	Bolivia, Brazil, Costa Rica, and Uruguay	[100,101,143,310]
*Uromyces mysticus* Arthur	*Hordeum jubatum*	California, Canada, Colorado, Idaho, Israel, and Utah	[81,112,114,185,412,559]
*Uromyces namaqualandus* Mennicken, W. Maier & Oberw.	*Roepera cordifolia*	Namibia	[384]
*Uromyces nassauviae* J.C. Lindq.	*Nassauvia lagascae*	Argentina	[415]
*Uromyces nassellae* Cummins	*Nassella pubiflora*	Bolivia	[106]
*Uromyces natalensis* Magnus	*Euphorbia* spp.	Madagascar and South Africa	[169,465]
*Uromyces natricis* A.L. Guyot	*Ononis rotundifolia*	France	[342]
*Uromyces nattrassii* Cummins	*Statice spicata*	Cyprus	[350]
*Uromyces naucinus* Berndt	*Cayaponia* sp.	Ecuador	[127]
*Uromyces necopinus* Cummins	*Hypoxis hirsuta*	Connecticut, Massachusetts, and New York	[167,233]
*Uromyces neotropicalis* J.R. Hern. & Aime	*Cayaponia rigida*, *C. selysioides*, and *Cucurbita* sp.	Guiana and Guyana	[63,557]
*Uromyces nevadensis* Harkn.	*Primula suffrutescens*	California	[78,658]
*Uromyces nidificans* Tranzschel	*Aellenia subaphylla*, *Cimacoptera korshinskyi*, *Salsola arbuscula*, and *Salsola* sp.	Central Asia, Iran, and Uzbekistan	[146,161,346]
*Uromyces niteroyensis* Rangel	*Panicum* spp. and *Setaria* spp.	Argentina, Brazil, Colombia, India, French Guiana, and México	[102,110,395,415,659]
*Uromyces nordenskjoldii* Dietel	*Vicia darapskyana* and *Vicia* sp.	Argentina and Chile	[415]
*Uromyces notabilis* Wakef.	*Cyperus* sp. and *Kyllinga* sp.	Uganda	[168,462]
*Uromyces nothoscordi* Syd. & P. Syd.	*Nothoscordum striatum*	Texas	[660]
*Uromyces novissimus* Speg.	*Trianosperma ficifolia*	Argentina, Brazil, Colombia, Panama, México, and West Indies	[48,324,469]
*Uromyces numidicus* Maire	*Geranium atlanticum*	Northern Africa	[649]
*Uromyces nyikensis* Syd. & P. Syd.	*Gladiolus nyikensis*	Malawi	[424]
*Uromyces nymphoidis* Săvul.	*Nymphoides peltata*	Romania	[228]
*Uromyces oaxacanus* Dietel & Holw.	*Jatropha urens*	Arizona, Belize, Guatemala, and México	[103,116,281]
*Uromyces oberwinklerianus*Berndt	*Acalypha* sp.	Costa Rica	[310]
*Uromyces obesus* Durrieu	*Heteropogon contortus*	Nepal and Uganda	[244,464]
*Uromyces oblectaneus* H.S. Jacks. & Holw.	*Rhynchospora corymbosa*, *R. exaltata*, and *Rhynchospora* sp.	Brazil	[166,219]
*Uromyces oblongisporus* Ellis & Everh.	*Artemisia tridentata*	China and Wyoming	[225,661]
*Uromyces oblongus* Vize	*Medicago polymorpha* and *Trifolium* spp.	California and Washington	[78,454,662]
*Uromyces obscurus* Dietel & Holw.	*Phaseolus* sp.	México	[456,479]
*Uromyces occidentalis* Dietel	*Euphorbia* spp., *Lupinus* spp., and *Tithymalus* spp.	Arizona, California, Colorado, México, Montana, Nevada, Nigeria, Oregon, and Utah	[114,116,187,292,316,412,663]
*Uromyces occultus* J.C. Lindq.	*Juncus densiflorus* var. *pohlii*	Argentina, Brazil, Chile, and México	[102,486,664]
*Uromyces ocimi* Hansf.	*Ocimum menthifolium*	Uganda	[168]
*Uromyces oedipus* Dietel	*Sophora japonica*	Japan	[665]
*Uromyces oenotherae* Burrill	*Oenothera linifolia*	Illinois	[582]
*Uromyces oliveirae* J. Anikster & I. Wahl	*Bellevalia eigii*	Israel	[390]
*Uromyces ononidis* Pass.	*Euphorbia* spp. and *Ononis* spp.	Bulgaria, Cyprus, Greece, Israel, Italy, Libya, Poland, Morocco, Romania, Spain, and Turkey	[80,81,82,105,111,220,532,666,667]
*Uromyces ophiorrhizae* Gäum.	*Ophiorrhiza longiflora*	Indonesia	[309]
*Uromyces orbicularis* Dietel	*Desmodium* spp.	Argentina, Brazil, and Bolivia	[143,171,269]
*Uromyces orchidearum* Cooke & Massee	*Chiloglottis* spp.	Australia	[268]
*Uromyces orientalis* Syd. & P. Syd.	*Indigofera* spp.	Australia, Ethiopia, Ghana, India, Japan, Kenya, Pakistan, Malawi, Philippines, Tanzania, Uganda, and Zambia	[85,168,171,172,357]
*Uromyces ornatipes* Arthur	*Phrygilanthus sonorae*	México	[15,76,77]
*Uromyces ornithogali* (Wallr.) Niessl	*Gagea arvensis*, *Ornithopus nanum*, and *Ornithopus* sp.	Romania, Portugal, Spain, and Ukraine	[111,158,179,228]
*Uromyces ornithopodioides* Gonz. Frag	*Ornithopus isthmocarpus* and *O. compressus*	Portugal	[317]
*Uromyces orobi-tuberosi* (Pers.) Liro	*Gladiolus* sp.	Finland	[668]
*Uromyces orthosiphonis* T.S. Ramakr. & Sriniv.	*Orthosiphon glabratus*	India	[152,669]
*Uromyces otakou* G. Cunn.	*Poa* spp.	New Zealand	[591,670]
*Uromyces otaviensis* Mennicken, W. Maier & Oberw.	*Ipomoea verbascoidea*	Namibia	[385]
*Uromyces ovalis* Dietel	*Leersia oryzoides*	Japan	[671]
*Uromyces ovirensis* Jaap	*Primula wulfeniana*	Austria	[672]
*Uromyces pachyceps* Lagerh.	*Ipomoea* sp.	Brazil, Ecuador	[126]
*Uromyces pallidus* Niessl	*Chamaecytisus* spp., *Cytisus* spp., and *Lembotropis nigricans*	Austria, Belarus, Bulgaria, Czech Republic, Germany, Hungary, Italy, Poland, Romania, Ukraine, and Yugoslavia	[105,135,220,673]
*Uromyces panici-sanguinalis* Rangel	*Digitaria horizontalis*, *Panicum sanguinale*, and *Panicum* sp.	Brazil, Cuba, Grenada, and Venezuela	[171,674,675]
*Uromyces pannosus* Vestergr.	*Bauhinia candicans*	Brazil	[216]
*Uromyces papillatus* Kalchbr. & Cooke	*Heteromorpha arborescens*	South Africa	[137]
*Uromyces paradoxus* Syd. & P. Syd.	*Commiphora zimmermannii* and *Commiphora* sp.	Kenya and Mozambique	[138,140,676]
*Uromyces parilis* Syd.	*Rumex occultans*	Israel	[81]
*Uromyces paspalicola* Arthur & Holw.	*Paspalum racemosum*	Ecuador	[199,294]
*Uromyces paulshoekensis* Mennicken, W. Maier & Oberw.	*Roepera foetida*	South Africa and Northern Cape Province	[384]
*Uromyces pavgii* R.N. Goswami & Ngachan	*Achyranthes aspera*	India	[677]
*Uromyces pavoniae* Arthur	*Pavonia racemosa*	Puerto Rico	[259,678]
*Uromyces pazschkeanus* Henn.	*Vigna* sp.	Eritrea	[287,427]
*Uromyces peckianus* Farl.	*Aristida* spp., *Atriplex* spp., *Bryzopyrum* sp., *Chenopodium* spp., *Distichlis* spp., *Houstonia* spp., *Plantago* spp., and *Salicornia* spp.	Alabama, California, Canada, Florida, Oklahoma, Massachusetts, Missouri, Montana, New York, Texas, and Wisconsin (USA)	[185,187,262,455,475,559,636]
*Uromyces peglerae* Pole-Evans	*Digitaria* spp.	Kenya, India, Malavi, Mauritius, New Guinea, Pakistan, Philippines, South Africa, Uganda, and Zimbabwe	[171,172,232,441,447]
*Uromyces peireskiae* Dietel	*Peireskia grandifolia*, *P. sacharosa*, and *Peireskia* sp.	Argentina and Brazil	[394,415]
*Uromyces pencanus* (Dietel & Neger) Arthur & Holw.	*Nassella* spp. and *Stipa* spp.	Argentina, Australia, Bovilia, Chile, andNew Zealand	[24,199,415]
*Uromyces penniseti* S. Ahmad	*Pennisetum lanatum*	Pakistan	[536,679]
*Uromyces pentaceae* D.K. Agarwal	*Pentace burmanica*	India	[680]
*Uromyces pentaschistidis* Gjaerum	*Pentaschistis airoides*	South Africa	[243]
*Uromyces peracarpae* S. Ito & Tochinai	*Peracarpa carnosa* and *P. circaeoides*	Japan and Russia	[94,297]
*Uromyces peraffinis* Dietel	*Bauhinia* sp.	Brazil	[219]
*Uromyces pereskiae* H.S. Jacks. & Holw.	*Pereskia aculeata*, *P. grandifolia*, and *Pereskia* sp.	Argentina and Brazil	[269,681]
*Uromyces perigynius* Halst.	*Carex* spp., *Ratibida columnaris*, *Rudbeckia* spp., and *Solidago* spp.	Bermuda, Canada, Idaho, Iowa, Maine, Missouri, Montana, Oregon, Pennsylvania, Washington, and Wisconsin	[185,187,191,252,260]
*Uromyces perlebiae* Vestergr.	*Bauhinia* spp.	Brazil	[102,216]
*Uromyces permeritus* Cummins	*Tournefortia sarmentosa* and *Tournefortia* sp.	Papua New Guinea	[223,426]
*Uromyces persicus* Syd. & P. Syd.	*Astragalus* spp., *Oxytropis* spp., and *Phaca* spp.	Alaska, Alberta, Austria, Canada, Colorado, India, Iran, Japan, Kazakhstan, Norway, Oregon, Russia, Siberia, Sweden, Tajikistan, and Turkmenistan	[220,357,682]
*Uromyces petitmenginii* Maire	*Minuartia globulosa* and *M.* mereyi	Greece, Turkey	[80,421,470]
*Uromyces phacae-frigidae* (Wahlenb.) Har.	*Astragalus* spp. and *Phaca* spp.	Alaska, Canada, Caucasus, Kyrgyzstan, Pakistan, Norway, Russia, and Sweden	[367,436,553,646]
*Uromyces phalaridicola* Katajev	*Phalaris minor*	Turkmenistan	[106]
*Uromyces phaseolicola* Speg.	*Phaseolus prostratus*	Argentina	[126,180]
*Uromyces phlei-michelii* Cruchet	*Phleum alpinum*, *P. phleoides*, and *P. michelii*	Morocco and Switzerland	[126,279]
*Uromyces phlogacanthi* Gäum.	*Phlogacanthus celebicus*	Indonesia	[309,683]
*Uromyces phtirusae* Mayor	*Phthirusa pyrifolia*	Colombia	[15,226]
*Uromyces phyllachoroides* Henn.	*Cynosurus elegans*	Tunisia	[684]
*Uromyces physanthyllidis* Vienn.-Bourg.	*Physanthyllis tetraphylla*	Greece	[544]
*Uromyces phyteumatum* (DC.) Niessl	*Phyteuma* spp.	The Czech Republic, Denmark, France, Germany, Norway, Poland, Romania, and Spain	[128,158,159,228,440]
*Uromyces pianhyensis* Henn.	*Wedelia* spp.	Brazil, Ethiopia, India, Puerto Rico, Virgin Islands, and West Indies	[264,312,571,600]
*Uromyces pictus* Thüm.	*Abutilon elaeocarpoides* and *Abutilon* sp.	Galapagos Islands and Ethiopia	[571,685,686]
*Uromyces pieningii* Cummins	*Ipomoea argentaurata* and *Ipomoea pes-caprae*	Ghana and Indonesia	[126,341]
*Uromyces pisi-sativi* (Pers.) Liro	*Astragalus* spp., *Colutea* sp., *Cytisus scoparius*, *Euphorbia* sp., *Galega officinalis*, *Genista* spp., *Lathyrus* spp., *Lotus* spp., *Medicago* spp., *Onobrychis* spp., *Pisum* spp., *Trifolium pratense*, *Ulex europaeus*, and *Vicia* sp.	Finland, Germany, Hong Kong, Israel, Libya, Turkey, Serbia, and Uzbekistan	[80,81,329]
*Uromyces pittospori* Henn.	*Pittosporum abyssinicum*	Eritrea	[287,687]
*Uromyces planiusculus* (Mont.) Jørst.	*Rumex frutescens*	Tristan da Cunha	[688]
*Uromyces plantaginis* Vestergr.	*Plantago barbata* and *P. tubulosa*	Argentina	[165,415]
*Uromyces plumbarius* Peck	*Gaura* spp., *Oenothera* spp., *Onagra biennis*, and *Pachylophus marginatus*	California, Canada, Colorado, Connecticut, Florida, Idaho, Louisiana, Iowa, México, Minnesota, Mississippi, Oregon, Texas, and Utah	[114,155,185,187,262,475,559]
*Uromyces poae-alpinae* Rytz	*Poa alpina* and *Ranunculus montanus*	Germany and Poland	[135,160]
*Uromyces poinsettiae Speg.*	*Poinsettia heterophylla*	Argentina	[689]
*Uromyces poiretiae* Syd.	*Poiretia scandens*	Venezuela	[431,690]
*Uromyces polemanniae* Kalchbr. & Cooke	*Polemannia* spp.	South Africa	[137,142]
*Uromyces poliotelis* Syd.	*Anguria* sp., *Gurania* sp., and *Selysia prunifera*	Costa Rica	[127,229]
*Uromyces politus* (Berk.) McAlpine	*Muehlenbeckia cunninghamii*	Australia	[280,343]
*Uromyces polycnemi* McAlpine	*Polycnemum pentandrum*	Bulgaria, Britain, Iran, and Ukraine	[82,280,317,422]
*Uromyces polygalae* Grove	*Polygala* spp.	Mongolia and Uganda	[535,691]
*Uromyces polygoni-avicularis* (Pers.) G.H. Otth	*Polygonum nepalense*	Nepal	[692,693]
*Uromyces polymniae* (Henn.) Dietel & Holw.	*Polymnia* spp.	Argentina, Brazil, Colombia, Guatemala, and México	[219,269,281,375]
*Uromyces polymorphus* Peck & Clinton	*Lathyri* sp.	New York	[694]
*Uromyces polytriadicola* Arthur & Cummins	*Polytrias amaura*	Philippines	[232]
*Uromyces pontederiae* W.R. Gerard	*Pontederia cordata*	Argentina, Brazil, Delaware, Florida, Georgia, Missouri, New York, Pennsylvania, Texas, and Virginia	[415,559,695]
*Uromyces pontederiicola* Speg.	*Pontederia sagittata*	Argentina and India	[34,592]
*Uromyces poonensis* W.D. More & Moniz	*Sesbania aegyptiaca*, *S. grandiflora*, and *S. sesban*	India	[357,696]
*Uromyces porcensis* Mayor	*Inga ingoides*	Colombia	[226]
*Uromyces porosus* (Peck) H.S. Jacks.	*Vicia americana* and *V. sparsifolia*	Iowa	[155,295,454]
*Uromyces pozoae* Dietel & Neger	*Pozoa hydrocotylifolia*	Chile	[240,417]
*Uromyces praetextus* Vestergr.	*Bauhinia* sp.	Brazil	[216]
*Uromyces prangi* Har.	*Hippomarathrum cristatum* and *Prangos* sp.	Bulgaria and Iran	[82,347]
*Uromyces pratensis* Juel	*Poa pratensis*, *Ranunculus auricomus*, and *R. cassubicus*	Finland	[329]
*Uromyces pratiae* Speg.	*Hypsela reniformis* and *Pratia repens*	Argentina, Brazil, and Ecuador	[123,219,415]
*Uromyces pretoriensis* Doidge	*Commelina africana*	Ghana, Uganda, Namibia, and South Africa	[138,168,443]
*Uromyces primaverilis* Speg.	*Allium striatellum*	Argentina, Illinois, Kansas, Missouri, Michigan, Missouri, Texas, Oklahoma, and Uruguay	[305,415,455,542,559]
*Uromyces primulae-integrifoliae* (DC.) Niessl	*Primula deorum* and *P. integrifolia*	Bulgaria and Switzerland	[82,159]
*Uromyces prismaticus* Vienn. Bourg.	*Secale montanum*	Iran	[320]
*Uromyces privae* Syd. & P. Syd.	*Priva lappulacea*	Cuba and Venezuela	[697,698]
*Uromyces probus* Arthur	*Olsynium grandiflorum* and *Sisyrinchium* spp.	Canada, California, Idaho, Oregon, Texas, Utah, and Washington	[66,112,185,187,559]
*Uromyces procerus* J.C. Lindq.	*Festuca procera*	Chile	[240]
*Uromyces propinquus* P. Syd. & Syd.	*Desmodium* sp. and *Rhopalotria mollis*	Mexico	[325]
*Uromyces prosopidis* (Jacz.) Jacz.	*Prosopis farcta*	Iran	[699]
*Uromyces pseudarthriae* Cooke	*Pseudarthria robusta*	South Africa	[700]
*Uromyces psoraleae* Peck	*Psoralea lanceolata*	Arizona, Canada, Colorado, Idaho, Montana, Oregon, South Africa, Utah, Washington, and Wyoming	[142,185,187,300,368,559,701]
*Uromyces psychotriae* Henn.	*Psychotria* sp.	Brazil	[219,575]
*Uromyces pteroclaenae* Lindr.	*Cachryde* sp.	Algeria	[501]
*Uromyces pulchellus* Ellis & Everh.	*Silene douglasii* and *Silene* sp.	California and Washington (USA)	[78,388]
*Uromyces pulvinatus* Kalchbr. & Cooke	*Euphorbia inaequilatera*	South Africa	[702]
*Uromyces punctiformis* Syd. & P. Syd.	*Vigna strobiliphora*	Mexico	[703]
*Uromyces purpureus* Lagerh.	*Liliaceae*	Angola	[704]
*Uromyces pustulatus* Wakef.	*Bauhinia fassoglensis*	Uganda and Kenya	[140,168]
*Uromyces puttemansii* Rangel	*Panicum* spp. and *Setaria* spp.	Argentina, Brazil, Cuba, Honduras, Jamaica, México, Panama, Uruguay, and Venezuela	[126,171,674]
*Uromyces pyriformis* Cooke	*Acorus calamus*	Iowa, Illinois, Japan, Massachusetts, Maine, Minnesota, Mississippi, New York, and Taiwan	[92,155,574,596]
*Uromyces quaggafonteinus* Mennicken & Oberw.	*Ehrharta calycina*	South Africa	[378,429]
*Uromyces quinchamalii* Neger	*Quinchamalium* spp.	Argentina, Bolivia, and Chile	[415,705]
*Uromyces ramacharii* Ravinder & Bagyan.	*Ocimum* sp.	India	[706]
*Uromyces ranunculi-distichophylli* Semadeni	*Ficaria* sp. and *Ranunculus* sp.	Africa, America, China, Canada, Europe, Iran, Japan, and Russia	[106]
*Uromyces rapaneae* Henn.	*Rapanea* sp.	São Paulo	[450]
*Uromyces ratoides* Jørst.	*Cayaponia* spp.	Ecuador	[124,212]
*Uromyces ratus* H.S. Jacks. & Holw.	*Cayaponia* spp.	Brazil	[210,212]
*Uromyces rayssiae* J. Anikster & I. Wahl	*Scilla hyacinthoides*	Israel	[390]
*Uromyces rebeccae* Bruckart, M. Abbasi & Aime	*Suaeda californica*	California	[47]
*Uromyces regius* Vestergr.	*Bauhinia candicans*	Brazil	[216,219]
*Uromyces reichei* Dietel	*Milla bivalvis* and *Triteleia gaudichaudiana*	Chile	[240,415]
*Uromyces reichertii* J. Anikster & I. Wahl	*Scilla hyacinthoides* and *Hordeum bulbosum*	Israel	[81]
*Uromyces renovatus* P. Syd. & Syd.	*Lupinus* sp.	Czech Republic, Greece, Israel, Kenya, Finland, Portugal, and Spain	[81,131,140,144,158,707]
*Uromyces reticulatus* (Thüm.) Bubák	*Allium victorialis*	Portugal and Spain	[158,612]
*Uromyces reynoldsii* Thaung	*Modecca bracteata* and *Trichosanthes* spp.	Myanmar	[212]
*Uromyces rhinacanthi* Cummins	*Rhinacanthus nasutus*	Ghana, West Africa	[218,609]
*Uromyces rhodesicus* Wakef.	*Bauhinia galpinii* and *B. macrantha*	South Africa and Zimbabwe	[138,250]
*Uromyces rhynchosporae* Ellis	*Rhynchospora* spp.	Brazil, Bermuda, California, Cuba, Florida, Georgia, Hawaii, Louisiana, Japan, Massachusetts, Michigan, New Jersey, Puerto Rico, West Indies, and Vermont	[94,166,191,202,306,307,388,412,559]
*Uromyces ribicola* H.S. Jacks. & Holw.	*Ribes albifolium* and *R. andicola*	Bolivia, Brazil, and Colombia	[101,126,227]
*Uromyces rickerianus* Arthur	*Polygonum* spp. and *Rumex* spp.	Colorado, Idaho, Utah, and Wyoming	[187,368]
*Uromyces riloensis* Hinkova	*Doronicum cordifolium*	Bulgaria	[82]
*Uromyces rostratus* Henn.	*Eriosema* sp.	Rio de Janeiro	[331]
*Uromyces rottboelliae* Arthur	*Rottboellia compressa*, *R. exaltata*, and *R. speciosa*	Congo, India, and Philippines	[171,708]
*Uromyces rubidus* Arthur & Holw.	*Andropogon condensatus*	Brazil	[199]
*Uromyces rudbeckiae* Arthur & Holw.	*Rudbeckia* spp. and *Solidago* spp.	Canada, China, Idaho, Florida, Japan, Korea, Iowa, Missouri, Mississippi, Montana, Taiwan, Texas, Wyoming	[94,108,109,185,483,559]
*Uromyces ruelliae* Holw.	*Beloperone californica*, *Beloperone* sp., *Justicia brandegeana*, and *Ruellia* sp.	Arizona, California, Florida, México, and Nevada	[218,262,409,412]
*Uromyces rugosus* Arthur	*Lupinus* sp.	México	[398]
*Uromyces rugulosus* Pat.	*Campylotropis* spp. and *Lespedeza* spp.	China	[96]
*Uromyces ruiz-leali* J.C. Lindq.	*Anarthrophyllum elegans*	Argentina	[415]
*Uromyces rumicis* (Schumach.) G. Winter	*Emex australis*, *Ficaria* spp., *Medicago* spp., *Ranunculus ficaria*, and *Rumex* spp.	Argentina, Armenia, Australia, Brazil, Bulgaria, Chile, Denmark, Finland, Germany, Greece, India, Iran, Italy, Japan, Malawi, New Zealand, Norway, Pakistan, Poland, Portugal, Sicily, South Africa, Spain, Romania, Sweden, Tanzania, Turkey, Uganda, Ukraine, Uzbekistan, and Russia	[75,80,82,128,135,142,158,245,279,313,468,519,534]
*Uromyces rumicis* (Schumach.) G. Winter	*Rumicis* sp.	Australia, Morocco, and Switzerland	[709]
*Uromyces rumicum* (DC.) Fuckel	*Rumicis hydrolapathi*	Egypt and Minnesota	[179,710]
*Uromyces rzedowskii* J.F. Hennen & Cummins	*Ledenbergia macrantha*	México	[292,403]
*Uromyces sabineae* Arthur	*Poitea* spp. and *Sabinea punicea*	Cuba, Dominican Republic, Puerto Rico	[184,486]
*Uromyces saginatus* Syd.	*Urginea altissima*	Namibia, Zimbabwe	[138,250]
*Uromyces sakawensis* Henn.	*Solidago virgaurea*	Japan	[711]
*Uromyces salicorniae* (DC.) de Bary	*Arthrocnemum glaucum* and *Salicornia* spp.	China, Germany, Finland, Poland, Portugal, Romania, and United Kingdom	[105,133,135,195,228,329]
*Uromyces salmeae* Arthur & Holw.	*Galinsoga* sp. and *Salmea scandens*	Costa Rica, Dominican Republic, Guatemala, Puerto Rico, Virgin Islands, and West Indies	[125,184,225,264,281,282,406]
*Uromyces salpichroae* H.S. Jacks. & Holw.	*Salpichroa diffusa* and *Salpichroa* sp.	Bolivia and Ecuador	[210]
*Uromyces salsolae* Rabenh.	*Climacoptera* sp., *Gamanthus gamocarpus*, *Gossypium hirsutum*, *Halocharis hispida*, *Noaea* spp., *Petrosimonia* spp., and *Salsola* spp.	Algeria, China, Cyprus, Finland, Israel, Japan, Mongolia, Morocco, Pakistan, Russia, Romania, Turkey, and Ukraine	[80,81,111,146,198,228,329,435,535]
*Uromyces sasaensis* Gjaerum	*Valeriana kilimandscharica* and *V. volkensii*	Uganda	[317,712]
*Uromyces satarensis* P.B. Chavan & Bakare	*Blainvillea acmella* and *B. latifolia*	China and India	[319,713]
*Uromyces saulensis* Berndt	*Selysia prunifera*	France	[127]
*Uromyces saururi* Henn.	*Saururus chinensis* and *S. loureiroi*	China, Japan, and Taiwan	[96,97,222]
*Uromyces saussureae* P. Karst.	*Saussurea* sp.	Japan, Siberia	[714]
*Uromyces savulescui* Rayss	*Limonium sinuatum* and *Limonium* sp.	The Canary Islands, Greece, and Israel	[81,327]
*Uromyces scaberulus* L. Guo & Y.C. Wang	*Lespedeza bicolor*, *L. cuneate*, *L. cyrtobotrya*, and *L. formosa*	China	[156]
*Uromyces scaevolae* G. Cunn.	*Scaevola albida*, *S. calendulacea*, *S. spinescens*, and *S. radicans*	Australia and New Zealand	[67,313]
*Uromyces schanginiae* Thüm.	*Suaeda* spp.	California and Egypt	[710,715]
*Uromyces schinzianus* Henn.	*Bauhinia fassoglensis*, *B. reticulate*, and *B. thonningii*	Somalia, South Africa, and Uganda	[138,168]
*Uromyces schismi* Jørst.	*Schismus scaberrimus*	South Africa	[411]
*Uromyces schoenanthi* Syd. & P. Syd.	*Andropogon schoenanthus*, *Apluda mutica*, *Cymbopogon schoenanthus*, *Polytrias amaura*, and *P. diversifolia*	India, New Guinea, Pakistan, and the Philippines	[152,201,318]
*Uromyces schweinfurthii* Henn.	*Acacia ehrenbergiana*, *A. flava*, and *A. seyal*	South Yemen and Ethiopia	[287,687]
*Uromyces scillinus* (Durieu & Mont.) Har.	*Scilla autumnalis*	Algeria and Europe	[451]
*Uromyces scirpi-maritimi* Hirats. f. & Yoshin.	*Bolboschoenus maritimus*, *Glaux maritima*, *Scirpus fluviatilis*, and *S. maritimus*	Japan and Russia	[222,297]
*Uromyces scirpinus* Syd.	*Scirpus supinus*	Philippines	[232]
*Uromyces scleranthi* Rostr.	*Minuartia* spp. and *Scleranthus* spp.	Australia, Bulgaria, Denmark, Finland, Norway, Sweden, Turkey, and Ukraine	[80,107,128,280,329,422]
*Uromyces scleriae* Henn.	*Scleria* spp.	Barbados, Brazil, Colombia, Cuba, Dominica, Grenada, Guyana, Nigeria, Rio de Janeiro, Saint Lucia, Puerto Rico, Venezuela, and West Indies	[122,184,219,264,323,431]
*Uromyces sclerochloae* Tranzschel	*Sclerochloa dura*	Central Asia (Turkmenistan and Iran)	[161,383]
*Uromyces scleropoae* Baudyš & Picb.	*Scleropoa ridiga*	Croatia	[451]
*Uromyces scrophulariae* (DC.) Fuckel	*Scrophularia auriculata*	Carmarthenshire and Wales	[716]
*Uromyces scutellatus* (Schrank) Niessl	*Euphorbia* spp.	Bulgaria, Chile, Germany, Iran, Iraq, Poland, Romania, Russia, Serbia, Spain, Turkey, Ukraine, and Uzbekistan	[43,80,82,105,111,146,179,196,228,236,347,386]
*Uromyces secamones* Wakef.	*Secamone platystigma*	Uganda	[168]
*Uromyces sedi* Gäum.	*Sedum anacampseros*	France	[717]
*Uromyces seditiosus* F. Kern	*Aristida* spp. and *Plantago* spp.	Alabama, Arkansas, California, Colorado, Illinois, Indiana, Kansas, Iowa, Missouri, Nebraska, Nebraska, New York, Oklahoma, Texas, Wisconsin, and Virginia	[537,559,718]
*Uromyces seligeri* Tranzschel & Erem.	*Lathyrus grandiflorus* and *L. sylvestris*	Greece and Russia	[220,382,421]
*Uromyces sellierae* G. Cunn.	*Selliera radicans*	New Zealand	[67]
*Uromyces semnanensis* Gjaerum	*Astragalus fridae*	Iran	[276]
*Uromyces senecionicola* Arthur	*Cacalia* sp., *Senecio roldana*, and *Senecio* sp.	México	[292,398]
*Uromyces senecionis-gigantis* Gjaerum	*Senecio gigas*	Ethiopia	[719]
*Uromyces senorensis* J.F. Hennen & Cummins	*Compositae*	Mexico	[703,720]
*Uromyces sepultus* Mains	*Setaria tenax*	America, México	[721]
*Uromyces seseli-graminis* E. Fisch.	*Arrhenatherum elatius*, *Melica ciliate*, and *Poae* sp.	Europe	[106]
*Uromyces seselis* Sousa da Câmara	*Seseli tortuosum*	Portugal	[722]
*Uromyces sesseae* Lagerh.	*Sessea* sp.	Ecuador	[325]
*Uromyces setariae-italicae* Yoshino	*Brachiaria* spp., *Chaetochloa* spp., *Eriochloa* spp., *Lasiacis* spp., *Setaria* spp., and *Urochloa* spp.	Worldwide	[294]
*Uromyces shahrudensis* Petr.	*Onobrychis* sp.	Iran	[723]
*Uromyces shearianus* Arthur		Arizona, California, Colorado, México, Utah, and Wyoming	[114,292,412,559]
*Uromyces shikokianus* Kusano	*Cladrastis platycarpa* and *C. shikokiana*	Japan	[222]
*Uromyces sii-latifolii* P. Karst.	*Sium latifolium*	Britain and Russia	[714]
*Uromyces silenes* (Schltdl.) Fuckel	*Arenaria glabrescens*, *Arenaria* sp., and *Silene* spp.	California, Colorado, Iowa, Kansas, Montana, Pennsylvania, Portugal, Turkey, Utah, and Washington (USA)	[80,114,454,559]
*Uromyces silenes-chloraefoliae* Vienn.-Bourg.	*Silene chlorifolia*	Iran	[320]
*Uromyces silenes-ponticae* Const.	*Silene* spp.	Bugaria, Iraq, Libya, Romania, Portugal, and Turkey	[80,82,196,228,532]
*Uromyces silksvleyensis* Mennicken & Oberw.	*Bartholina burmanniana*	Western Cape Province	[378]
*Uromyces silphii* (Syd. & P. Syd.) Arthur	*Aster macrophyllus*, *Helianthus* spp., *Heliopsis helianthoides*, *Juncus* spp., and *Silphium* spp.	Argentina, Canada, Czech Republic, Chile, Florida, Georgia, Idaho, Illinois, Iowa, México, Minnesota, Missouri, New York, Oklahoma, Oregon, Pennsylvania, Poland, Texas, Uruguay, Washington, Wisconsin, and Vermont	[185,187,191,292,415,559]
*Uromyces simulans* Peck	*Vilfa* sp.	Colorado	[724]
*Uromyces siphocampyli-gigantei* Berndt	*Siphocampylus giganteus*	Ecuador	[725]
*Uromyces sisyrinchiicola* Speg.	*Sisyrinchium iridifolium*	Chile	[258]
*Uromyces skottsbergii* Jørst.	*Enargea marginata*	Argentina and the Falkland Islands	[726,727]
*Uromyces smilacis* Mayor	*Smilax* sp.	Colombia	[226,227]
*Uromyces snowdeniae* Cummins	*Snowdenia scabra*	Kenya	[140]
*Uromyces socius* Arthur & Holw.	*Loranthus* spp. and *Struthanthus* spp.	Guatemala and México	[15,281]
*Uromyces solani* Dietel & Holw.	*Solanum appendiculatum*	Colombia, Guatemala, and México	[227,281,456,479]
*Uromyces solariae* Dietel	*Solaria miersioides*	Chile	[240]
*Uromyces solidaginis* (Sommerf.) Fuckel	*Solidago* spp.	Colorado, Finland, Idaho, Japan, Montana, Oregon, Poland, Russia, Washington, and Wyoming	[92,329,454,559]
*Uromyces solidaginis-caricis* Arthur	*Carex varia*	Indiana	[728]
*Uromyces solidus* Berk. & M.A. Curtis	*Desmodium strictum*	North Carolina	[657]
*Uromyces sommerfeltii* Hyl., Jørst. & Nannf.	*Solidago* spp.	China, Canada, Germany, Finland, Idaho, Japan, Russia, Mississippi, Montana, Nepal, Norway, Oregon, Poland, Russia, Sweden, and Turkey	[80,94,129,130,131,135,185,187,436,475,567]
*Uromyces sonorensis* J.F. Hennen & Cummins	*Merremia palmeri*	México	[729]
*Uromyces sophorae* Peck	*Sophora alopecuroides*, *S. sericea*, and *S. mollis*	Pakistan and México	[563,730]
*Uromyces sophorae-flavescentis* Kusano	*Sophora alopecuroides*, *S. angustifolia*, *S. flavescens*, *S. jaubertii*, and *S. japonicum*	China, Japan, Korea, Turkey, Russia, and Uzbekistan	[96,146,297,483]
*Uromyces sophorae-japonicae* Dietel	*Sophora japonica*	Japan	[92,731]
*Uromyces sophorae-vicifoliae* F.L. Tai	*Sophora viciifolia*	China	[732]
*Uromyces sparaxidis* Syd. & P. Syd.	*Sparaxis lineata*, *S. tricolor*, and *Sparaxis* sp.	South Africa	[142]
*Uromyces sparganii* Cooke & Peck	*Acorus calamus*, *Hypericum virginicum*, and *Sparganium eurycarpum*	Canada, Iowa, Indiana, India, Michigan, Mississippi, New York, Nebraska, and Wisconsin	[152,185,475]
*Uromyces sparsus* (Kunze & J.C. Schmidt) Lév.	*Arenaria marina*, *Spergularia* spp. and *Stellaria patens*	Bulgaria, Denmark, Finland, Germany, Nepal, Norway, Romania, and Ukraine	[82,111,128,135,179,188,228,329]
*Uromyces spartii-juncei* P. Syd. & Syd.	*Spartium junceum*	France, Greece, Portugal, Spain, Switzerland, and Yugoslavia	[220]
*Uromyces speciosus* Holw.	*Frasera macrophylla*	Colorado and New México	[559,733]
*Uromyces spegazzinii* (De Toni) Arthur	*Commelina angustifolia*, *C. elegans*, *C. erecta*, *C. nudiflora*, and *C. virginica*	Florida, Taiwan, Texas, and the Virgin Islands	[299,447]
*Uromyces spermacoces* (Schwein.) Thüm.	*Diodia teres*, *D. virginiana*, *Diodia* sp., and *Spermacoce tenuior*	Brazil, Carolina, Florida, Georgia, Indiana, Missouri, Mississippi, Oklahoma, Pennsylvania, and Texas	[202,203,262,455,584]
*Uromyces sphaericus* H.S. Jacks. & Holw.	*Perymenium ecuadoricum*	Ecuador	[210,219]
*Uromyces sphaerocarpus* Syd. & P. Syd.	*Indigofera potaninii*, *I. pseudotinctoria*, *I. tinctoria*, and *Indigofera* sp.	China and Japan	[96,734]
*Uromyces sphaerophysae* Pospelov ex Nevod.	*Swainsona salsula*	China	[198]
*Uromyces splendens* A. Blytt	*Astragalus oroboides*	Norway	[735]
*Uromyces sporoboli* Ellis & Everh.	*Allium* spp., *Sporobolus* spp.	Bolivia, Chile, Indiana, Kansas, Iowa, Missouri, Nebraska, Puerto Rico, Texas, and Wisconsin	[199,386,559,635]
*Uromyces sporobolicola* J.C. Lindq.	*Sporobolus marginatus*, *S. pyramidalis*, and *S. pyramidatus*	Argentina, India, Pakistan, and México	[171,415,488,736]
*Uromyces sporoboloides* Cummins	*Sporobolus berteroanus*	Ecuador	[737]
*Uromyces spragueae* Harkn.	*Calyptridium umbellatum*, *C. umbellatum* var. *caudiciferum*, *Spraguea umbellate*, and *Spraguea* sp.	California, Oregon, and Wyoming	[186,559,658]
*Uromyces standleyanus* Arthur	*Gaudichaudia schiedeana*	El Salvador	[301]
*Uromyces statices* Berk. & M.A. Curtis	*Statice* sp.	California	[598]
*Uromyces statices-mucronatae* Malençon	*Statice mucronata*	Morocco	[279]
*Uromyces statices-sinensis* Liou & Y.C. Wang	*Statice sinensis*	China	[543]
*Uromyces steironematis* Arthur	*Spartina michauxiana*	Nebraska	[738]
*Uromyces stellariae* Syd. & P. Syd.	*Stellaria kotschyana*	Iran	[739]
*Uromyces stellariae-saxatilis* L. Guo & Y.C. Wang	*Stellaria media*, *S. saxatilis*, *S. vestita*, and *Stellaria* sp.	China	[96]
*Uromyces stenorrhynchi* Henn.	*Stenorrhynchus* sp.	Peru	[740]
*Uromyces stipinus* Tranzschel & Erem.	*Stipa rubens*	Russia	[106]
*Uromyces strauchii* Doidge	*Clutia daphnoides*	Southern Africa	[138]
*Uromyces striatellus* Tranzschel	*Euphorbia esula*, *E. leptocaula*, *E. sieboldiana*, and *Euphorbia* sp.	China, Iran, Japan, Ukraine, and Russia	[94,108,109,157,297]
*Uromyces striatus* J. Schröt.	*Argyrolobium* spp., *Cicer arietinum*, *Ervum lens*, *Euphorbia* spp., *Hosackia* sp., *Hymenocarpos circinnatus*, *Lathyrus odoratus*, *Lens culinaris*, *Lotus* spp., *Manihot esculenta*, *Medicago lupulina*, *Melilotus* spp., *Pisum* spp., *Trifolium* spp., and *Vicia* spp.	Worldwide	[104,165,220,222,225]
*Uromyces striolatus* Tranzschel	*Euphorbia aff-esula*, *E. boissieriana*, *E. cyparissias*, *E. esula*, and *Euphorbia* sp.	Bulgaria, China, Iran, and Pakistan	[82,108,157,317,356]
*Uromyces strobilanthis* Barclay	*Pteracanthus urticifolius*,*Strobilanthes alata*, *S. dalhousieana*, and *Strobilanthes* sp.	India, Pakistan, Taiwan, and the Philippines	[218,563,565]
*Uromyces strumariae* A.R. Wood	*Strumaria gemmata*	Western Cape Province	[741]
*Uromyces struthanthi* Perd.-Sánch.	*Struthanthus* sp.	Panama	[15]
*Uromyces stylochaetonis* Doidge	*Stylochaeton natalense*	KwaZulu-Natal	[249]
*Uromyces sublevis* Tranzschel	*Euphorbia baetica*, *E. petrophila*, and *E. tictoria*	Lebanon, Portugal, and Ukraine	[157,158,422,449]
*Uromyces substriatus* Syd. & P. Syd.	*Lupinus argenteus*	Montana	[79]
*Uromyces suksdorfii* Dietel & Holw.	*Lychnis drummondii*, *Lychnis* sp., *Silene oregano*, *S. pacifica*, *S. scouleri*, and *Silene* sp.	Arizona, California, Idaho, Oregon, Utah, México, and Washington	[114,116,187,454,559]
*Uromyces superfixus* Vestergr.	*Bauhinia mirandina*, *B. mollis*, and *Bauhinia* sp.	Argentina, Brazil, Bolivia, and Venezuela	[216,394,431]
*Uromyces superfluus* P. Syd. & Syd.	*Panicum antidotale* and *P. depauperatum*	America, India, and Pakistan	[144,171,294]
*Uromyces superstomatalis* Berndt	*Cayaponia rigida*	France	[127]
*Uromyces suzukii* Sawada ex Hirats. f.	*Sigesbeckia orientalis*	China, Japan, and Taiwan	[94,108,319]
*Uromyces symaethidis* W. Schneid.	*Simethis bicolor*	Italy	[742]
*Uromyces tairae* Hirats. f.	*Messerschmidia argentea*	Japan	[743]
*Uromyces tarapotensis* Henn.	*Camptosema* sp.	Peru	[575]
*Uromyces teheranicus* Petr.	*Trifolium retense*	Iran	[744]
*Uromyces tehuelches* Speg.	*Alstroemeria patagonica*	Argentina	[167,592]
*Uromyces tener* J. Schröt.	*Manettia gracilis*	Brazil	[219,331]
*Uromyces tenuicutis* McAlpine	*Parietaria judaica* and *Sporobolus* spp.	Australia, China, Fiji, Ghana, Guyana, Grenada, India, Japan, Jamaica, Kenya, Malawi, México, Malaysia, Nigeria, Pakistan, Philippines, Puerto Rico, South Africa, Sierra, Taiwan, Uganda, Virgin Island, West Indies, and Zimbabwe	[97,171,172,184,243,244,280,615]
*Uromyces tenuistipes* Dietel & Holw.	*Desmodium* spp. and *Meibomia* sp.	Bolivia, Colombia, Cuba, and México	[172,479,597,745]
*Uromyces teodorescui* Rayss	*Onobrychis crista-galli*	Israel and Palestine	[81]
*Uromyces tepicensis* J.F. Hennen & Cummins	*Loeselia amplectens*	México	[292]
*Uromyces tessariae* (Speg.) J.C. Lindq.	*Tessaria absinthioides*	Spain	[746]
*Uromyces thapsi* Opiz ex Bubák	*Verbascum* spp.	Greece, Romania, Spain, Turkey, Ukraine, and Uzbekistan	[111,146,158,228,421]
*Uromyces thellungi* Maire	*Rumex roseus*, *R. simpliciflorus*, and *R. vesicarius*	Canary Islands, Iran, Israel, and Pakistan	[472]
*Uromyces thelymitrae* McAlpine	*Thelymitra antennifera* and *T. flexuosa*	Australia	[81,280,317,356]
*Uromyces thermopsidicola* Shimab.	*Thermopsis chinensis*	Japan	[222]
*Uromyces tinctoriicola* Magnus	*Euphorbia* spp.	Armenia, Germany, Iraq, Morocco, Spain, Turkey, Romania, and Ukraine	[80,196,228,422,519]
*Uromyces tingitanus* Henn.	*Rumex* spp.	The Canary Islands, Libya, and Morocco	[197,279]
*Uromyces tolerandus* H.S. Jacks. & Holw.	*Manihot esculenta* and *Manihot* sp.	Brazil	[219]
*Uromyces tomentellus* Cooke	*Leguminosae* sp.	California	[747]
*Uromyces tordillensis* Speg.	*Euphorbia serpens* and *E. ovalifolia*	Argentina, Chile, Córdoba, and Uruguay	[371,386]
*Uromyces tosensis* Henn.	*Commelina communis*	Japan	[711]
*Uromyces tournefortiae* Henn.	*Tournefortia* sp.	Brazil	[600]
*Uromyces tragi* Wakef. & Hansf.	*Tragus berteronianus*	Malawi and Uganda	[168,273]
*Uromyces transcaspicus* Petr.	*Astragalus angustidens*	Turkmenistan	[748]
*Uromyces transversalis* (Thüm.) G. Winter	*Crocosmia* spp., *Freesia refracta*, *Gladiolus* spp., *Tritonia* spp., and *Watsonia* spp.	Argentina, Australia, Brazil, California, Cuba, England, Florida, France, Malwai, Martinique, Mauritius, México, New Zealand, South Africa, Tanzania, Uganda, Venezuela, Zambia, and Zimbabwe	[69,142,209,250,273,487,530,650]
*Uromyces tranzschelii* Syd. & P. Syd.	*Euphorbia crenulata*, *E. lucida*, *E. montana*, *E. palmeri*, *E. robusta*, and *E. robusta*	Azirona, Colorado, Montana, Oregon, Utah, and Wyoming	[103,157,187,292,368,412]
*Uromyces traucoensis* Monteal. & Oehrens	*Selliera radicans*	Chile	[749]
*Uromyces triandrae* T.S. Ramakr. & Sriniv.	*Themeda triandra*	India	[669]
*Uromyces trichoclines* Henn.	*Trichocline polymorpha*	Brazil	[219,323]
*Uromyces tricholenae* Gonz. Frag. & Cif.	*Tricholaena rosea*	Dominican Republic	[750]
*Uromyces trichoneurae* Doidge	*Astrebla elymoides*, *A. lappacea*, *A. pectinate*, *A. squarrosa*, *Trichoneura grandiglumis*, and *T. lisboae*	Australia, India, South Africa	[169,488,751]
*Uromyces tricorynes* McAlpine	*Tricoryne elatior*	Australia	[245,267]
*Uromyces trifolii-megalanthi* (Dietel & Neger) H.S. Jacks. & Holw.	*Trifolium peruvianum* and *Trifolium* sp.	Brazil, Chile, and Peru	[101,126]
*Uromyces trifolii-purpurei* Const.	*Trifolium campestre*, *T. eriosphaerum*, *T. purpureum*, *T. scabrum*, *T. stellatum*, and *Trifolium* sp.	Bulgaria, Greece, France, Israel, Italy, Russia, Romania, and Spain	[81,82,126,228,327]
*Uromyces trifolii-repentis* Liro	*Trifolium* spp.	Worldwide	[225,361]
*Uromyces trigonellae* Pass.	*Trigonella* spp.	Bulgaria, Israel, Romania, and Turkey	[80,81,82,197,228,357]
*Uromyces trigonellae-occultae* Henn.	*Trigonella occulta*	Egypt	[752]
*Uromyces tripogonicola* Payak & Thirum.	*Tripogon lisboae*	India	[753]
*Uromyces tripogonis-sinensis* Y.C. Wang	*Tripogon chinensi* and *T. lisboae*	China and India	[106,108,109]
*Uromyces tripsaci* F. Kern & Thurst.	*Tripsacum dactyloides*	Ecuador and Venezuela	[431,754]
*Uromyces triquetrus* Cooke	*Ascyrum hypericoides*, *Hypericum* spp., and *Triadenum japonicum*	Argentina, Brazil, California, Canada, China, Colombia, Indiana, Indonesia, Iowa, Maine, México, Missouri, Mississippi, Taiwan, Oregon, Japan, and Wisconsin	[96,97,187,219,252,255,292,412,415,449,475]
*Uromyces triseti* Katajev	*Trisetum cavanillesii*	Turkmenistan	[106]
*Uromyces triteleiae* Dietel & Neger	*Brodiaea porrifolia*, *Leucocoryne alliacea*, and *Triteleia porrifolia*	Chile	[162,166,240,415]
*Uromyces trollii-caroli* Ulbr.	*Trifolium* sp.	Germany and India	[34,755]
*Uromyces trollipii* Kalchbr. & MacOwan	*Roepera foetida* and *Zygophyllum foetidum*	South Africa	[138,385]
*Uromyces tropaeoli* Ranoj.	*Tropaeolum major*	Yugoslavia	[212]
*Uromyces truncatulus* Trotter	*Geranium versicolor*	Greece	[421]
*Uromyces truncicola* Henn. & Shirai	*Sophora japonica* and *Sophora* sp.	China, Japan, and Korea	[468,483,734]
*Uromyces tuberculatus* Fuckel	*Euphorbia* spp.	The Balearic Islands, Canary Islands, China, Finland, Germany, Iran, Malaysia, Romania, Pakistan, Portugal, Spain, and Uzbekistan	[105,129,130,228,317,318,329,356]
*Uromyces tulipae* Dietel	*Tulipa edulis*	Japan	[756]
*Uromyces tungurahuensis* Syd.	*Aspilia lanceolata*	Ecuador	[757]
*Uromyces turcomanicus* Katajev	*Muscari leucostomum*, *Hordeum bulbosum*, *H. marinum*, and *H. spontaneum*	Iraq, Jordan, Libya, Russia, and Turkmenistan	[126,243,244]
*Uromyces tylosemae* Gjaerum	*Tylosema fassoglensis*	Uganda and Sudan	[428]
*Uromyces uleanus* Dietel	*Euphorbia* sp.	Brazil	[143,219]
*Uromyces umiamensis* Berndt & Baiswar	*Cucumis* sp. and *Momordica cochinchinensis*	India	[127,212,234]
*Uromyces undulatoparietis* B. Li	*Ligularia hookeri*	China	[108,109]
*Uromyces undulatus* Tranzschel	*Euphorbia condylocarpa* and *Euphorbia* sp.	Armenia and Iran	[157,318]
*Uromyces unioniensis* Viégas	*Desmodium* sp. and *Meibomia* sp.	Brazil	[394,640]
*Uromyces unitus* Peck	*Calandrinia leeana*	Washington (USA)	[758]
*Uromyces urariae* B. Li	*Uraria lagopodioides*	China	[759]
*Uromyces urbanianus* Henn.	*Oryctanthus spicatus*	Argentina, Brazil, Colombia, Guatemala, Honduras, México, and Trinidad and Tobago	[15,227,473]
*Uromyces urgineae* M.S. Patil	*Urginea indica*	India	[397]
*Uromyces ushuwaiensis* Speg.	*Impatiens hochstetteri*	Ethiopia	[258,760]
*Uromyces ustalis* Tranzschel	*Ranunculus repens*	Siberia	[761]
*Uromyces usterianus* Dietel	*Myrtaceae*	Colombia and São Paulo	[762]
*Uromyces valens* F. Kern	*Carex aestivalis*, *C. lupulina*, and *C. utriculata*	Indiana, North Carolina, and Wisconsin	[763,764,765]
*Uromyces valerianae* (Schumach.) Fuckel	*Valerianella* spp.	Bulgaria, China, Czech Republic, Czechoslovakia, Denmark, Finland, France, Germany, Greece, Japan, Mongolia, Norway, Poland, Romania, Scotland, Spain, South Africa, Sweden, Switzerland, Taiwan, Turkey, and Ukraine	[80,82,90,94,128,132,133,135,138,228,283,421,440]
*Uromyces valerianae-microphyllae* Berndt	*Valeriana microphylla*	Ecuador	[766]
*Uromyces valerianae-wallichii* (Dietel) Arthur & Cummins	*Valeriana* spp.	China, Japan, Taiwan, India, and Pakistan	[87,88,94,108,109,356]
*Uromyces valesiacus* E. Fisch.	*Vicia ervilia* and *V. onobrychioides*	Albania, Bulgaria, France, Greece, Romania, Switzerland, Turkey, and Yugoslavia	[82,126,220]
*Uromyces vanderystii* Henn.	*Teramnus labialis*	The Democratic Republic of the Congo	[610]
*Uromyces vankyorum* Berndt	*Atriplex lampa*	Argentina	[282]
*Uromyces venustus* Dietel & Holw.	*Cestrum nitidum*	México	[292,375]
*Uromyces veratri* (DC.) J. Schröt.	*Adenostyles* spp., *Homogyne alpine*, *Ligularia* spp., and *Veratrum* spp.	Austria, Bulgaria, China, Germany, Japan, Korea, Poland, Romania, Russia, Spain, Switzerland, and Turkey	[82,135,158,222,228,231,283,402]
*Uromyces verbasci* Niessl	*Verbascum* spp.	Bulgaria, Germany, Poland, and Ukraine	[82,105,135]
*Uromyces verrucosae-craccae* Mayor	*Vicia cracca*	France and Switzerland	[126,220]
*Uromyces verruculosus* J. Schröt.	*Arenaria serpyllifolia*, *Cucubalus baccifer*, *Dianthus armeria*, *Lychnis* spp., *Melandrium* spp., and *Silene* spp.	Armenia, Bulgaria, Czech Republic, Denmark, Greece, Germany, Indiana, Iran, Michigan, New York, Norway, Poland, Romania, Sweden, Texas, Turkey, Ukraine, and Wisconsin	[105,107,135,228,317,422,519]
*Uromyces verus* H.S. Jacks. & Holw.	*Bauhinia rufa*	Brazil	[101]
*Uromyces vesicatorius* (Bubák) Nattrass	*Lens esculenta* and *L. leontopetalum*	Cyprus, Iraq, Israel, and Turkey	[80,81,196,599]
*Uromyces vesiculosus* G. Winter	*Zygophyllum* spp.	Australia	[245]
*Uromyces vestergrenii* P. Syd. & Syd.	*Bauhinia tomentosa*	India and Sri Lanka	[165]
*Uromyces viciae-craccae* Const.	*Ervum lens*, *Euphorbia* sp., *Lathyrus aphaca*, *Lens* spp., and *Vicia* spp.	Armenia, Austria, Azerbaijan, Bulgaria, China, Czech Republic, Finland, France, Germany, Georgia, Greece, Hungary, Iran, Itlay, Kazakhstan, Poland, Romania, Russia, Siberia, Switzerland, Ukraine, and Yugoslavia	[82,135,220,767,768]
*Uromyces viciae-fabae* (Pers.) J. Schröt.	*Vicia* spp., *Lathyrus* spp., and *Pisum* spp.	Worldwide	[71,222,236,238,769]
Uromyces *viciae-unijugae* S. Ito	*Vicia unijuga*	Japan	[92]
*Uromyces vicinus* H.S. Jacks. & Holw.	*Ipomoea* sp.	Brazil	[219]
*Uromyces vicosensis* R.T. Almeida	*Bauhinia* sp.	Brazil	[219]
*Uromyces viegasii* R.T. Almeida	*Bauhinia forficata* and *Bauhinia* sp.	Brazil	[219,394]
*Uromyces viennot-bourginii* J. Anikster & I. Wahl	*Bellevalia eigii* and *Hordeum spontaneum*	Israel	[81]
*Uromyces vignae* Barclay	*Desmodium* spp., *Dipogon* spp., *Lablab* sp., *Phaseolus* sp., *Sphenostylis* sp., and *Vigna vexillata*	Worldwide	[71,222,225]
*Uromyces vignae-luteolae* Henn.	*Vigna luteola*	Congo	[610]
*Uromyces vignae-sinensis* Miura	*Vigna sinensis*	China and Japan	[89,96]
*Uromyces visci* T. Majewski & K.A. Nowak	*Viscum congolense*	Rwanda	[551]
*Uromyces volkartii* Gäum. & Terrier	*Trisetum flavescens*	Switzerland	[770]
*Uromyces vossiae* Barclay	*Phacelurus speciosus*, *Rottboellia* sp., and *Vossia speciosa*	India and Pakistan	[106,294,771]
*Uromyces vulpiae* Sousa da Câmara	*Vulpia broteri*	Portugal	[772]
*Uromyces waipoua* McNabb	*Hypericum gramineum* and *H. japonicum*	New Zealand	[313]
*Uromyces wartoensis* Petr.	*Astragalus wartoensis*	Armenia and Turkey	[220,276]
*Uromyces wedeliae* Henn.	*Wedelia bicolour*, *W. biflora*, *W. chinensis*, *W. menotriche*, and *W. prostrata*	China, Eritrea, Fiji, Japan, Micronesia, Philippines, Taiwan, and Tonga	[94,97,108,109,287,565,654]
*Uromyces wedeliae-biflorae* Boedijn	*Wedelia biflora*	Indonesia	[309]
*Uromyces wellingtonicus* T.S. Ramakr. & K. Ramakr.	*Sporobolus indicus*	India	[629]
*Uromyces wiehei* Cummins	*Thalictrum rhynchocarpum*	Kenya, Malawi, and Uganda	[318,609,712]
*Uromyces wolfii* Cummins	*Borreria laevis*	Venezuela	[609]
*Uromyces wulffiae* Henn.	*Wulffia baccata*, *W. maculata*, *W. scandens*, *W. stenoglossa*, and *Wulffia* sp.	Brazil	[219,394,575]
*Uromyces wulffiae-stenoglossae* Dietel	*Wulffia baccata*, *W. maculata*, *W. stenoglossa*, and *Wulffia* sp.	Brazil, French Guiana, Guyana, Trinidad and Tobago, Venezuela, and West Indies	[120,219,264,431,557]
*Uromyces yakushimensis* Hirats. f. & Katsuki	*Trichosanthes bracteata*, *T. multiloba*, and *T. palmata*	Japan and Myanmar	[94,127,212]
*Uromyces yoshinagae* Henn.	*Pisum sativum*	Honshu	[360]
*Uromyces yurimaguasensis* Henn.	*Clitoria guianensis* and *Clitoria* sp.	Belize and Peru	[172,575,721]
*Uromyces zeyheri* Bubák	*Ixia scillaris*, *I. scillaris*, and *Tritonia pallida*	South Africa	[123,138,165]
*Uromyces zigadeni* Peck	*Zigadenus paniculatus*	Utah	[328]
*Uromyces zizaniae-latifoliae* Sawada	*Zizania latifolia*	Taiwan	[773]

**Table 4:** World distribution of endemic/native *Uromyces* species.

*Uromyces* Species	Host	Country	Reference
*Uromyces actinostemonis*	*Actinostemon concolor*	Brazil	[103]
*Uromyces acutatus*	*Gageabohemica* and *G*. *villosa*	Germany	[105]
*Uromyces aegopogonis*	*Aegopogon cenchroides*, *A*. *geminiflorus*, *A*. *gracilis*, and *A*. *tenellus*	México	[106]
*Uromyces aeluropodinus*	*Aeluropus littoralis*	Ukraine	[111]
*Uromyces agnatus*	*Jatropha stimulosa*	Florida	[112]
*Uromyces agropyri*	*Agropyron* sp.	India	[126]
*Uromyces aimeae*	*Cucurbitaceae*	Ecuador	[127]
*Uromyces albiziae*	*Albizia procera*	Indinesia	[136]
*Uromyces allii-monanthi*	*Allium monanthum*	Japan	[147]
*Uromyces allii-sibirici*	*Allium sibiricum*	Norway	[149]
*Uromyces allii-victorialis*	*Allium fistulosum*, *A*. *macrostemon*, and *A*. *victorialis*	China	[90]
*Uromyces alsinis*	*Minuartia hamata* and *M*. *meyeri*	Turkey	[80]
*Uromyces alyxiae*	*Alyxia oliviformis*	Hawaii	[176]
*Uromyces amphilophis-insculptae*	*Amphilophis insculpta*	India	[194]
*Uromyces anabasis*	*Anabasis aphylla*	China	[195]
*Uromyces anomathecae*	*Anomatheca cruenta*	South Africa	[169]
*Uromyces anotidis*	*Anotis richardiana*	Sri Lanka	[214]
*Uromyces antioquiensis*	*Rhynchospora polyphylla* and *R*. *nervosa*	Colombia	[119,226,227]
*Uromyces antipae*	*Rosa lutea*	Romania	[228]
*Uromyces aphelandrae*	*Aphelandra pectinata*	Costa Rica	[218]
*Uromyces aquiriensis*	*Cucurbitaceae*	Israel	[127]
*Uromyces araucanus*	*Senecio otites*	Chile	[240]
*Uromyces arenariae-grandiflorae*	*Arenaria saponarioides*	Turkey	[80]
*Uromyces argutus*	*Spartina alterniflora* and *S*. *glabra*	Florida	[106]
*Uromyces asperulae*	*Asperula conferta* and *A*. *oligantha*	Australia	[268]
*Uromyces aspiliellus*	*Aspilia latifolia*	Ivory Coast	[270]
*Uromyces astragali-alopecuri*	*Astragalus alopecurus*	Turkey	[276]
*Uromyces astragali-atropilosuli*	*Astragalus atropilosulus*	Kenya	[276]
*Uromyces astragali-pseudoutrigeris*	*Astragalus pseudoutriger*	Turkey	[276]
*Uromyces atlanticus*	*Hippocrepis scabra*	Morocco	[279]
*Uromyces azorellae*	*Pozoa trifoliate* and *Schizeilema trifoliolatum*	New Zealand	[284]
*Uromyces babianae*	*Babiana disticha*	Western Cape Province	[285]
*Uromyces baccarinii*	*Wedelia* sp.	Eritrea	[287]
*Uromyces badius*	*Haemanthus coccineus*, *H*. *pumilio*, *H*. *rotundifolius*, and *H. sanguineus*	South Africa	[167]
*Uromyces bahiensis*	On leaves of *Loranthaceae*	Panama	[15]
*Uromyces bauhiniicola*	*Bauhinia chlorantha* and *B*. *pringlei*	México	[398]
*Uromyces beckmanniae*	*Beckmannia eruciformis* and *B*. *syzigachne*	Oregon	[295]
*Uromyces belemensis*	*Ormosia nobilis*	Brazil	[219]
*Uromyces bermudianus*	*Cyperus paniculatus*	Bermuda	[224]
*Uromyces bethelii*	*Silene verecunda*	California	[301]
*Uromyces bisbyi*	*Eriogonum parvifolium*	California	[314]
*Uromyces boissierae*	*Boissiera pumilio*	Iran	[320]
*Uromyces bolusii*	*Aspalathus pachyloba*	South Africa	[322]
*Uromyces bomareae*	*Bomarea* sp.	Brazil	[167]
*Uromyces bonaerensis*	*Gomphrena elegans*	Buenos Aires	[324]
*Uromyces bonae-spei*	*Tritonia scillaris* and *Acidanthera pallida*	Southern Africa	[325]
*Uromyces bonaveriae*	*Bonaveria securidaca* and *Securigera securidaca*	Greece	[327]
*Uromyces borreriae*	*Borreria verticillata*	Rio de Janeiro	[331]
*Uromyces bosseri*	*Trochomeriopsis diversifolia*	Madagascar	[127]
*Uromyces bothriochloae-intermediae*	*Bothriochloa intermedia*	China	[108,109]
*Uromyces bradburyae*	*Bradburya pubescens*, *B*. *virginiana*, *Centrosema pubescens*, and *C*. *virginianum*	Brazil	[219]
*Uromyces brizae*	*Briza media*	France	[339]
*Uromyces bromicola*	*Bromus coloratus* and *B*. *lithobius*	Chile	[199]
*Uromyces buforrestiae*	*Buforrestia imperforata*	Ghana	[341]
*Uromyces bulbinicola*	*Bulbine bulbosa*	Australia	[249]
*Uromyces bunsteri*	*Sisyrinchium cuspidatum* and *S*. *graminifolium*	Chile	[166]
*Uromyces calopogonii*	*Calopogonium galactioides*	Guatemala	[172]
*Uromyces calotheus*	*Urginea* sp.	Sierra Leone	[351]
*Uromyces calycotomes*	*Calycotome spinosa*	France	[220]
*Uromyces caricis-brunneae*	*Carex brunnea*	Japan	[94]
*Uromyces caricis-schmidtii*	*Carex schmidtii*	Khabarovsk	[297]
*Uromyces cassiae-mimosoidis*	*Cassia mimosoides* and *Chamaecrista mimosoides*	South Africa	[172]
*Uromyces cearensis*	*Ipomoea* sp.	Brazil	[373]
*Uromyces cedrelae*	*Toona serrata*	Indonesia	[177]
*Uromyces celtidis*	*Celtis* sp.	Brazil	[102]
*Uromyces cenisiae*	*Ononis cenisia*	France	[342]
*Uromyces chaetobromi*	*Chaetobromus dregeanus* and *C*. *schraderi*	South Africa	[378]
*Uromyces chilensis*	*Lathyrus magellanicus* and *L*. *multiceps*	Chile	[386]
*Uromyces chiovendae*	*Cissus* sp.	Somalia	[287]
*Uromyces chorizanthis*	*Chorizanthe pungens*	California	[388]
*Uromyces christensenii*	*Muscari parviflorum* and *Hordeum bulbosum*	Israel	[390]
*Uromyces chubutensis*	*Poa chubutensis*	Chubut	[391]
*Uromyces ciceris-soongaricae*	*Cicer songaricum*	Pakistan	[393]
*Uromyces circinalis*	*Scilla prasina*	South Africa	[138]
*Uromyces cladomanes*	*Vitis* sp.	Somalia	[287]
*Uromyces cladrastidis*	*Cladrastis shikokiana*	Japan	[92]
*Uromyces clignyioides*	*Monocymbium ceresiiforme*	Zimbabwe	[243]
*Uromyces clitoriae*	*Clitoria mexicana*	México	[172]
*Uromyces clivalis*	*Argyrolobium flaccidum*	India	[357]
*Uromyces cobresiae*	*Carex* sp.	Uzbekistan	[146]
*Uromyces collinus*	*Bauhinia* sp.	México	[292]
*Uromyces coluteae*	*Colutea arborescens*	Austria	[299]
*Uromyces combreti*	*Combretum* sp.	Myanmar	[405]
*Uromyces conicus*	*Cleome* sp.	Bolivia	[411]
*Uromyces correntinus*	*Rhynchospora tenuis*	Argentina	[415]
*Uromyces costesianus*	*Sphaeralcea velutina*	Chile	[386]
*Uromyces crepidis-fraasii*	*Crepisfraasii* sp.	Greece	[419]
*Uromyces cretensis*	*Coronilla parviflora* and *C*. *rostrata*	Greece	[421]
*Uromyces crotalariae-nitens*	*Crotalaria nitens*	Colombia	[423]
*Uromyces cruchetii*	*Borreria tenella*	Colombia	[226]
*Uromyces cucumivorus*	*Cucumis melo*	Iraq	[127]
*Uromyces cuenodii*	*Silene eriocalycina*	Iraq	[196]
*Uromyces cyanotidis*	*Cyanotis capitata*	Papua New Guinea	[426]
*Uromyces cyathulae*	*Cyathula globulifera*	Eritrea	[287]
*Uromyces dendroseridis*	*Dendroseris micrantha*	Chile	[240]
*Uromyces densus*	*Bidens pilosa*	Puerto Rico	[76,77]
*Uromyces desmodiicola*	*Desmodium albiflorum*	Brazil	[102]
*Uromyces desmodii-leiocarpi*	*Desmodium leiocarpum*	Brazil	[172]
*Uromyces dieramatis*	*Dierama* spp.	South Africa	[273]
*Uromyces dilucidus*	*Sisyrinchium striatum*	Argentina	[415]
*Uromyces diniensis*	*Ononis fruticosa*	France	[460]
*Uromyces dipcadi*	*Dipcadi viride*	Kenya	[487]
*Uromyces discariae*	*Discaria toumatou*	New Zealand	[313]
*Uromyces dispersus*	*Apios fortunei*	Japan	[222]
*Uromyces dobremezii*	*Euphorbia stracheyi*	Nepal	[464]
*Uromyces doebbeleri*	*Hypericum irazuense*	Costa Rica	[310]
*Uromyces dorystaechadis*	*Dorystaechas hastata*	Turkey	[471]
*Uromyces drimiopsidis*	*Drimiopsis maculata*	South Africa	[138]
*Uromyces dubiosus*	*Lantana* sp.	Goiás	[291]
*Uromyces ducellieri*	*Anabasis aphylla*	China	[91]
*Uromyces dusenii*	*Gilliesia graminea*, *G*. *monophylla*, *Miersia chilensis*, and *Ornithogalum biflorum*	Chile	[240]
*Uromyces echinodes*	*Asclepiadaceae*	Suriname	[473]
*Uromyces eclipsis*	*Zygophyllum morgsana*	South Africa	[234]
*Uromyces edwardsiae*	*Edwardsia* spp. and *Sophora* spp.	New Zealand	[284]
*Uromyces ehrhartae-giganteae*	*Ehrharta* spp.	South Africa	[429]
*Uromyces ellipticus*	*Glycyrrhiza astragalina*	Chile	[240]
*Uromyces ellisianus*	*Euphorbia marginata*	Minnesota	[277]
*Uromyces emmeorhizae*	*Emmeorhiza umbellata*	Venezuela	[478]
*Uromyces eriogoni*	*Eriogonum virgatum*	California	[388]
*Uromyces ermelensis*	*Indigofera* sp.	South Africa	[138]
*Uromyces erythrinae*	*Erythrina* sp.	Ecuador	[165]
*Uromyces euphlebius*	*Phoradendron calyculatus*	México	[15]
*Uromyces euphorbiae-javanicae*	*Euphorbia javanica*	Indonesia	[309]
*Uromyces euphorbiae-lunulatae*	*Euphorbia esula*, *E*. *Kansui*, and *E*. *lunulata*	China	[108,109]
*Uromyces evastigatus*	*Phthirusa pyrifolia*	El Salvador	[15]
*Uromyces fiebrigii*	*Bauhinia* sp.	Paraguay	[216]
*Uromyces fiorianus*	*Peucedanum fraxinifolium* and *Peucedanum* sp.	South Africa	[138]
*Uromyces flemmingiae*	*Flemingia* sp.	Uganda	[507]
*Uromyces fleuryae*	*Fleurya podocarpa*	Gabon	[508]
*Uromyces floralis*	*Bauhinia hiemalis*, *B*. *cuyabensis*, *B*. *Holophylla*, and *B*. *rufa*	Brazil	[102]
*Uromyces floscopae*	*Floscopa peruviana*	Brazil	[102]
*Uromyces fontii*	*Peplis acutangula*	Morocco	[510]
*Uromyces foveolatus*	*Bauhinia hirsuta* and *B*. *mirandina*	Brazil	[102]
*Uromyces fuscatus*	*Polygonum alpinum*	Idaho and Utah	[281]
*Uromyces fusisporus*	*Acacia neriifolia* and *A*. *salicina*	Australia	[177]
*Uromyces fremonti*	*Oenothera fremontii*	Kansas	[79]
*Uromyces galactiae*	*Galactia pedunculata*	Brazil	[517]
*Uromyces galii*	*Galium aparine* and *G*. *spurium*	Japan	[222]
*Uromyces galii-californici*	*Galium californicum* and *Galium* sp.	California	[520]
*Uromyces galphimiae*	*Galphimia glauca* and *G*. *humboldtiana*	México	[456]
*Uromyces garanbiensis*	*Ehretia dicksonii*	Taiwan	[521]
*Uromyces gaubae*	*Caltha introloba*	Australia	[522]
*Uromyces gausseni*	*Dorycnopsis gerardii*	France	[516]
*Uromyces geissorhizae*	*Geissorhiza* sp.	Western Cape Province	[523]
*Uromyces geraniicola*	*Geranium patagonicum*	Chile	[240]
*Uromyces gigantiformis*	*Bidens* sp.	Colombia	[423]
*Uromyces globosus*	*Sapium* spp.	México	[103]
*Uromyces gnaphalii*	*Gnaphalium* sp.	Colorado	[388]
*Uromyces goyazensis*	*Bauhinia* sp.	Brazil	[538]
*Uromyces grandiotii*	*Ancrumia cuspidata*	Chile	[540]
*Uromyces greenstockii*	*Ipomoea greenstockii*	South Africa	[138]
*Uromyces guayacuru*	*Statice brasiliensis*	Buenos Aires	[542]
*Uromyces habrochloae*	*Habrochloabul lockii*	Malawi	[244]
*Uromyces hainanicus*	*Ipomoea sumatrana*	China	[319]
*Uromyces handelii*	*Lotus gebelia*	Iraq	[220]
*Uromyces hardenbergiae*	*Hardenbergia monophylla*	Australia	[280]
*Uromyces hawksworthii*	*Phthirusa stelis*	Brazil	[60]
*Uromyces heimii*	*Medicago arborea*	France	[516]
*Uromyces hellebori-thibetani*	*Helleborus thibetanus*	China	[195]
*Uromyces hessii*	*Zantedeschia angustiloba*	Angola	[558]
*Uromyces heterantherae*	*Heteranthera reniformis*	Brazil	[102]
*Uromyces heterogeneus*	*Hibiscus syriacus*	India	[126]
*Uromyces heteromallus*	*Haloxylon recurvum*	Pakistan	[563]
*Uromyces hewittiae*	*Hewittia bicolor*	Philippines	[565]
*Uromyces hidakaensis*	*Pisum sativum*	Japan	[222]
*Uromyces himalaicus*	*Lilium* sp.	Nepal	[567]
*Uromyces holubii*	*Dracaena* sp.	Gauteng	[573]
*Uromyces huallagensis*	*Desmodium* sp.	Peru	[575]
*Uromyces hyderabadensis*	*Atylosia scarabaeoides*	India	[579]
*Uromyces hydrocotylicola*	*Hydrocotyle* sp.	China	[96]
*Uromyces hypericinus*	*Hypericum brasiliense*	Formosa	[180]
*Uromyces hypsophilus*	*Euphorbia* sp.	Mendoza	[391]
*Uromyces indicus*	*Sporobolus indicus*	Barbados	[589]
*Uromyces infarctus*	*Cayaponia* sp.	Costa Rica	[127]
*Uromyces inflatus*	*Anisotome* sp.	New Zealand	[591]
*Uromyces ingicola*	*Inga* sp.	Amazonas	[575]
*Uromyces ingiphilus*	*Inga edulis*	Argentina	[592]
*Uromyces insignis*	*Echinocephalum latifolium* and *Melanthera latifolia*	Brazil	[219]
*Uromyces insularis*	*Clitoria cajanifolia*	Puerto Rico	[466]
*Uromyces invisus*	*Solanum sisymbriifolium*	Argentina	[415]
*Uromyces ipatingae*	*Clitoria fairchildiana*	Brazil	[219]
*Uromyces isachnes*	*Isathne kunthiana*	Sri Lanka	[594]
*Uromyces jatrophicola*	*Cnidoscolus* sp. and *Jatropha* sp.	Brazil	[103]
*Uromyces juncicola*	*Juncus stipulatus*	Mendoza	[180]
*Uromyces johowii*	*Vicia macraei*, *V*. *nigricans*, and *Vicia* sp.	Chile	[282]
*Uromyces kentaniensis*	*Antholyza aethiopica* and *Chasmanthe aethiopica*	South Africa	[322]
*Uromyces kigesianus*	*Pittosporum abyssinicum*	Uganda	[609]
*Uromyces kochianus*	*Geranium nodosum*	Switzerland	[611]
*Uromyces koeleriae*	*Koeleria caucasica*	Russia	[106]
*Uromyces krantzbergensis*	*Anthericum* sp.	Namibia	[138]
*Uromyces kurtzii*	*Senecio* spp.	Argentina	[415]
*Uromyces kwangensis*	*Justicia* sp.	Congo	[218]
*Uromyces langtangensis*	*Anaphalis nepalensis*	Nepal	[463]
*Uromyces largus*	*Chamaesyce lata*	Colorado	[87,88]
*Uromyces* sp.	*Lasiocorys abyssinica*	Eritrea	[287]
*Uromyces latimammatus*	*Ipomoea sumatrana*	China	[621]
*Uromyces leonotidis*	*Leonotis nepetifolia*	India	[75]
*Uromyces lereddei*	*Colutea arborescens*	France	[220]
*Uromyces lespedezae*	*Lespedeza capitata*	Vermont	[583]
*Uromyces lespedezae-bicoloris*	*Lespedeza bicolor* and *L*. *formosa*	China	[108,109]
*Uromyces lespedezae-macrocarpae*	*Campylotropis macrocarpa*, *Lespedeza bicolor*, and *L*. *formosa*	China	[108,109]
*Uromyces lespedezae-sericeae*	*Lespedeza sericea*	Pakistan	[208]
*Uromyces libycus*	*Lotus pusillus*	Libya	[197]
*Uromyces lomandracearum*	*Lomandra longifolia*	Australia	[62]
*Uromyces loranthi*	*Loranthus* sp.	Brazil	[376]
*Uromyces lotononidicola*	*Lotononis cytisoides*	South Africa	[632]
*Uromyces lygei*	*Lygeum spartum*	Sardegna	[325]
*Uromyces macnabbii*	*Chionochloa* spp. and *Danthonia* spp.	New Zealand	[591]
*Uromyces mangenotii*	*Vicia pubescens*	France	[516]
*Uromyces manihoticola*	*Manihot* spp.	Brazil	[103]
*Uromyces manihotis-catingae*	*Manihot* spp.	Brazil	[102]
*Uromyces marinus*	*Medicago marina*	Morocco	[220]
*Uromyces martinii*	*Melanthera* spp. and *Bidens* spp.	Florida	[262]
*Uromyces massoniae*	*Massonia latifolia*	South Africa	[138]
*Uromyces megalosporus*	*Tessaria absinthioides*	Tucumán	[371]
*Uromyces melandrii*	*Melandrium cucubaloides*	Los Lagos	[162]
*Uromyces melasphaerulae*	*Melasphaerula graminea*	Western Cape Province	[424]
*Uromyces melothriae*	*Melothria tomentosa*	Eritrea	[287]
*Uromyces meygounensis*	*Euphorbia bungei*	Iran	[638]
*Uromyces miersiae*	*Miersia chilensis*	Chile	[540]
*Uromyces mikaniae*	*Mikania* sp.	Brazil	[219]
*Uromyces mimusops*	*Mimusops* sp.	South Africa	[700]
*Uromyces moehringiae*	*Moehringia lateriflora*	Japan	[297]
*Uromyces mongolicus*	*Euphorbia kozlovii*	Mongolia	[535]
*Uromyces montis-ferrati*	*Euphorbia luteola*	Northern Africa	[649]
*Uromyces moraeae*	*Moraea spathacea*	South Africa	[144]
*Uromyces mussooriensis*	*Stipa sibirica*	India	[106]
*Uromyces myosotidis*	*Myosotis* sp.	Turkey	[470]
*Uromyces myristicus*	*Euphorbia bicolor*	Texas	[657]
*Uromyces namaqualandus*	*Roepera cordifolia*	Namibia	[384]
*Uromyces nassauviae*	*Nassauvia lagascae*	Argentina	[415]
*Uromyces nassellae*	*Nassell apubiflora*	Bolivia	[106]
*Uromyces natricis*	*Ononis rotundifolia*	France	[342]
*Uromyces nattrassii*	*Statice spicata*	Cyprus	[350]
*Uromyces naucinus*	*Cayaponia* sp.	Ecuador	[127]
*Uromyces nevadensis*	*Primula suffrutescens*	California	[78]
*Uromyces notabilis*	*Cyperus* sp. and *Kyllinga* sp.	Uganda	[487]
*Uromyces nothoscordi*	*Nothoscordum striatum*	Texas	[660]
*Uromyces numidicus*	*Geranium atlanticum*	Northern Africa	[649]
*Uromyces nymphoidis*	*Nymphoides peltata*	Romania	[228]
*Uromyces oberwinklerianus*	*Acalypha* sp.	Costa Rica	[310]
*Uromyces oblectaneus*	*Rhynchospora corymbosa* and *R*. *exaltata*	Brazil	[219]
*Uromyces obscurus*	*Phaseolus* sp.	México	[456]
*Uromyces ocimi*	*Ocimum menthifolium*	Uganda	[168]
*Uromyces oedipus*	*Sophora japonica*	Japan	[665]
*Uromyces oenotherae*	*Oenothera linifolia*	Illinois	[582]
*Uromyces oliveirae*	*Bellevalia eigii*	Israel	[390]
*Uromyces ophiorrhizae*	*Ophiorrhiza longiflora*	Indonesia	[309]
*Uromyces orchidearum*	*Chiloglottis* spp.	Australia	[268]
*Uromyces ornatipes*	*Phrygilanthus sonorae*	México	[15]
*Uromyces ornithopodioides*	*Ornithopus isthmocarpus* and *O*. *compressus*	Portugal	[317]
*Uromyces orthosiphonis*	*Orthosiphon glabratus*	India	[152]
*Uromyces otakou*	*Poa* spp.	New Zealand	[591]
*Uromyces otaviensis*	*Ipomoea verbascoidea*	Namibia	[385]
*Uromyces ovalis*	*Leersia oryzoides*	Japan	[671]
*Uromyces ovirensis*	*Primula wulfeniana*	Austria	[672]
*Uromyces pannosus*	*Bauhinia candicans*	Brazil	[216]
*Uromyces papillatus*	*Heteromorpha arborescens*	South Africa	[137]
*Uromyces parilis*	*Rumex occultans*	Israel	[81]
*Uromyces paspalicola*	*Paspalum racemosum*	Ecuador	[294]
*Uromyces pavgii*	*Achyranthes aspera*	India	[677]
*Uromyces pavoniae*	*Pavonia racemosa*	Puerto Rico	[259]
*Uromyces pazschkeanus*	*Vigna* sp.	Eritrea	[287]
*Uromyces penniseti*	*Pennisetum lanatum*	Pakistan	[536,679]
*Uromyces pentaceae*	*Pentace burmanica*	India	[680]
*Uromyces pentaschistidis*	*Pentaschistis airoides*	South Africa	[243]
*Uromyces peraffinis*	*Bauhinia* sp.	Brazil	[219]
*Uromyces perlebiae*	*Bauhinia* spp.	Brazil	[102]
*Uromyces phalaridicola*	*Phalaris minor*	Turkmenistan	[106]
*Uromyces phaseolicola*	*Phaseolus prostratus*	Argentina	[126]
*Uromyces phlogacanthi*	*Phlogacanthus celebicus*	Indonesia	[309]
*Uromyces phtirusae*	*Phthirusa pyrifolia*	Colombia	[15]
*Uromyces phyllachoroides*	*Cynosurus elegans*	Tunisia	[684]
*Uromyces physanthyllidis*	*Physanthyllis tetraphylla*	Greece	[544]
*Uromyces pittospori*	*Pittosporum abyssinicum*	Eritrea	[287]
*Uromyces planiusculus*	*Rumex frutescens*	Tristan da Cunha	[688]
*Uromyces plantaginis*	*Plantago barbata* and *P*. *tubulosa*	Argentina	[415]
*Uromyces poiretiae*	*Poiretia scandens*	Venezuela	[431]
*Uromyces polemanniae*	*Polemannia* spp.	South Africa	[142]
*Uromyces poliotelis*	*Anguria* sp., *Gurania* sp. and *Selysia prunifera*	Costa Rica	[127]
*Uromyces politus*	*Muehlenbeckia cunninghamii*	Australia	[343]
*Uromyces polygoni-avicularis*	*Polygonum nepalense*	Nepal	[693]
*Uromyces polytriadicola*	*Polytrias amaura*	Philippines	[232]
*Uromyces poonensis*	*Sesbania aegyptiaca*, *S. grandiflora*, and *S*. *sesban*	India	[357]
*Uromyces porcensis*	*Inga ingoides*	Colombia	[226]
*Uromyces porosus*	*Vicia americana* and *V*. *sparsifolia*	Iowa	[155]
*Uromyces pozoae*	*Pozoa hydrocotylifolia*	Chile	[240]
*Uromyces pratensis*	*Poa pratensis*, *Ranunculus auricomus*, and *R*. *cassubicus*	Finland	[329]
*Uromyces prismaticus*	*Secale montanum*	Iran	[320]
*Uromyces procerus*	*Festuca procera*	Chile	[240]
*Uromyces pseudarthriae*	*Pseudarthria robusta*	South Africa	[700]
*Uromyces psychotriae*	*Psychotria* sp.	Brazil	[219]
*Uromyces pulvinatus*	*Euphorbia inaequilatera*	South Africa	[702]
*Uromyces quaggafonteinus*	*Ehrharta calycina*	South Africa	[429]
*Uromyces ramacharii*	*Ocimum* sp.	India	[706]
*Uromyces rapaneae*	*Rapanea* sp.	São Paulo	[450]
*Uromyces ratoides*	*Cayaponia* sp.	Ecuador	[212]
*Uromyces ratus*	*Cayaponia* spp.	Brazil	[212]
*Uromyces rayssiae*	*Scilla hyacinthoides*	Israel	[390]
*Uromyces rebeccae*	*Suaeda californica*	California	[47]
*Uromyces regius*	*Bauhinia candicans*	Brazil	[219]
*Uromyces reichei*	*Milla bivalvis* and *Triteleia gaudichaudiana*	Chile	[415]
*Uromyces reichertii*	*Scilla hyacinthoides* and *Hordeum bulbosum*	Israel	[81]
*Uromyces reynoldsii*	*Modeccabracteata* and *Trichosanthes* spp.	Myanmar	[212]
*Uromyces riloensis*	*Doronicum cordifolium*	Bulgaria	[82]
*Uromyces rostratus*	*Eriosema* sp.	Rio de Janeiro	[331]
*Uromyces rubidus*	*Andropogon condensatus*	Brazil	[199]
*Uromyces rugosus*	*Lupinus* sp.	México	[398]
*Uromyces rugulosus*	*Campylotropis* spp. and *Lespedeza* spp.	China	[96]
*Uromyces ruiz-leali*	*Anarthrophyllum elegans*	Argentina	[415]
*Uromyces rzedowskii*	*Ledenbergia macrantha*	México	[292]
*Uromyces sakawensis*	*Solidago virgaurea*	Japan	[711]
*Uromyces sasaensis*	*Valerianakilimandscharica* and *V. volkensii*	Uganda	[712]
*Uromyces satarensis*	*Blainville acmella* and *B*. *latifolia*	China and India	[319]
*Uromyces saulensis*	*Selysia prunifera*	France	[127]
*Uromyces scaberulus*	*Lespedeza bicolor*, *L. cuneate*, *L*. *cyrtobotrya*, and *L*. *formosa*	China	[156]
*Uromyces scirpinus*	*Scirpus supinus*	Philippines	[232]
*Uromyces secamones*	*Secamone platystigma*	Uganda	[168]
*Uromyces sedi*	*Sedum anacampseros*	France	[717]
*Uromyces seligeri*	*Lathyrus grandiflorus* and *L*. *sylvestris*	Greece and Russia	[421]
*Uromyces sellierae*	*Selliera radicans*	New Zealand	[67]
*Uromyces semnanensis*	*Astragalus fridae*	Iran	[276]
*Uromyces senecionicola*	*Cacalia* sp. and *Senecio roldana*	México	[292]
*Uromyces senecionis-gigantis*	*Senecio gigas*	Ethiopia	[719]
*Uromyces seselis*	*Seseli tortuosum*	Portugal	[722]
*Uromyces sesseae*	*Sessea* sp.	Ecuador	[325]
*Uromyces shahrudensis*	*Onobrychis* sp.	Iran	[723]
*Uromyces shikokianus*	*Cladrastis platycarpa* and *C*. *shikokiana*	Japan	[222]
*Uromyces silenes-chloraefoliae*	*Silene chlorifolia*	Iran	[320]
*Uromyces silksvleyensis*	*Bartholina burmanniana*	Western Cape and South Africa	[378]
*Uromyces simulans*	*Vilfa* sp.	Colorado	[724]
*Uromyces siphocampyli-gigantei*	*Siphocampylus giganteus*	Ecuador	[725]
*Uromyces smilacis*	*Smilax* sp.	Colombia	[227]
*Uromyces snowdeniae*	*Snowdenia scabra*	Kenya	[140]
*Uromyces solariae*	*Solaria miersioides*	Chile	[240]
*Uromyces solidaginis-caricis*	*Carex varia*	Indiana	[728]
*Uromyces solidus*	*Desmodium strictum*	North Carolina	[657]
*Uromyces sonorensis*	*Merremia palmeri*	México	[729]
*Uromyces sophorae-japonicae*	*Sophora japonica*	Japan	[92]
*Uromyces sophorae-viciifoliae*	*Sophora viciifolia*	China	[732]
*Uromyces sparaxidis*	*Sparaxis lineata* and *S*. *tricolor*	South Africa	[142]
*Uromyces sphaericus*	*Perymenium ecuadoricum*	Ecuador	[210]
*Uromyces sphaerophysae*	*Swainsona salsula*	China	[198]
*Uromyces splendens*	*Astragalus oroboides*	Norway	[735]
*Uromyces sporoboloides*	*Sporobolus berteroanus*	Ecuador	[737]
*Uromyces standleyanus*	*Gaudichaudia schiedeana*	El Salvador	[301]
*Uromyces statices*	*Statice* sp.	California	[598]
*Uromyces statices-mucronatae*	*Statice mucronata*	Morocco	[279]
*Uromyces steironematis*	*Spartina michauxiana*	Nebraska	[738]
*Uromyces stellariae*	*Stellaria kotschyana*	Iran	[739]
*Uromyces stellariae-saxatilis*	*Stellaria media*, *S*. *saxatilis*, and *S*. *vestita*	China	[96]
*Uromyces stenorrhynchi*	*Stenorrhynchus* sp.	Peru	[740]
*Uromyces stipinus*	*Stipa rubens*	Russia	[106]
*Uromyces strauchii*	*Clutia daphnoides*	Southern Africa	[138]
*Uromyces struthanthi*	*Struthanthus* sp.	Panama	[15]
*Uromyces substriatus*	*Lupinus argenteus*	Montana	[79]
*Uromyces superstomatalis*	*Cayaponia rigida*	France	[127]
*Uromyces tarapotensis*	*Camptosema* sp.	Peru	[575]
*Uromyces teheranicus*	*Trifolium retense*	Iran	[744]
*Uromyces tehuelches*	*Alstroemeria patagonica*	Argentina	[167]
*Uromyces tener*	*Manettia gracilis*	Brazil	[219]
*Uromyces tepicensis*	*Loeselia amplectens*	México	[292]
*Uromyces thelymitrae*	*Thelymitra antennifera* and *T*. *flexuosa*	Australia	[81]
*Uromyces thermopsidicola*	*Thermopsis chinensis*	Japan	[222]
*Uromyces tolerandus*	*Manihot esculenta*	Brazil	[219]
*Uromyces tomentellus*	*Leguminosae* sp.	California	[747]
*Uromyces tosensis*	*Commelina communis*	Japan	[711]
*Uromyces tournefortiae*	*Tournefortia* sp.	Brazil	[600]
*Uromyces transcaspicus*	*Astragalus angustidens*	Turkmenistan	[748]
*Uromyces traucoensis*	*Selliera radicans*	Chile	[749]
*Uromyces triandrae*	*Themeda triandra*	India	[669]
*Uromyces trichoclines*	*Trichocline polymorpha*	Brazil	[219]
*Uromyces tricholenae*	*Tricholaena rosea*	Dominican Republic	[750]
*Uromyces tricorynes*	*Tricoryne elatior*	Australia	[245]
*Uromyces trigonellae-occultae*	*Trigonella occulta*	Egypt	[752]
*Uromyces tripogonicola*	*Tripogon lisboae*	Maharashtra	[753]
*Uromyces triteleiae*	*Brodiaea porrifolia*, *Leucocoryne alliacea*, and *Triteleia porrifolia*	Chile	[415]
*Uromyces trollipii*	*Roepera foetida* and *Zygophyllum foetidum*	South Africa	[385]
*Uromyces truncatulus*	*Geranium versicolor*	Greece	[421]
*Uromyces tulipae*	*Tulipa edulis*	Japan	[756]
*Uromyces tungurahuensis*	*Aspilia lanceolata*	Ecuador	[757]
*Uromyces uleanus*	*Euphorbia* sp.	Brazil	[219]
*Uromyces umiamensis*	*Cucumis* sp. and *Momordica cochinchinensis*	India	[127]
*Uromyces undulatoparietis*	*Ligularia hookeri*	China	[108,109]
*Uromyces unioniensis*	*Desmodium* sp. and *Meibomia* sp.	Brazil	[394]
*Uromyces unitus*	On living leaves of *Calandrinia leeana*	Washington	[758]
*Uromyces urariae*	*Uraria lagopodioides*	China	[759]
*Uromyces urgineae*	*Urginea indica*	India	[397]
*Uromyces valerianae-microphyllae*	*Valeriana microphylla*	Ecuador	[766]
*Uromyces vankyorum*	*Atriplex lampa*	Argentina	[282]
*Uromyces venustus*	*Cestrum nitidum*	México	[375]
*Uromyces verus*	*Bauhinia rufa*	Brazil	[100]
*Uromyces vesiculosus*	*Zygophyllum* spp.	Australia	[245]
*Uromyces viciae-unijugae*	*Vicia unijuga*	Japan	[92]
*Uromyces vicinus*	*Ipomoea* sp.	Brazil	[219]
*Uromyces vicosensis*	*Bauhinia* sp.	Brazil	[219]
*Uromyces viegasii*	*Bauhinia forficata* and *Bauhinia* sp.	Brazil	[219]
*Uromyces viennot-bourginii*	*Bellevalia eigii* and *Hordeum spontaneum*	Israel	[81]
*Uromyces vignae-luteolae*	*Vigna luteola*	Congo	[610]
*Uromyces visci*	*Viscum congolense*	Rwanda	[551]
*Uromyces volkartii*	*Trisetum flavescens*	Switzerland	[770]
*Uromyces vulpiae*	*Vulpia broteri*	Portugal	[722]
*Uromyces waipoua*	*Hypericum gramineum* and *H*. *japonicum*	New Zealand	[313]
*Uromyces wedeliae-biflorae*	*Wedelia biflora*	Indonesia	[309]
*Uromyces wellingtonicus*	*Sporobolus indicus*	India	[629]
*Uromyces wolfii*	*Borreria laevis*	Venezuela	[609]
*Uromyces wulffiae*	*Wulffia baccata*, *W*. *maculata*, *W*. *scandens*, and *W*. *stenoglossa*	Brazil	[219]
*Uromyces yoshinagae*	*Pisum sativum*	Honshu	[360]
*Uromyces zeyheri*	*Ixia scillaris*, *I. scillaris*, and *Tritonia pallida*	South Africa	[123]
*Uromyces zigadeni*	*Zigadenus paniculatus*	Utah	[328]
*Uromyces zizaniae-latifoliae*	*Zizania latifolia*	Taiwan	[773]

## Data Availability

Not applicable.
